# Supplementary material for: Three previously unrecognised classes of biosynthetic enzymes revealed during the production of xenovulene A
Source: Nat Commun. 2018 May 17;9:1963. doi: 10.1038/s41467-018-04364-9 (PMC5958101; doi:10.1038/s41467-018-04364-9)
Supplement: Supplementary file 1 — Supplementary Information [file 41467_2018_4364_MOESM1_ESM.pdf]

Three Previously Unrecognised Classes of Biosynthetic Enzymes Revealed During the Production of Xenovulene A

Coresponding Author russell.cox@oci.uni-hannover.de

Supplementary Information

**Supplementary Figures**	**2**
**Supplementary Tables**	**40**
**Supplementary Methods**	**55**
**Supplementary References**	**60**

Supplementary Figures

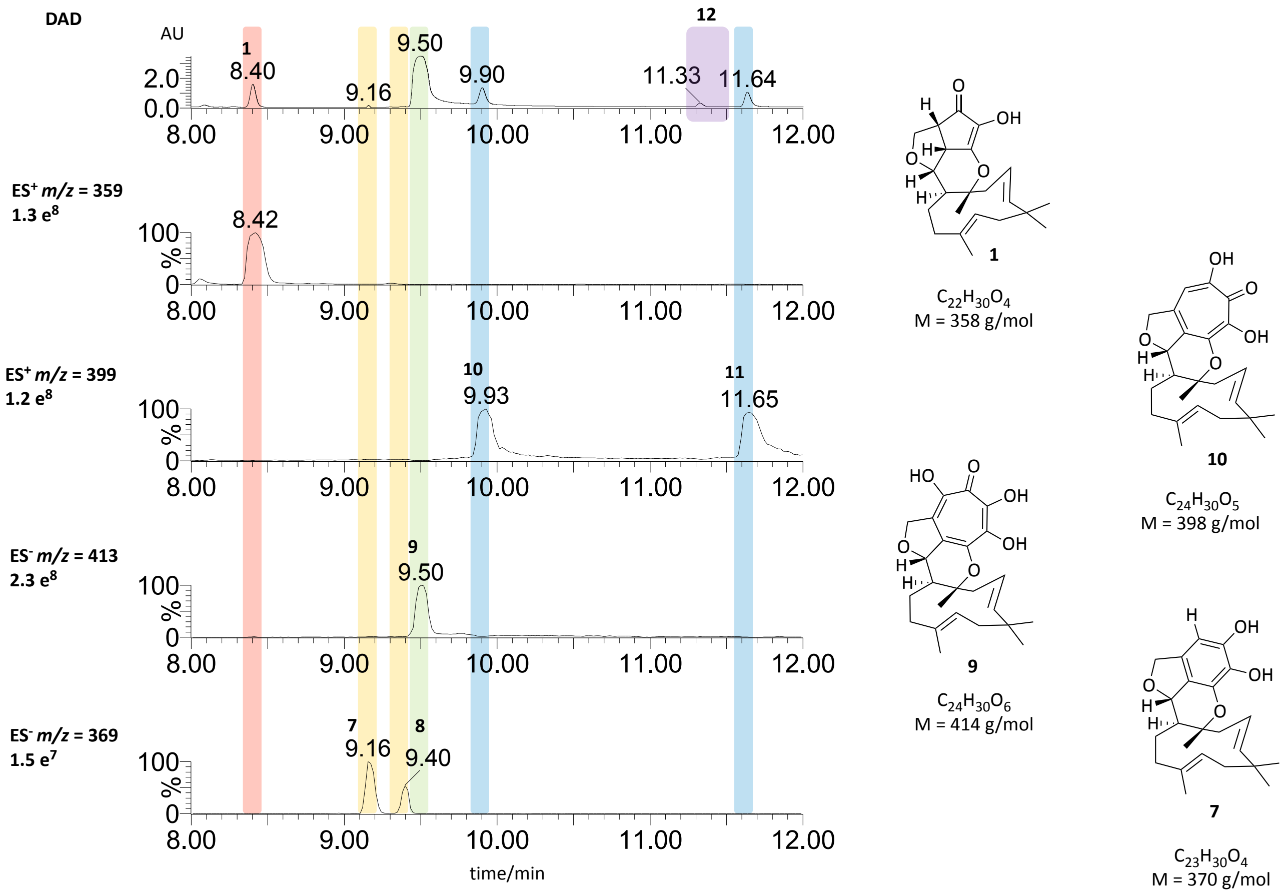

Supplementary Figure 1. LCMS (liquid chromatography mass spectrometry) chromatograms of an extract of WT *S. schorii* grown under **1**-production conditions. Top trace shows DAD (Dioode array detector) data (200-600 nm), all other traces show extracted ion chromatograms (ES-) at indicated *m/z* values.

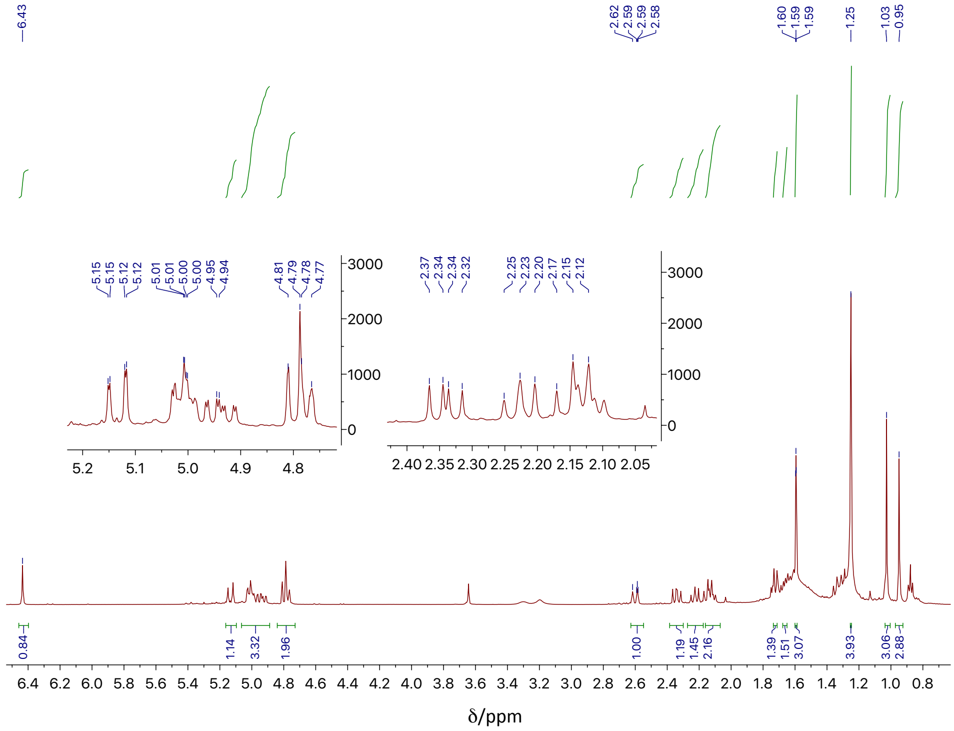

Supplementary Figure 2. ^{1}H NMR of compound **7** in $CDCl_3$ (500 MHz) referenced to $CDCl_3$. See Supplementary Table 1 for assignment.

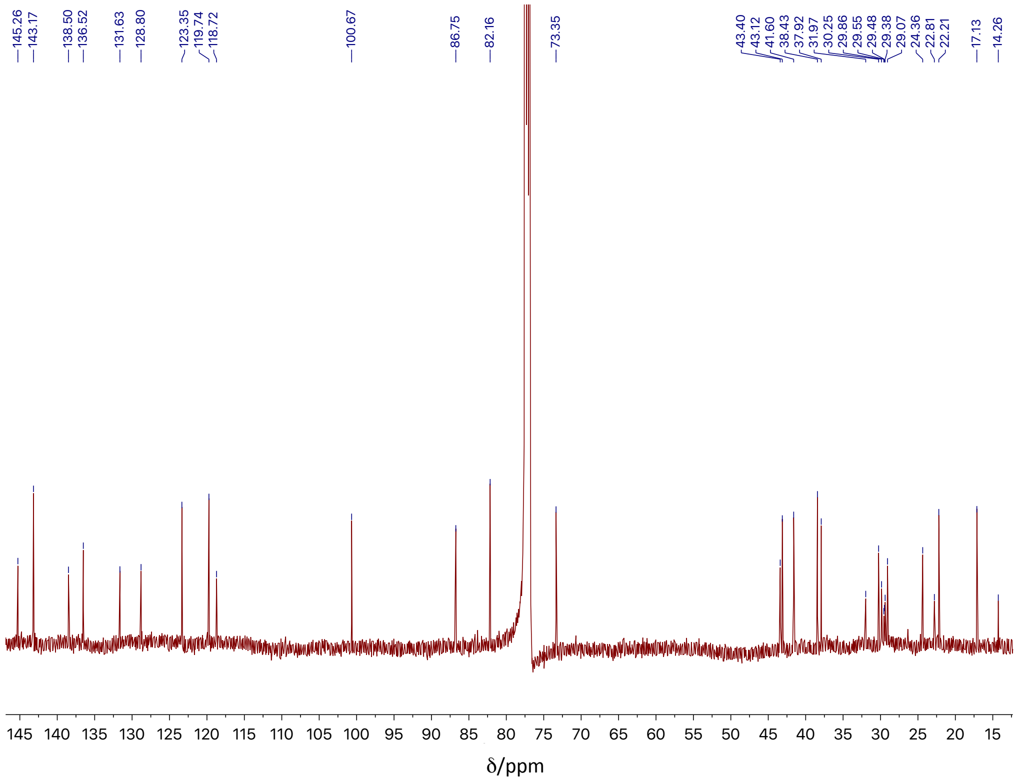

Supplementary Figure 3. ^{13}C NMR of compound **7** in $CDCl_3$ (500 MHz) referenced to $CDCl_3$. See Supplementary Table 1 For Assignment.

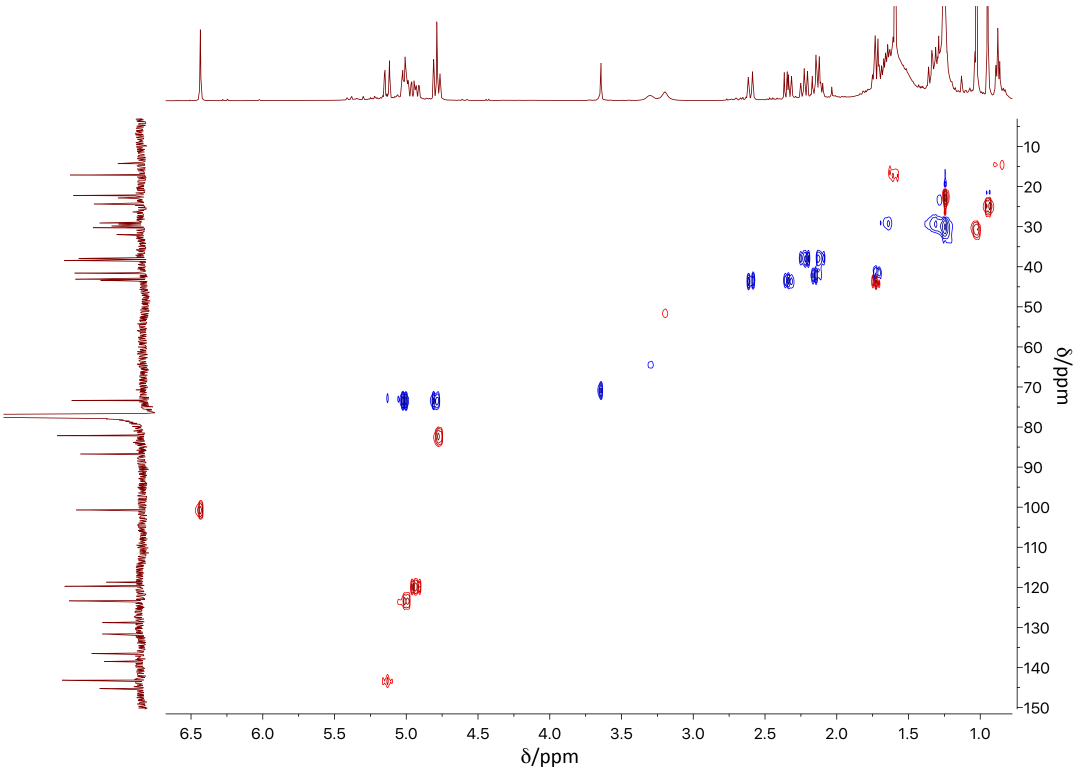

Supplementary Figure 4. HSQC of compound **7**.

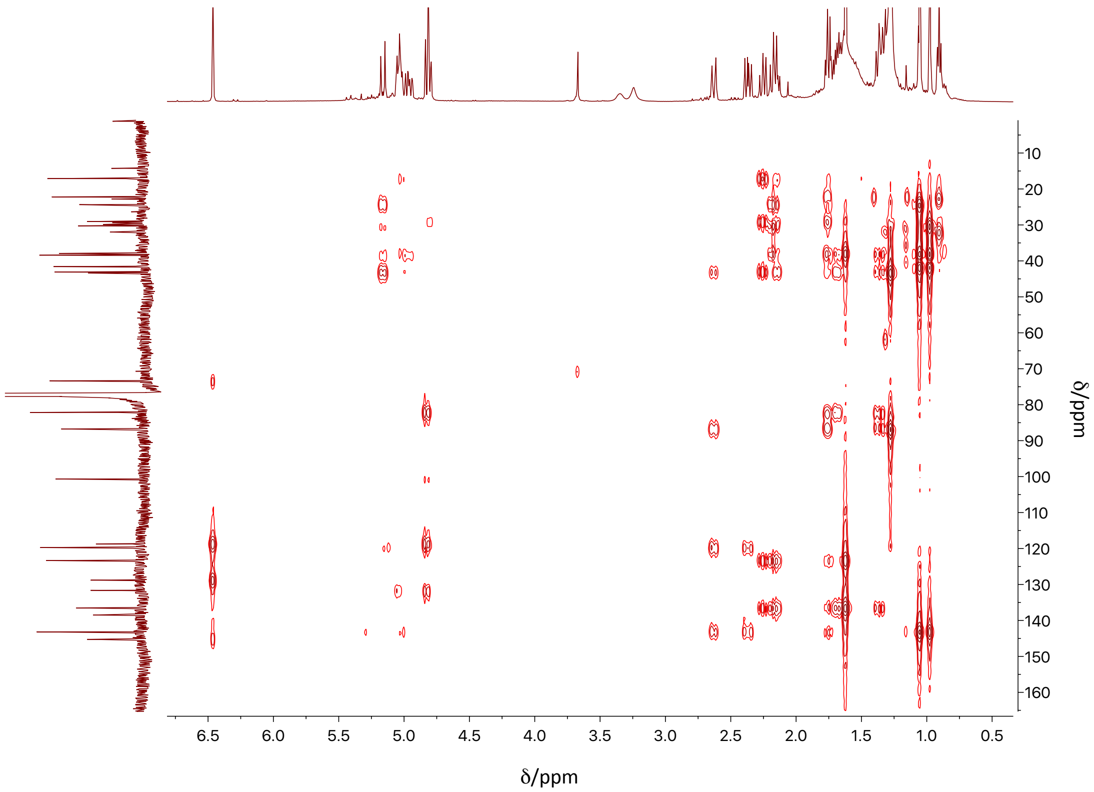

Supplementary Figure 5. HMBC of compound **7**.

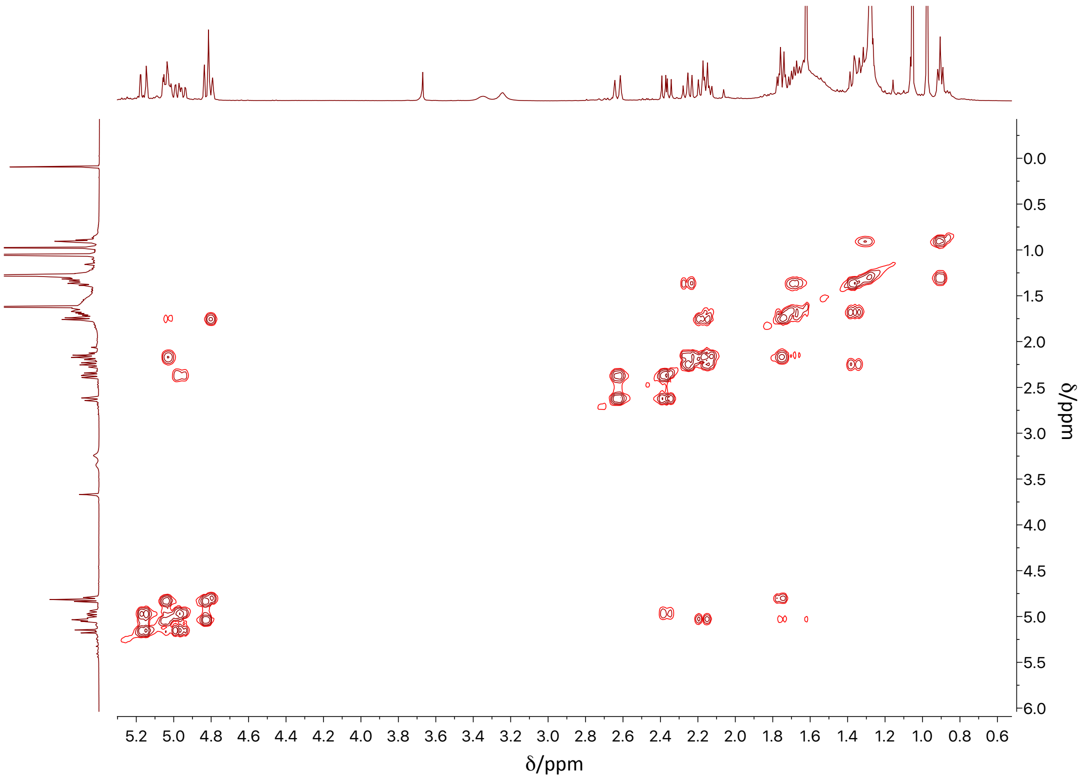

Supplementary Figure 6. ^{1}H,^{1}H COSY of compound **7**.

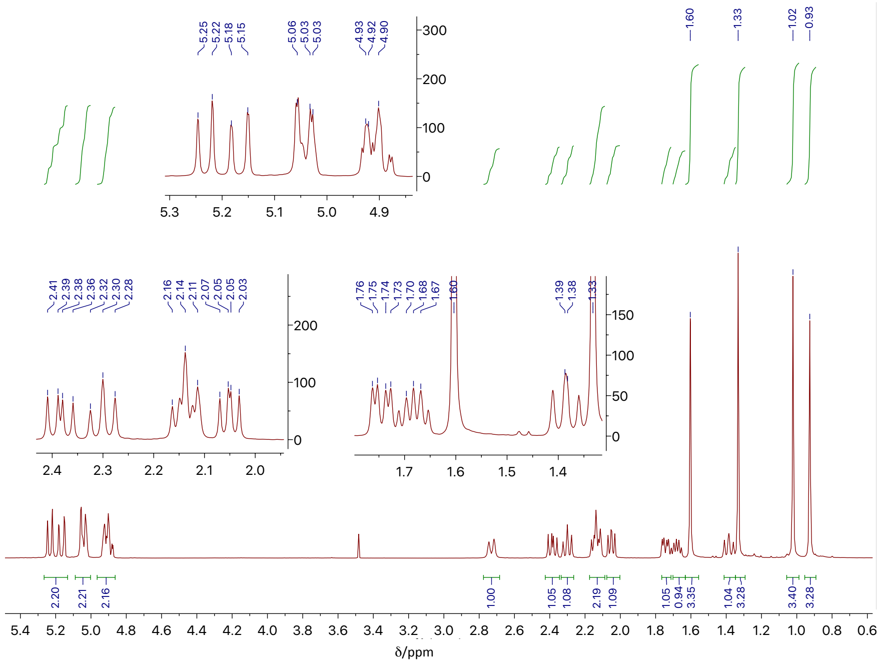

Supplementary Figure 7. ^{1}H NMR of compound **9** in $CDCl_3$ (500 MHz) referenced to $CDCl_3$. See Supplementary Table 2 for assignment.

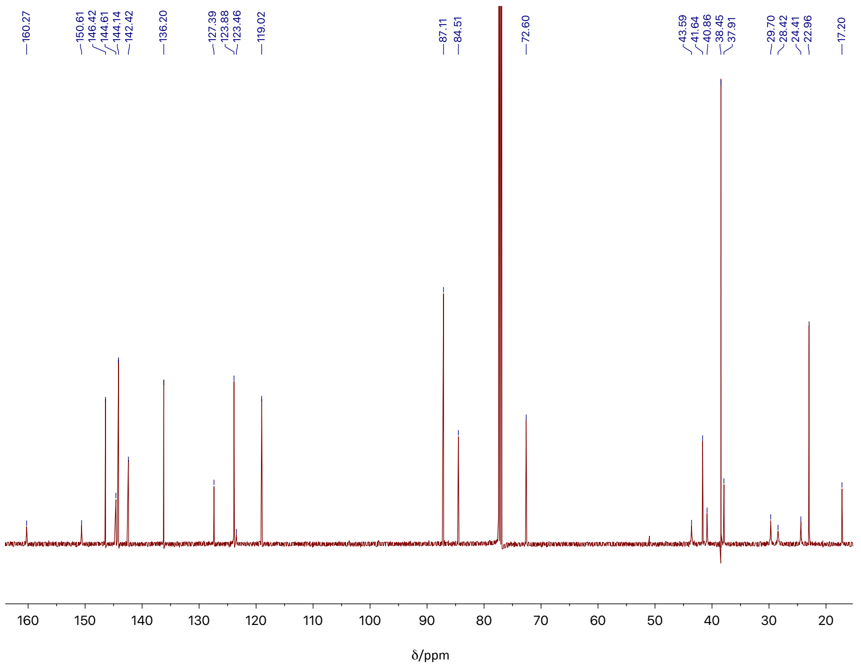

Supplementary Figure 8. ^{13}C NMR of compound **9** in $CDCl_3$ (125 MHz) referenced to $CDCl_3$. See Supplementary Table 2 for assignment.

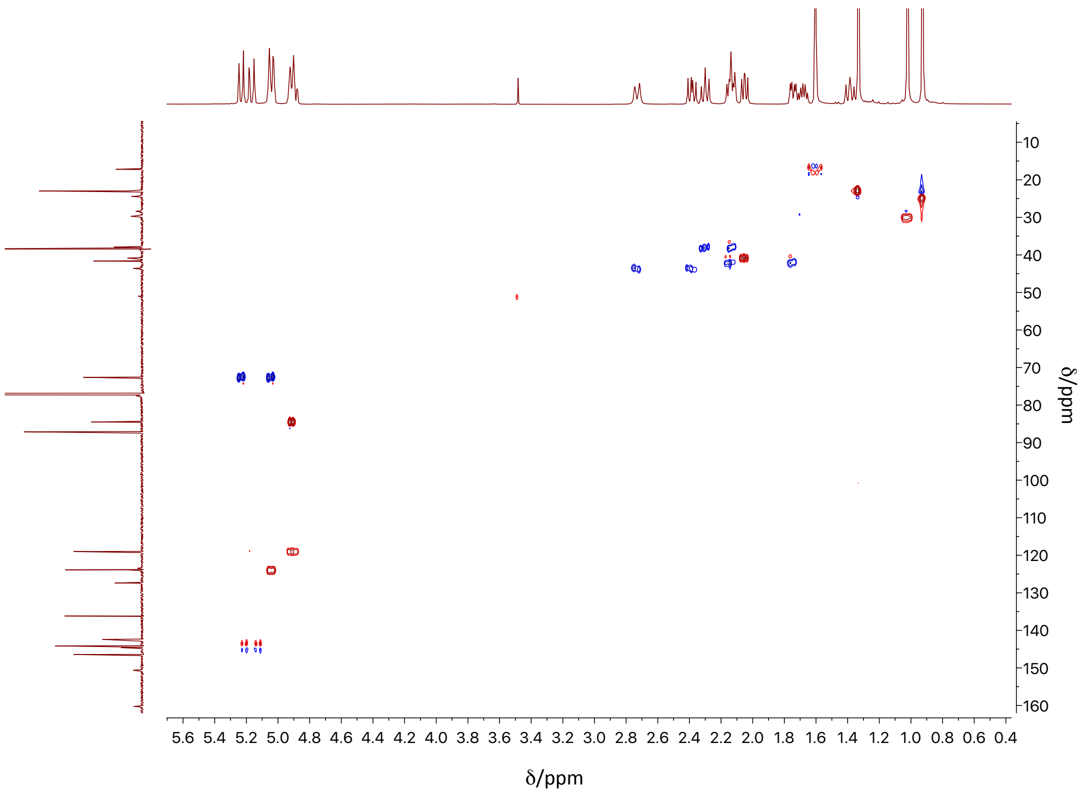

Supplementary Figure 9. HSQC of compound **9**.

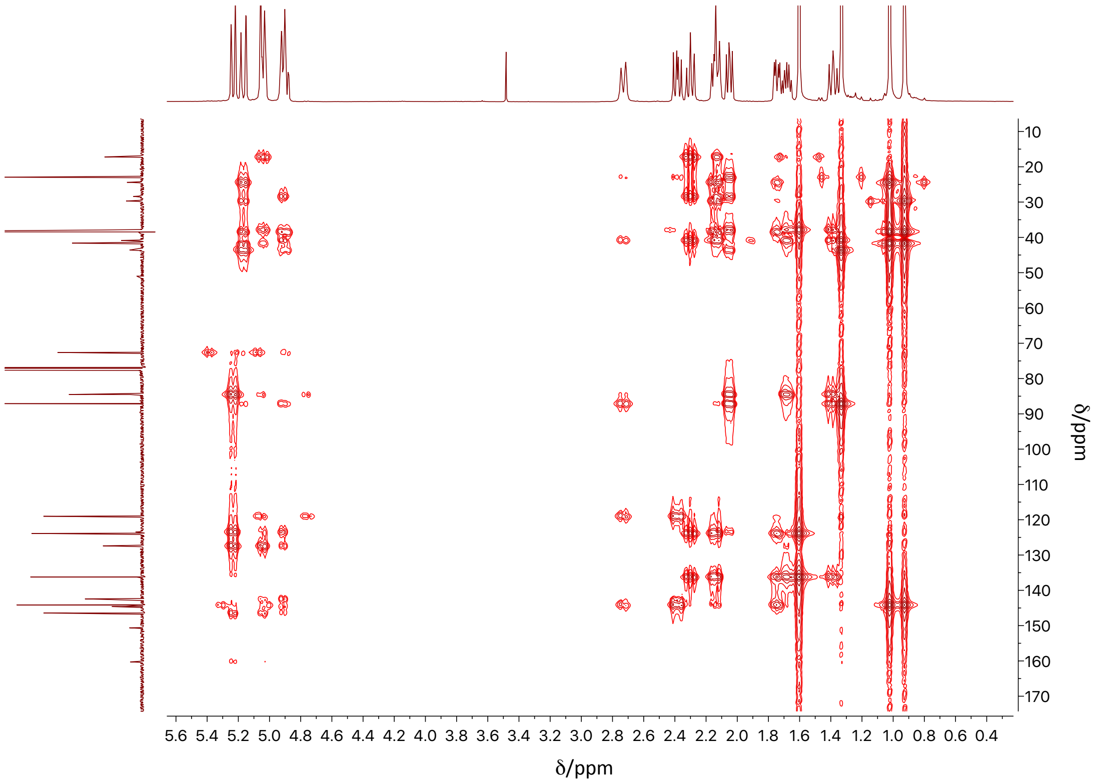

Supplementary Figure 10. HMBC of compound **9**.

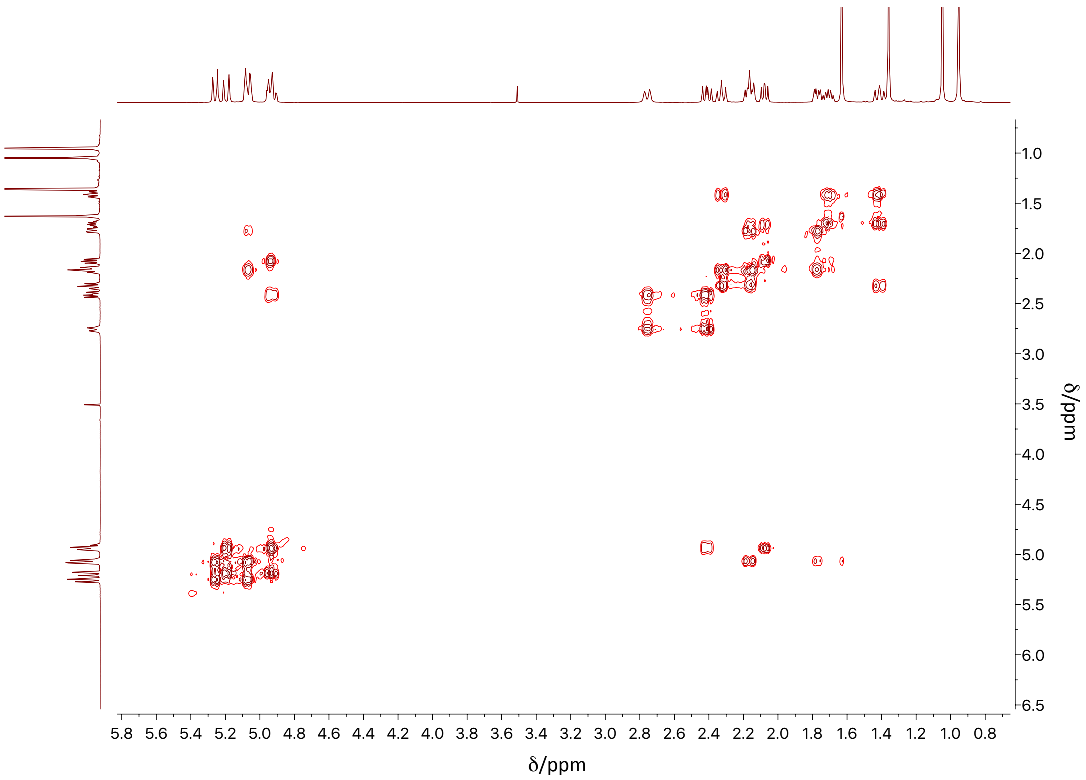

Supplementary Figure 11. $^{1}H,^{1}H$ COSY of compound **9**.

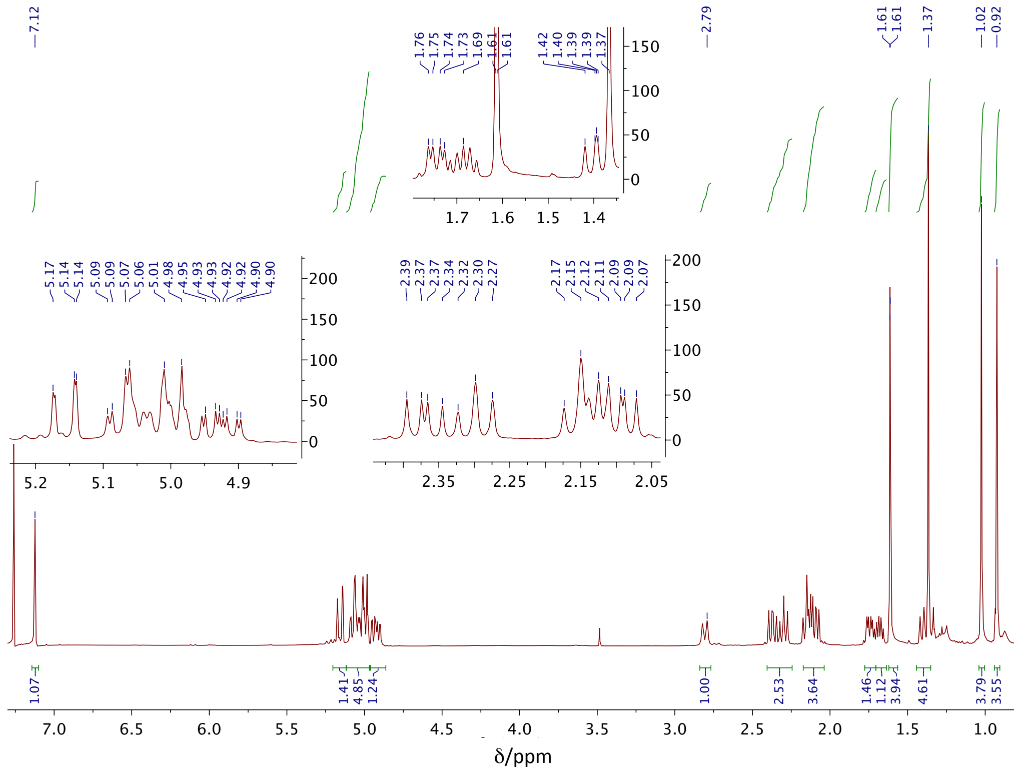

Supplementary Figure 12. ^{1}H NMR of compound **10** in $CDCl_3$ (500 MHz) referenced to $CDCl_3$. See Supplementary Table 3. for assignment.

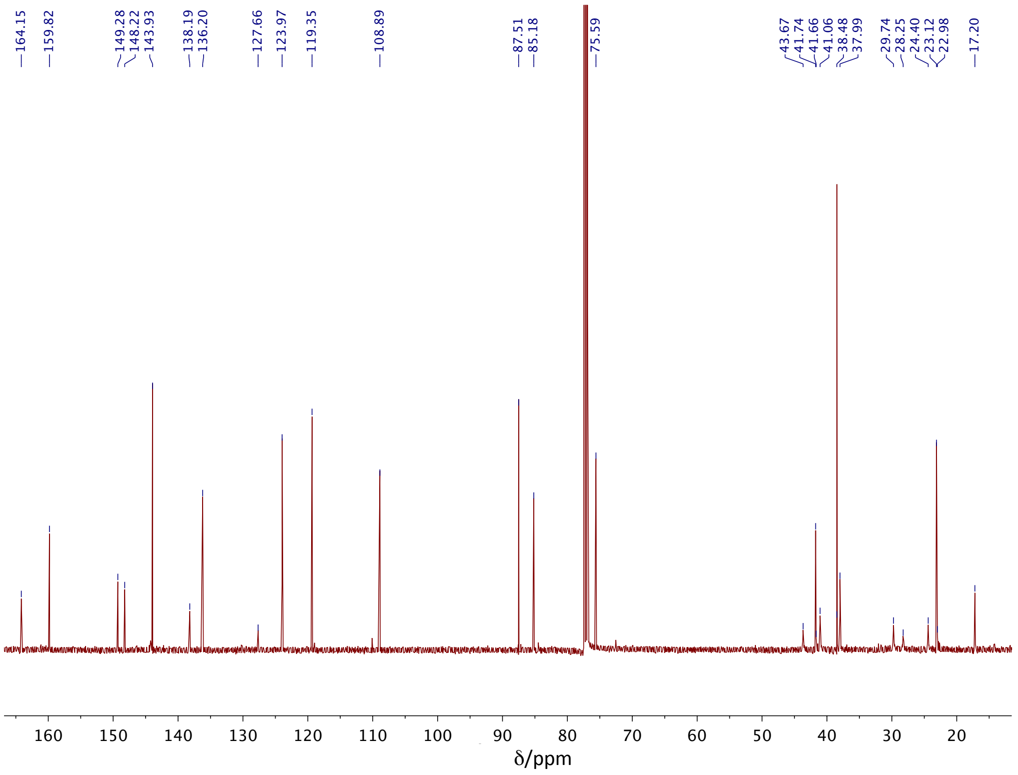

Supplementary Figure 13. ^{13}C NMR of compound **10** in $CDCl_3$ (500 MHz) referenced to $CDCl_3$. See Supplementary Table 3 for assignment.

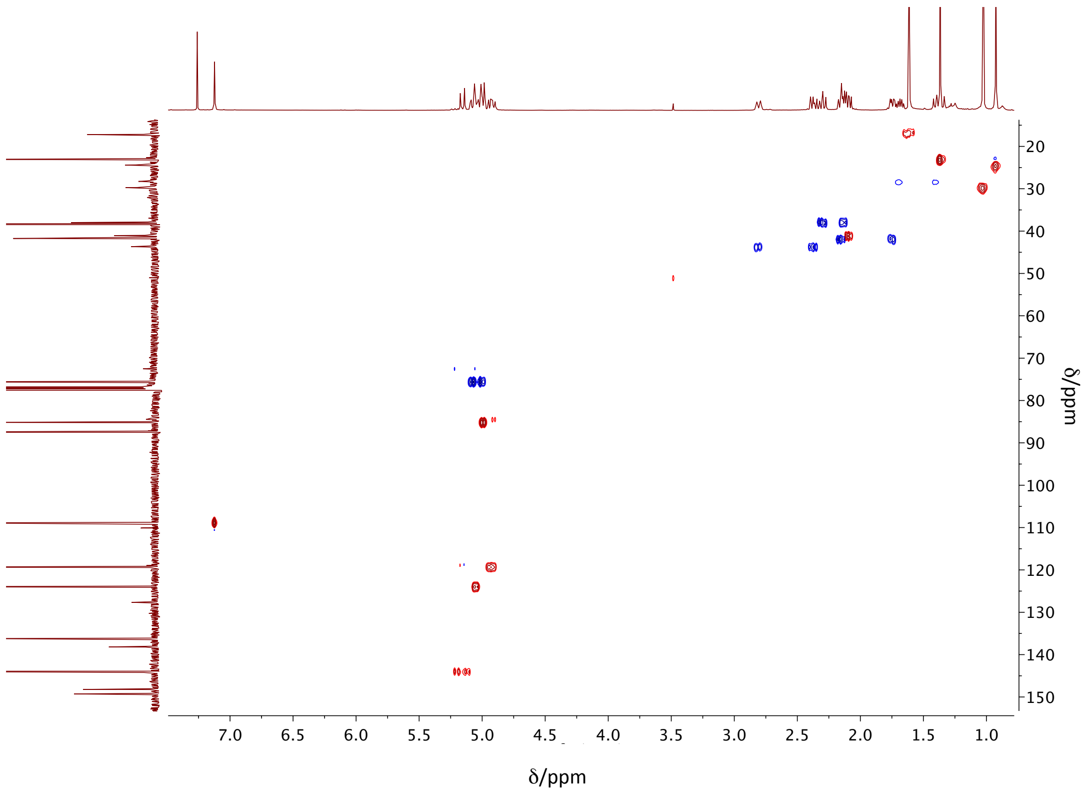

Supplementary Figure 14. HSQC of compound **10**.

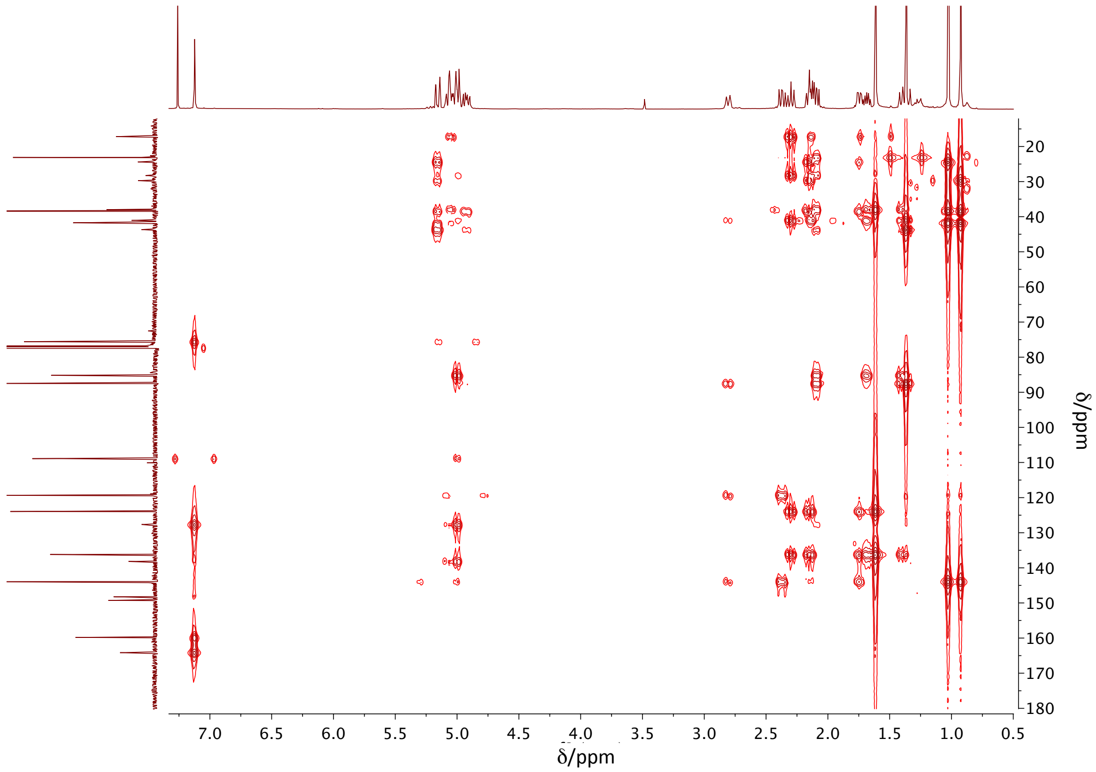

Supplementary Figure 15. HMBC of compound **10**.

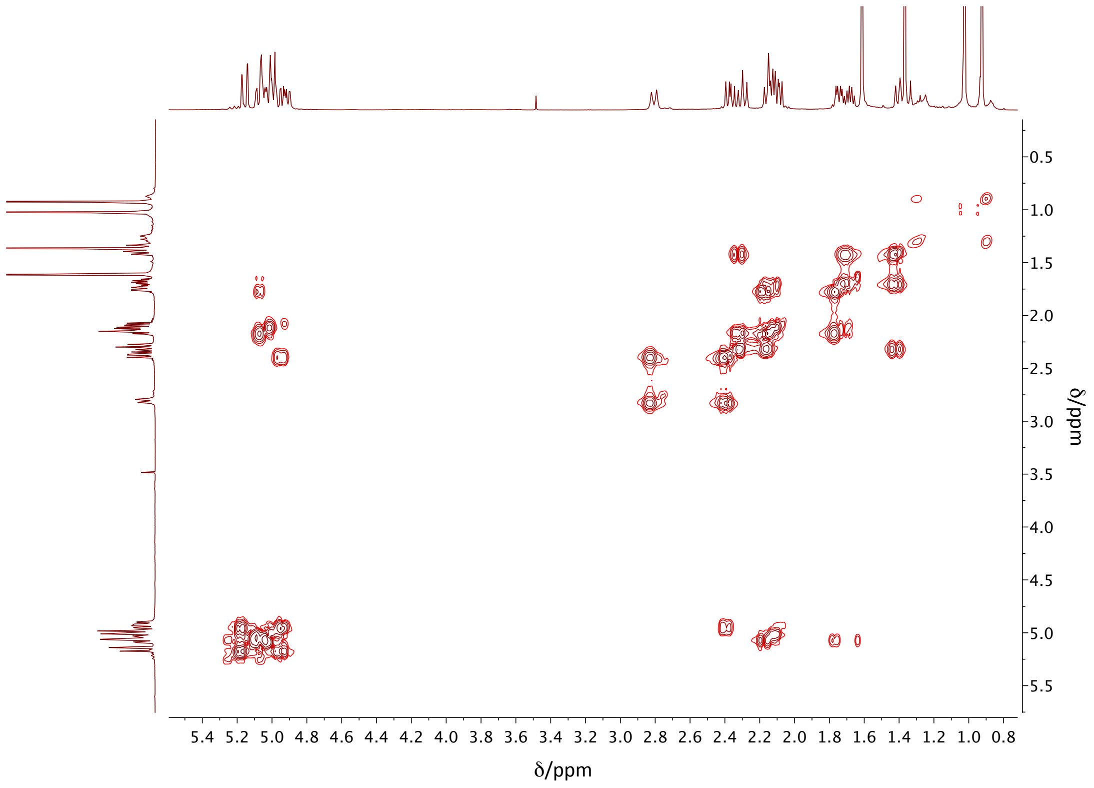

Supplementary Figure 16. $^{1}H,^{1}H$ COSY of compound **10**.

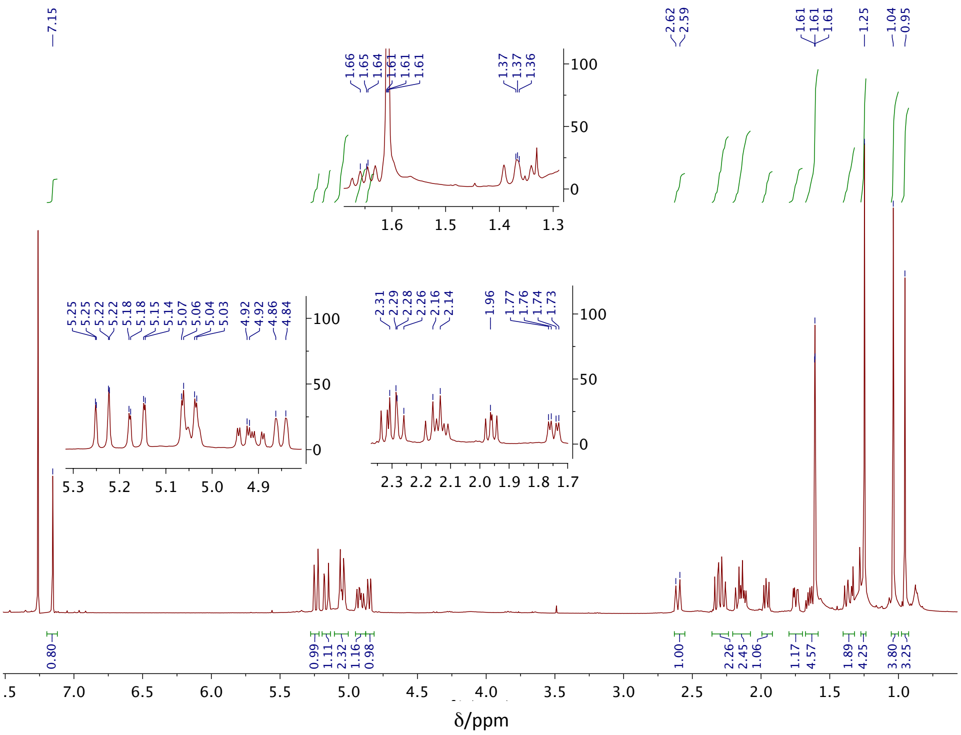

Supplementary Figure 17. ^{1}H NMR of compound **11** in $CDCl_3$ (500 MHz) referenced to $CDCl_3$. See Supplementary Table 3 for assignment.

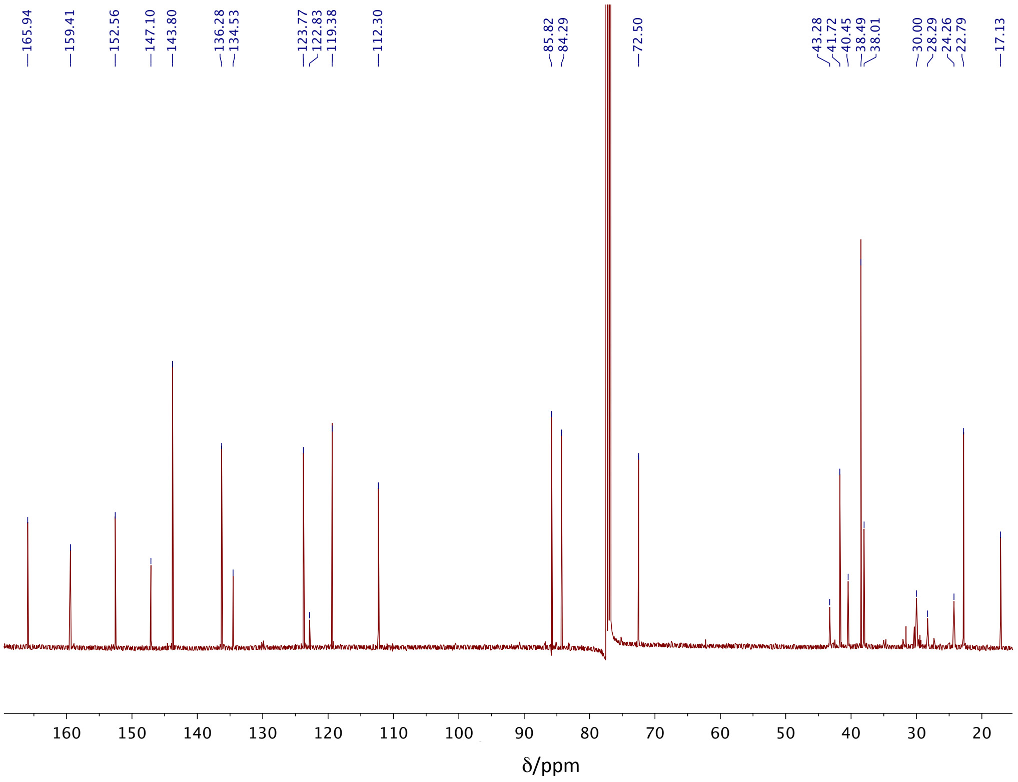

Supplementary Figure 18. ^{13}C NMR of compound **11** in $CDCl_3$ (500 MHz) referenced to $CDCl_3$. See Supplementary Table 3 for assignment.

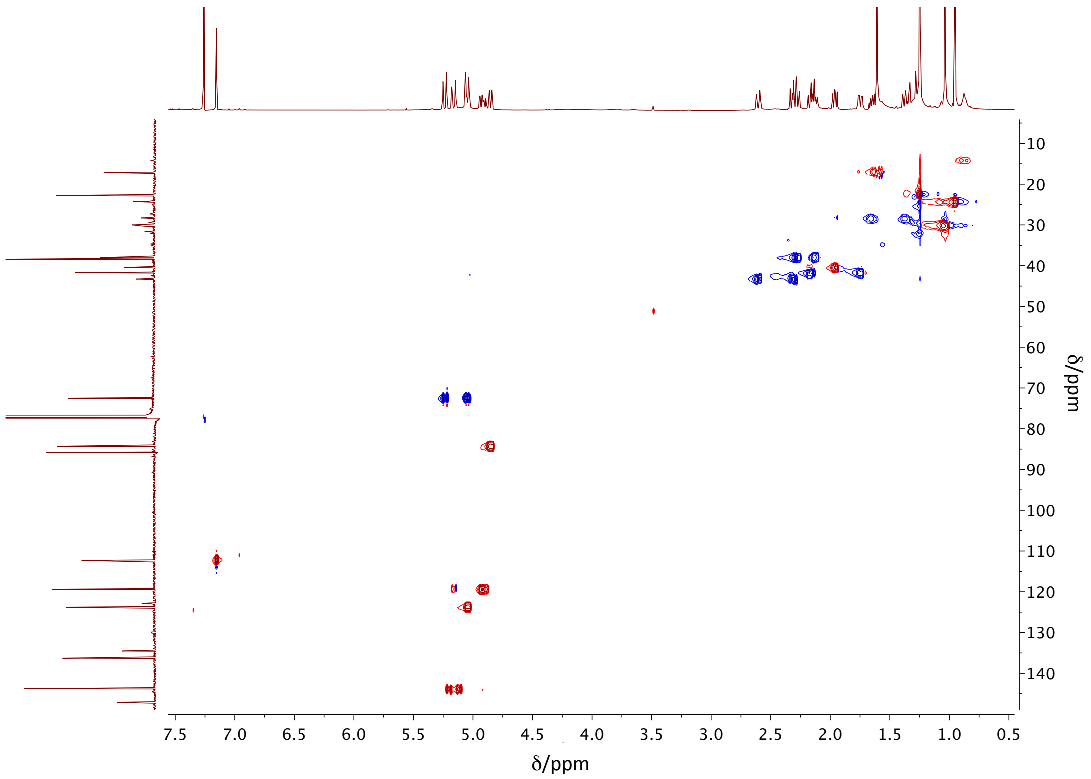

Supplementary Figure 19. HSQC of compound **11**.

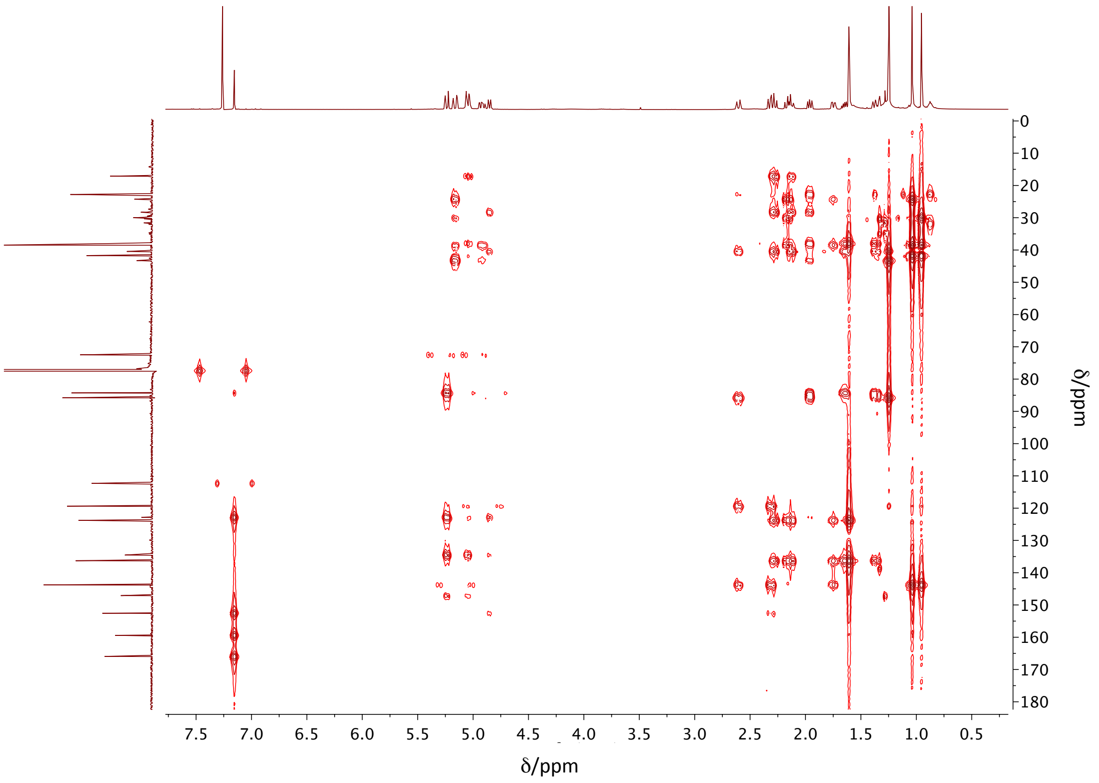

Supplementary Figure 20. HMBC of compound **11**.

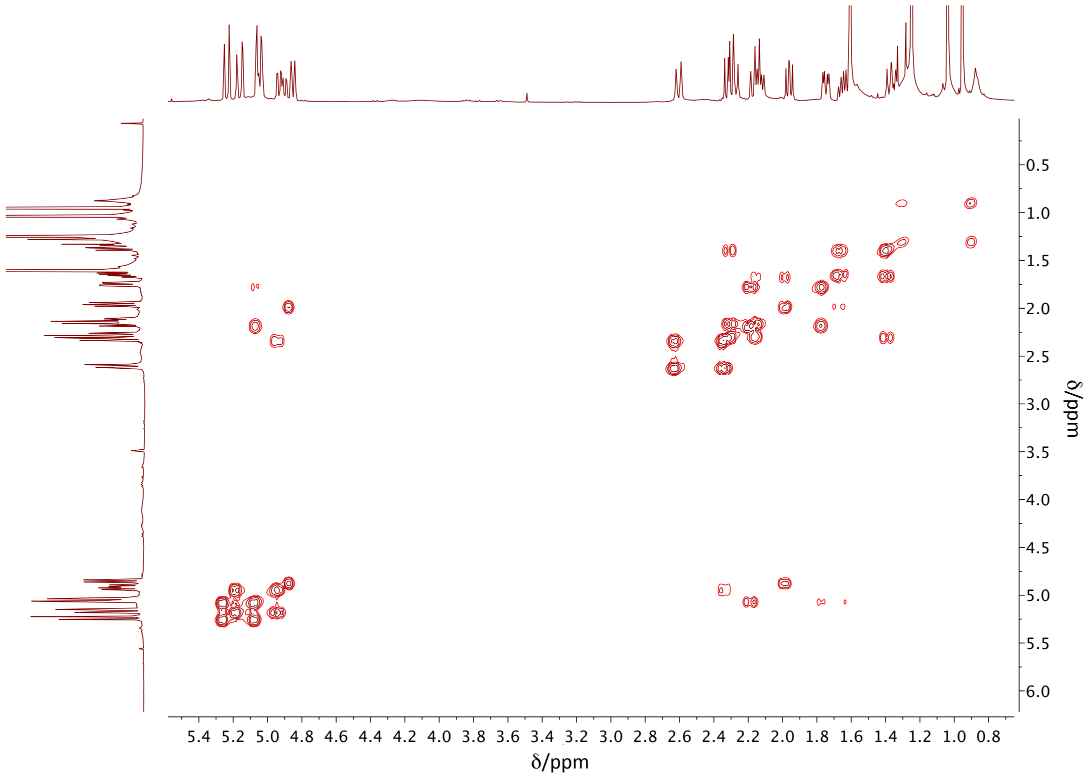

Supplementary Figure 21. ^{1}H,^{1}H COSY of compound **11**.

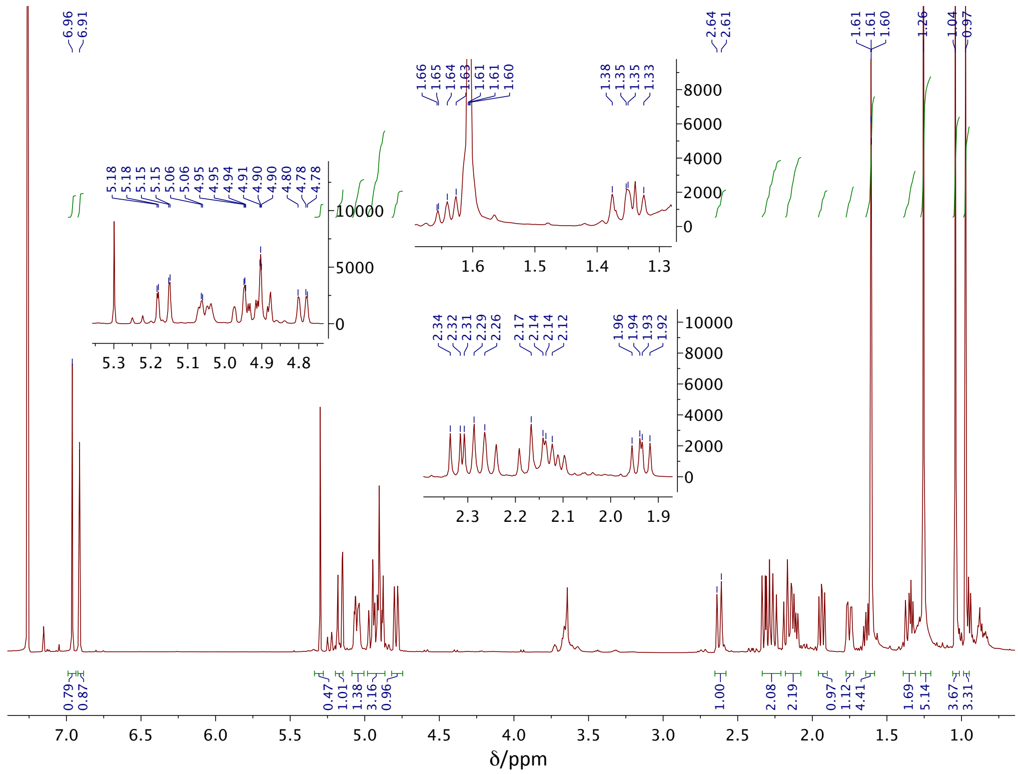

Supplementary Figure 22. ^{1}H NMR of compound **12** in $CDCl_3$ (500 MHz) referenced to $CDCl_3$. See Supplementary Table 4 for assignment.

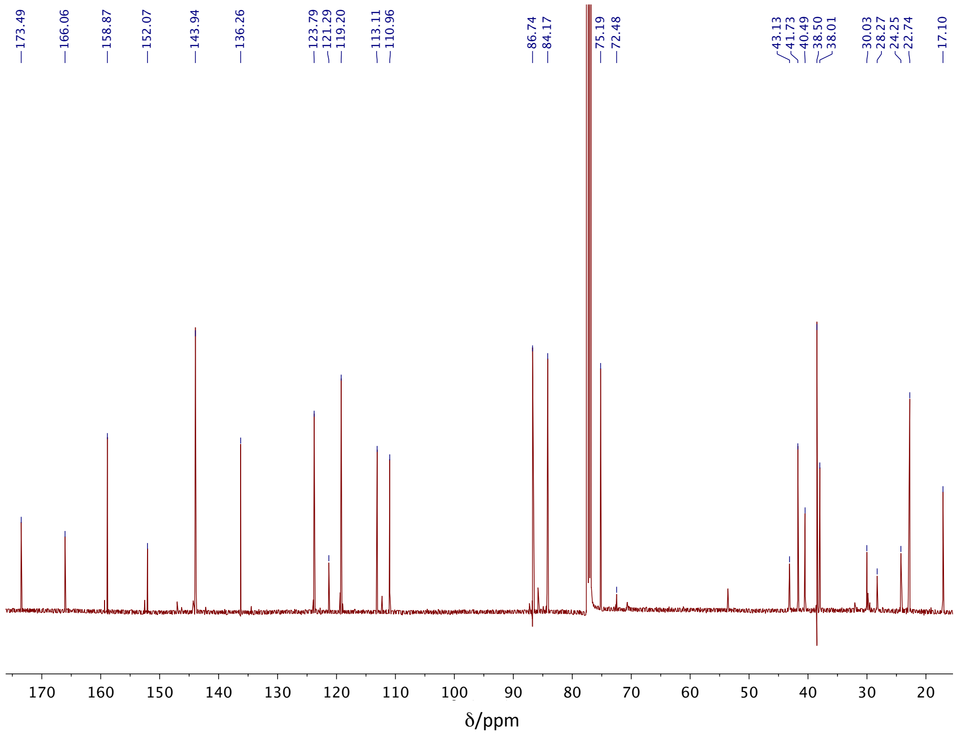

Supplementary Figure 23. ^{13}C NMR of compound **12** in $CDCl_3$ (500 MHz) referenced to $CDCl_3$. See Supplementary Table 4 for assignment.

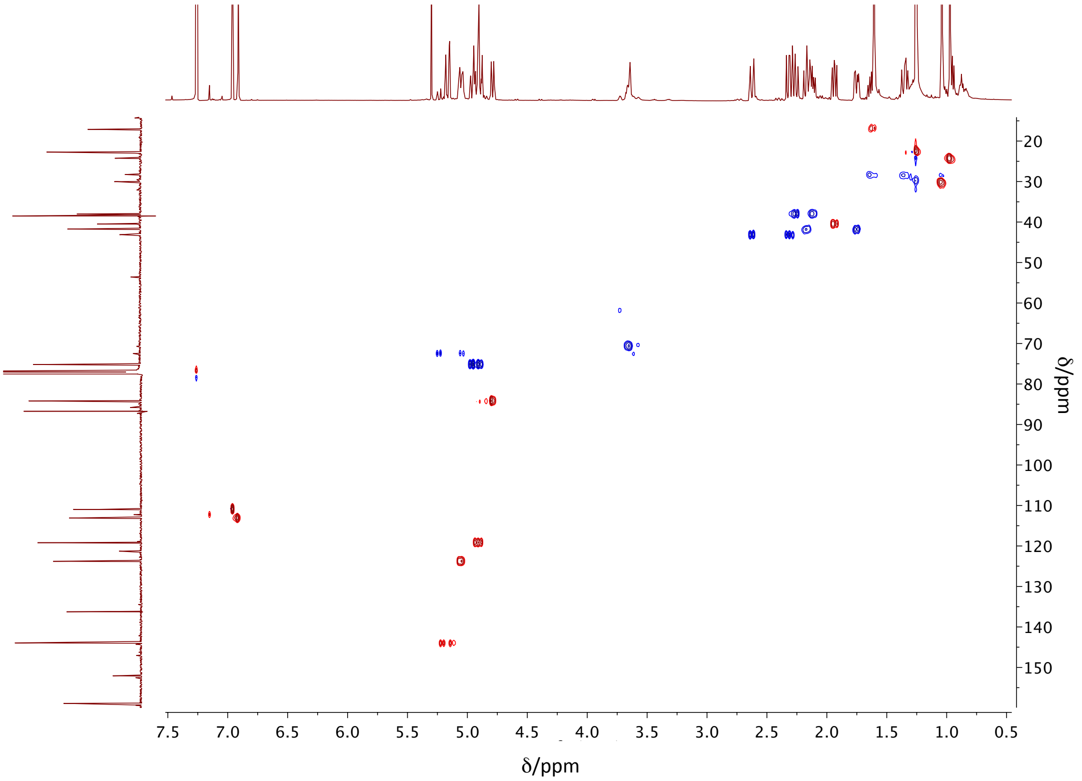

Supplementary Figure 24. HSQC of compound **12**.

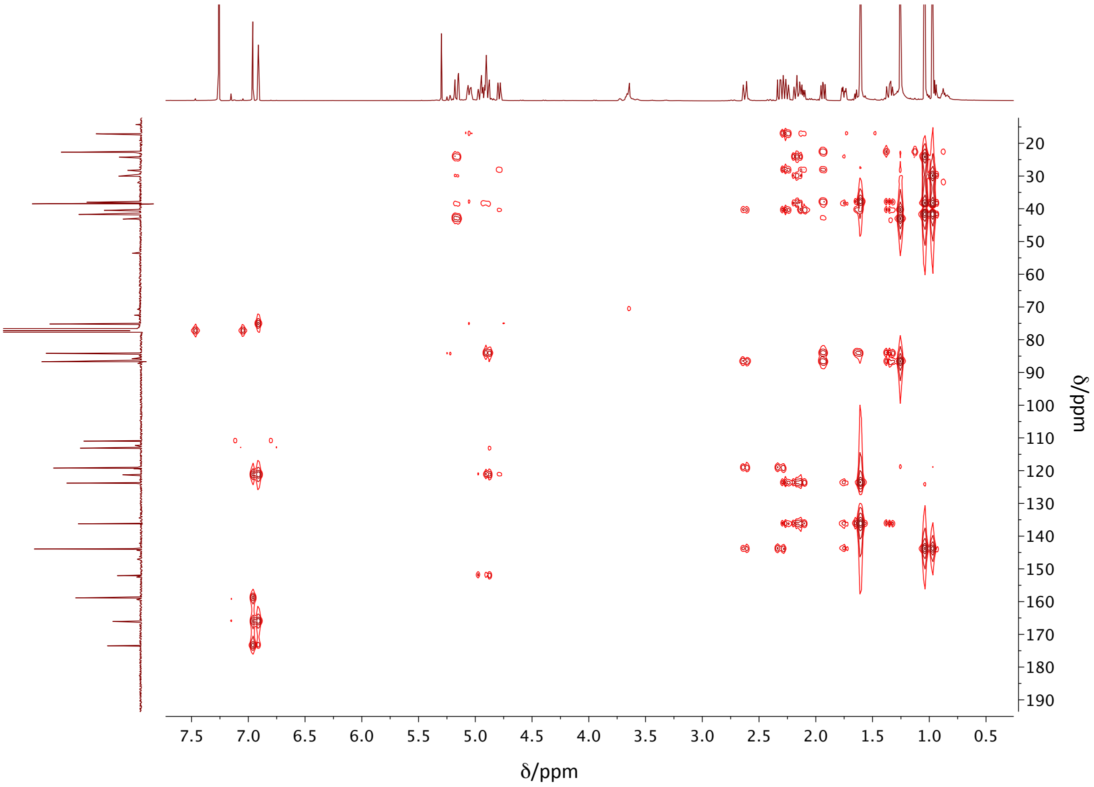

Supplementary Figure 25. HMBC of compound **12**.

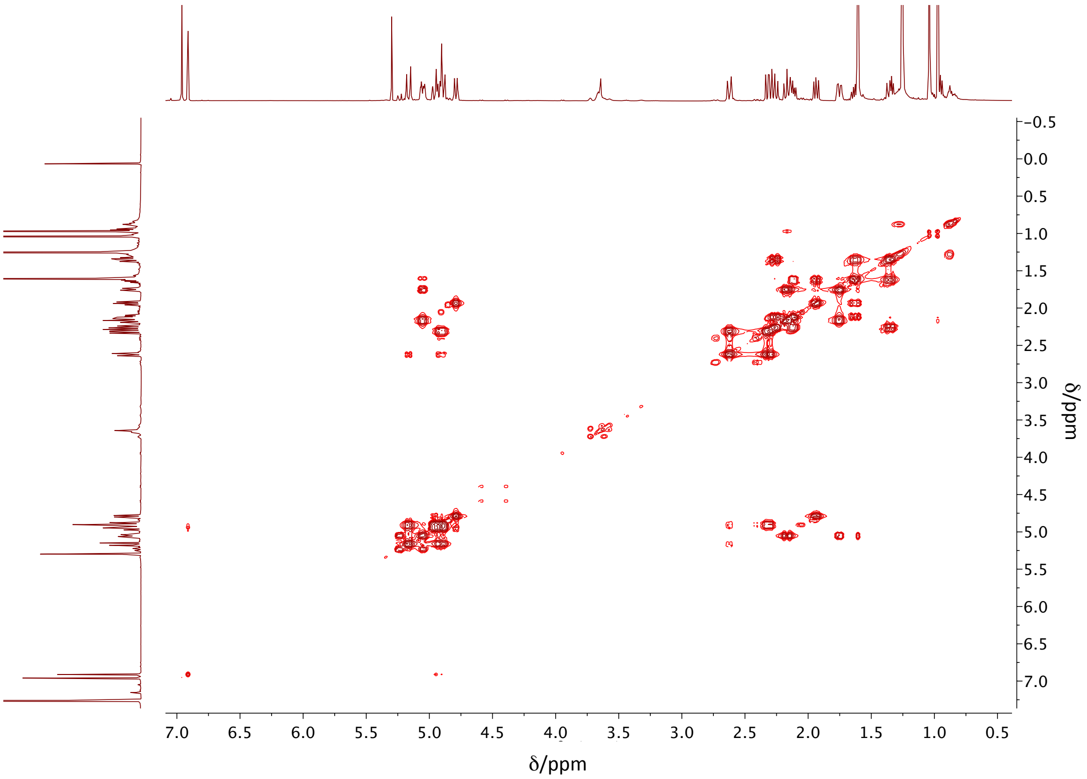

Supplementary Figure 26. $^{1}H,^{1}H$ COSY of compound **12**.

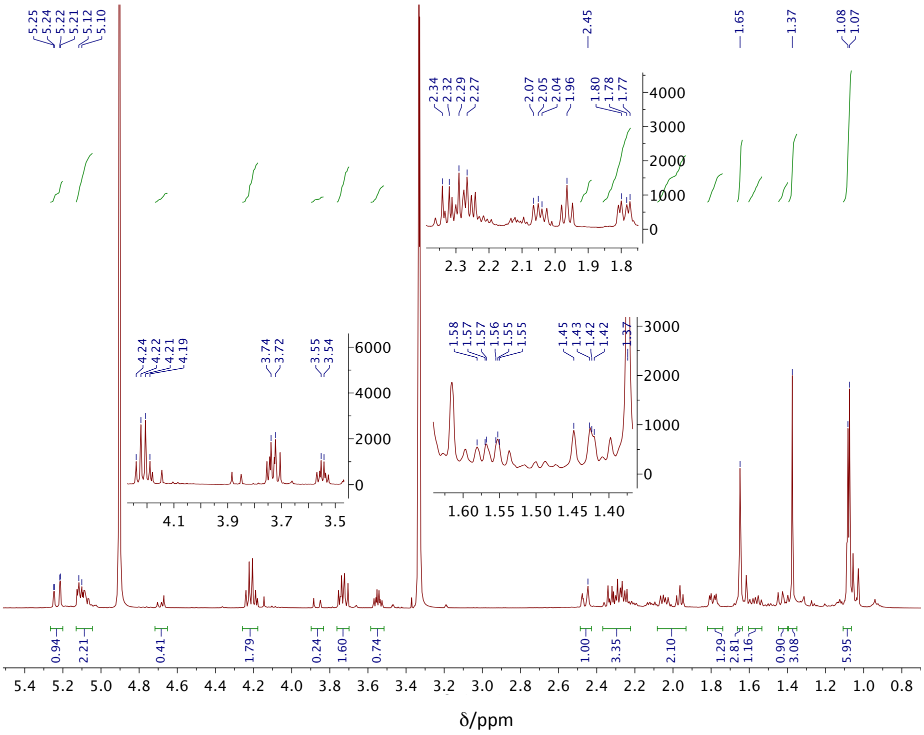

Supplementary Figure 27. ^{1}H NMR of compound **13** in CD_3OD (500 MHz) referenced to CD_3OD. See Supplementary Table 5 for assignment.

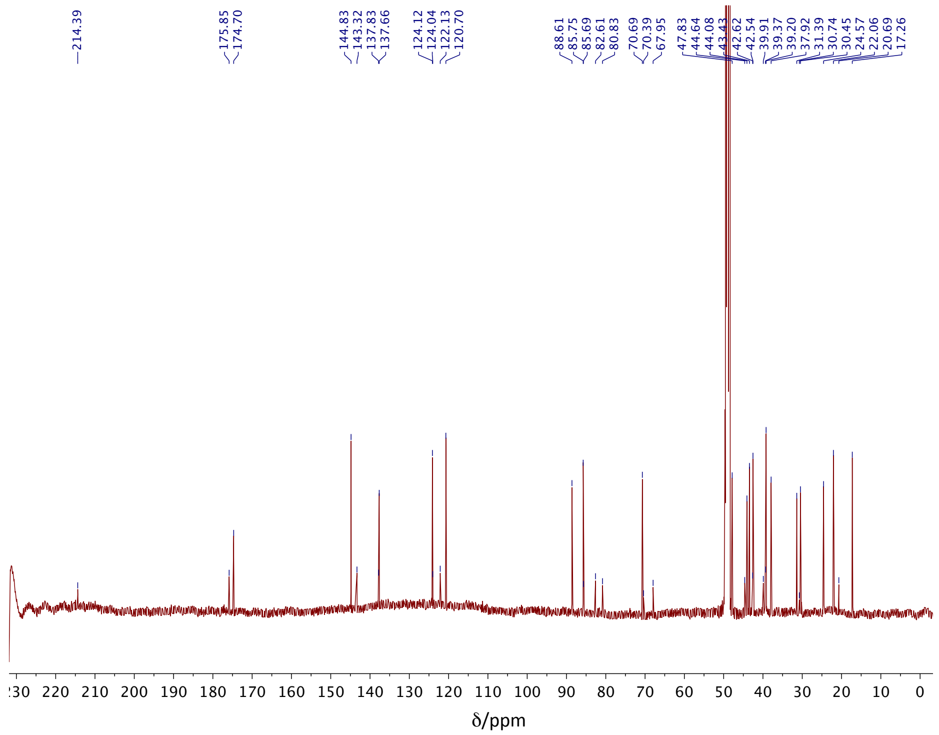

Supplementary Figure 28. ^{13}C NMR of compound **13** in CD_3OD (500 MHz) referenced to CD_3OD. See Supplementary Table 5 for assignment.

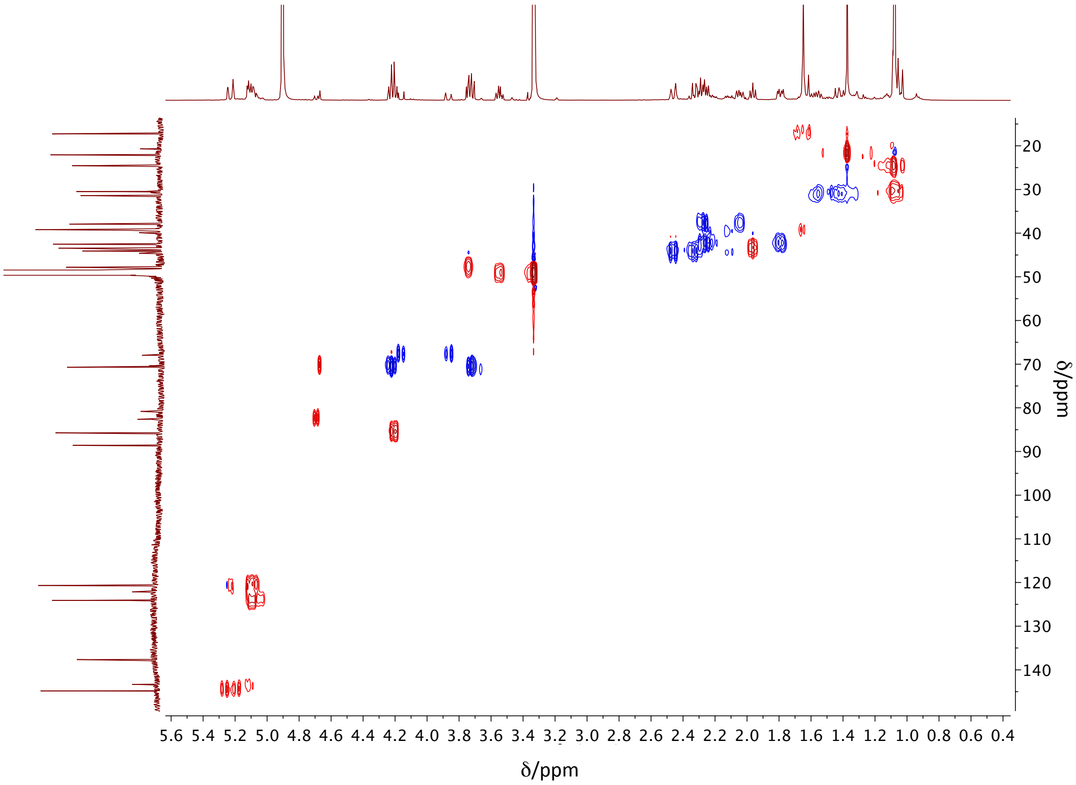

Supplementary Figure 29. HSQC of compound **13**.

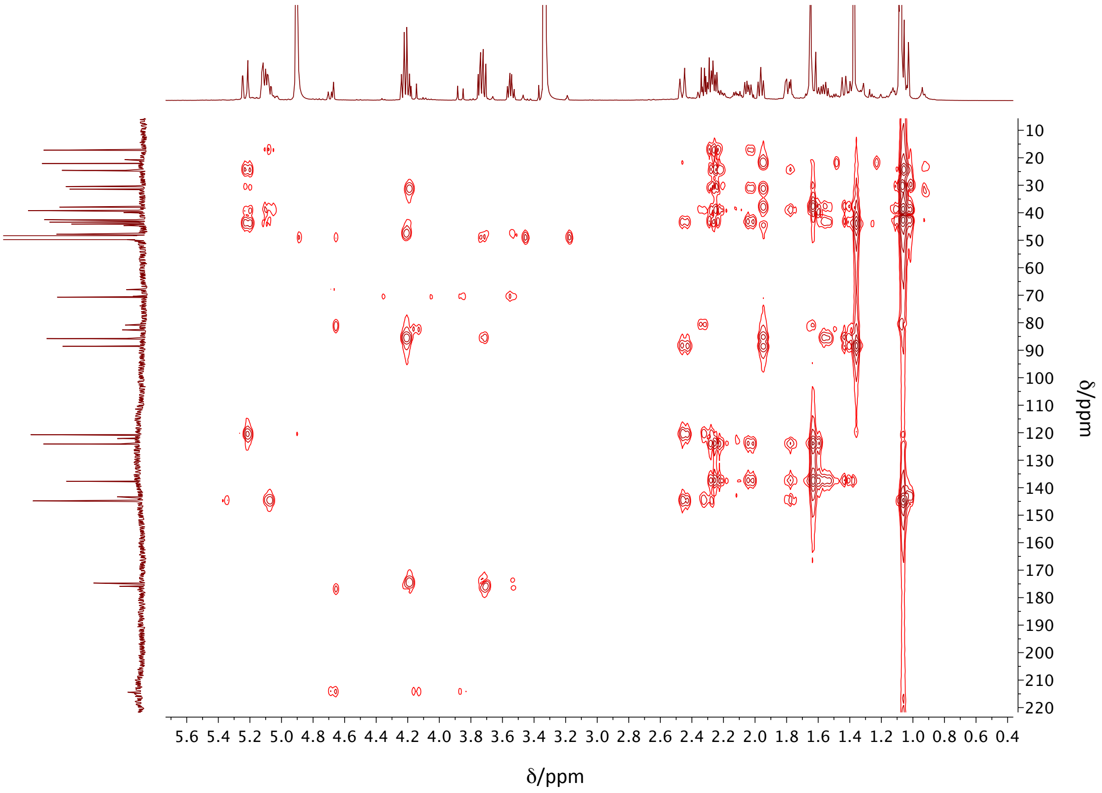

Supplementary Figure 30. HMBC of compound **13**.

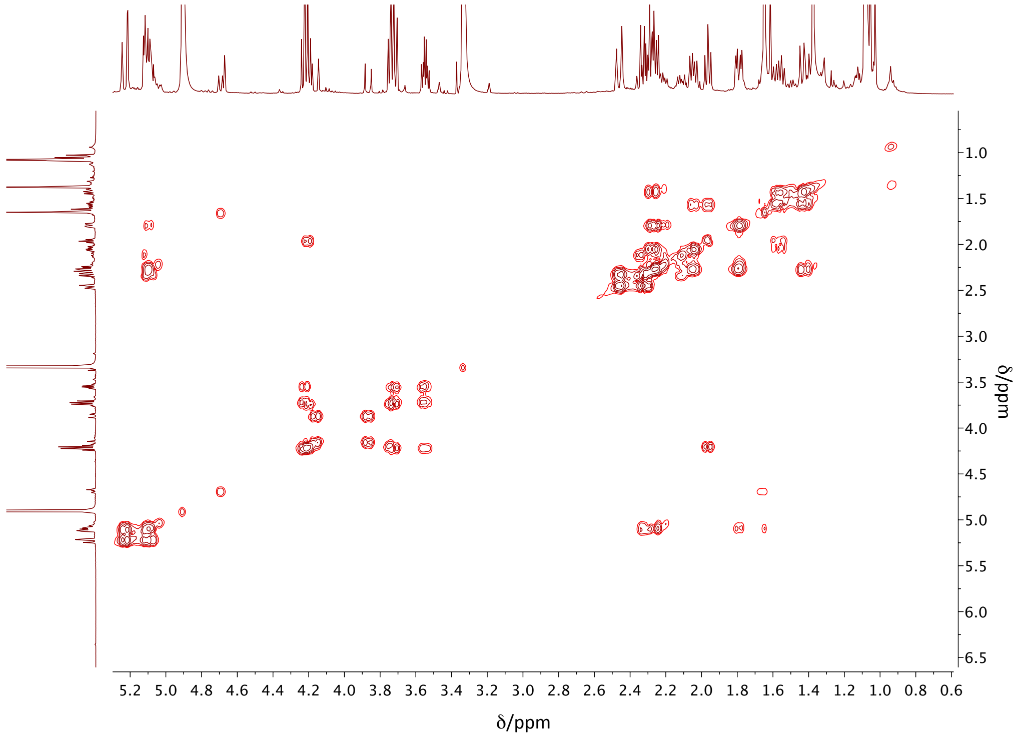

Supplementary Figure 31. ^{1}H,^{1}H COSY of compound **13.**

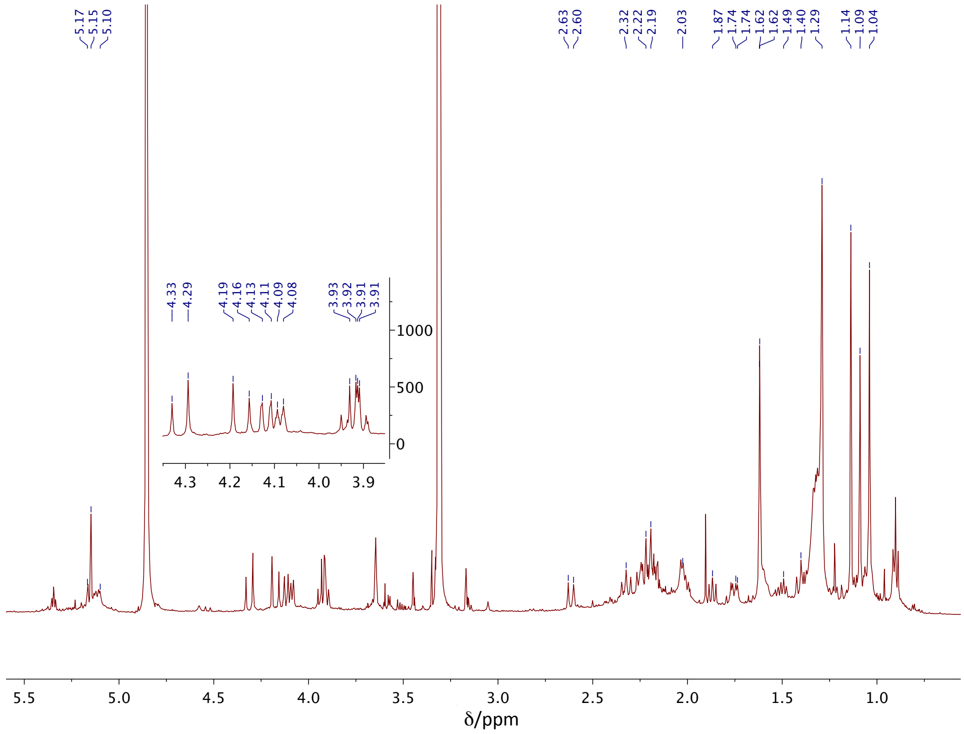

Supplementary Figure 32. ^{1}H NMR of compound **22** in CD_3OD (500 MHz) referenced to CD_3OD. See Supplementary Table 7 for assignment.

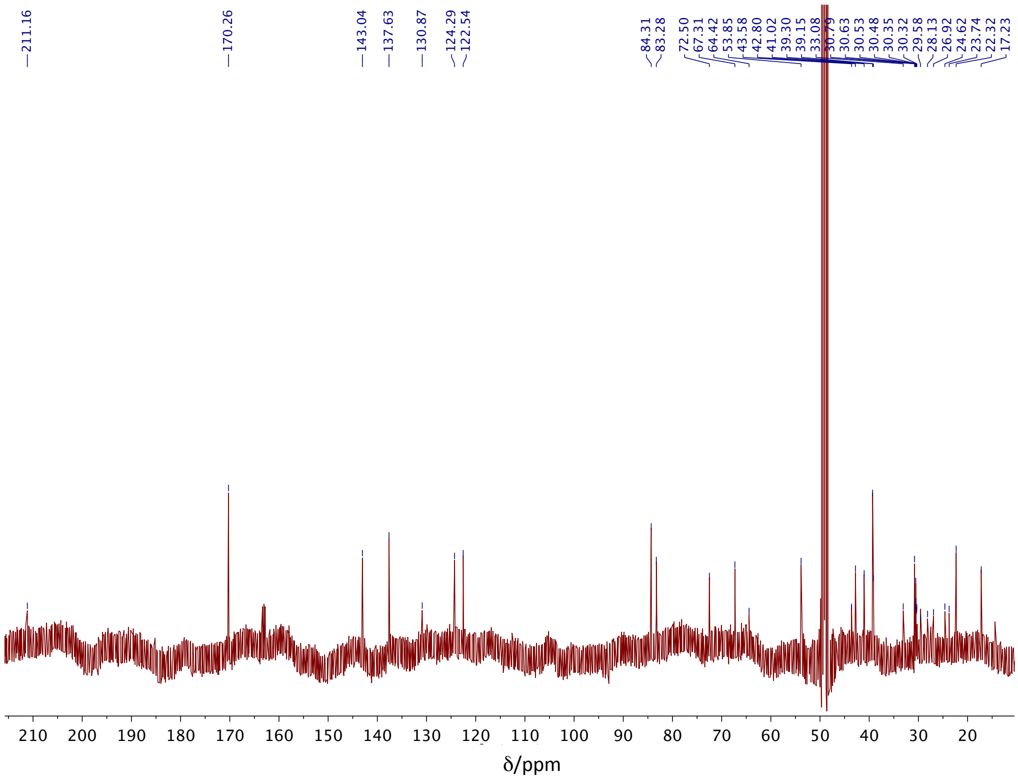

Supplementary Figure 33. ^{13}C NMR of compound **22** in CD_3OD (500 MHz) referenced to CD_3OD. See Supplementary Table 7 for assignment.

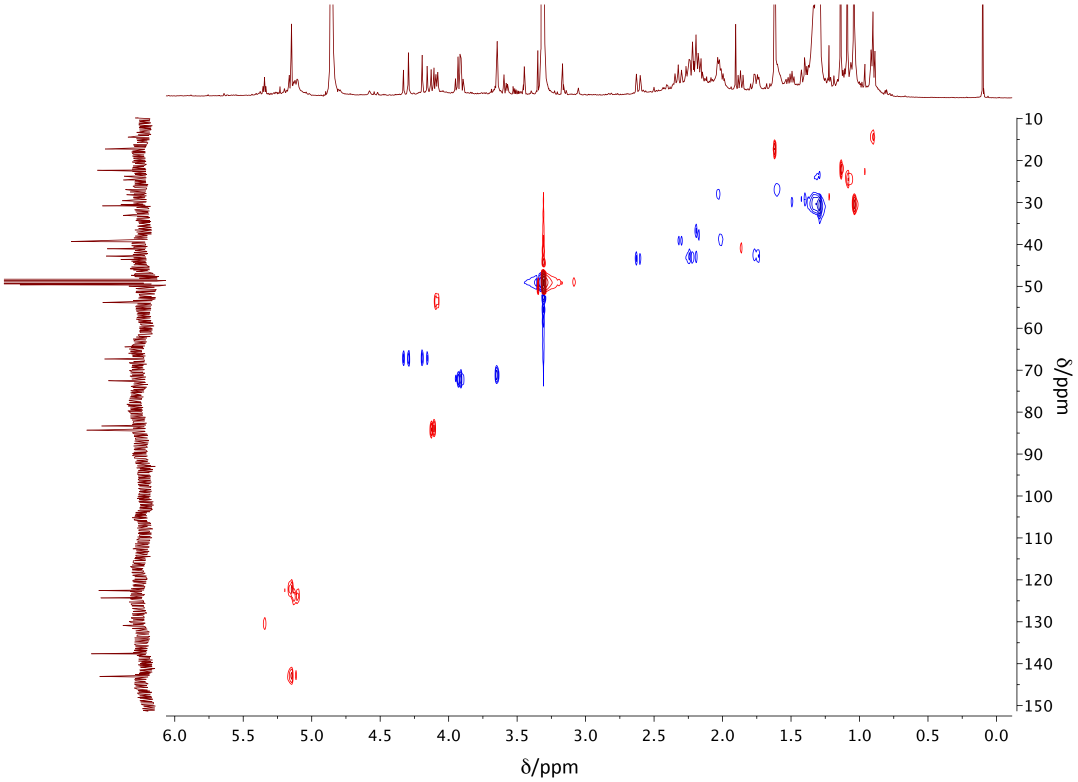

Supplementary Figure 34. HSQC of compound **22**.

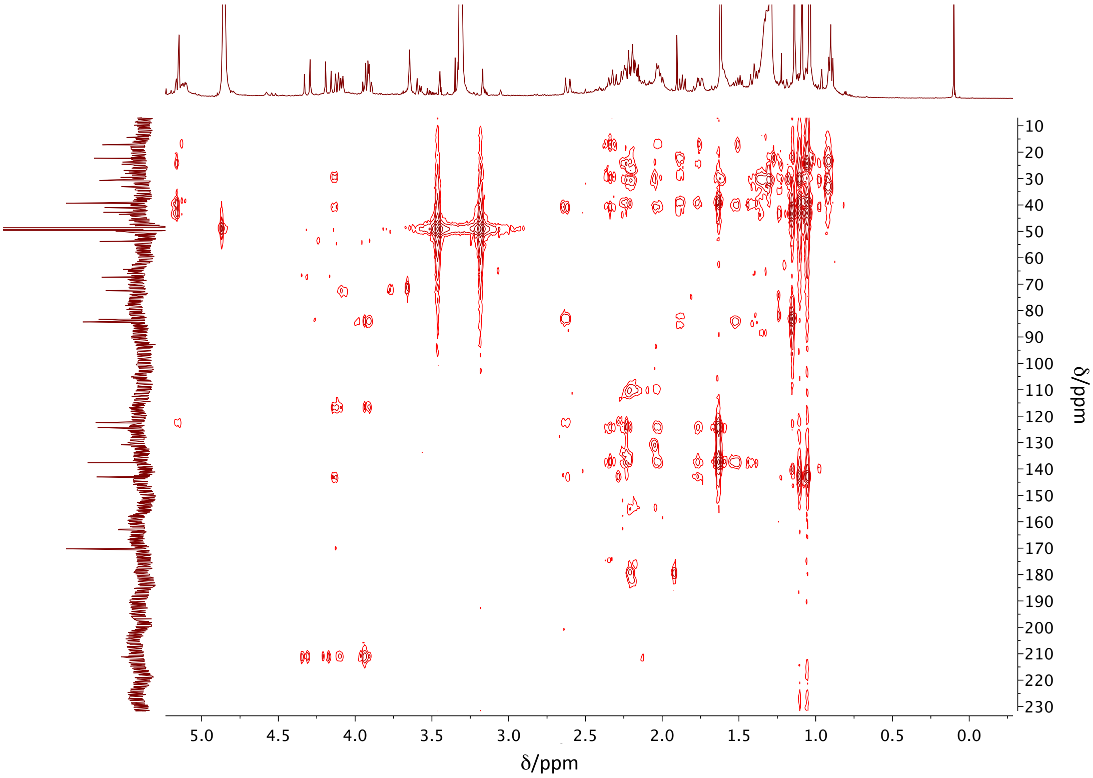

Supplementary Figure 35. HMBC of compound **22**.

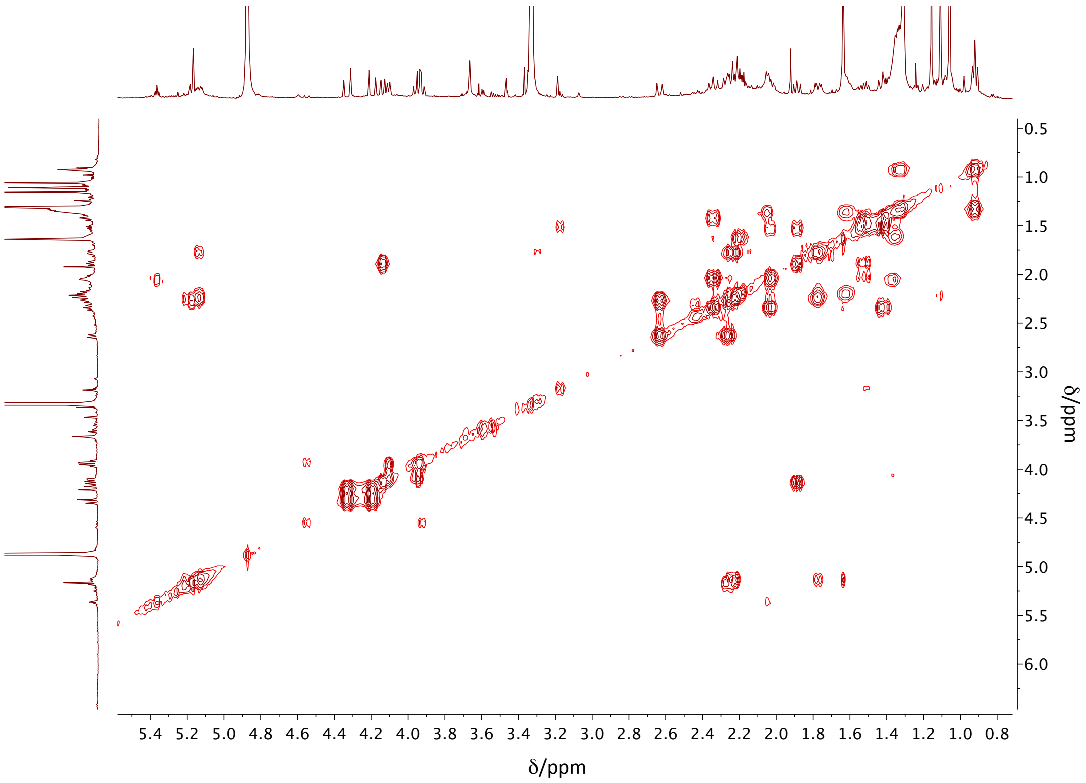

Supplementary Figure 36. ^{1}H,^{1}H COSY of compound **22**.

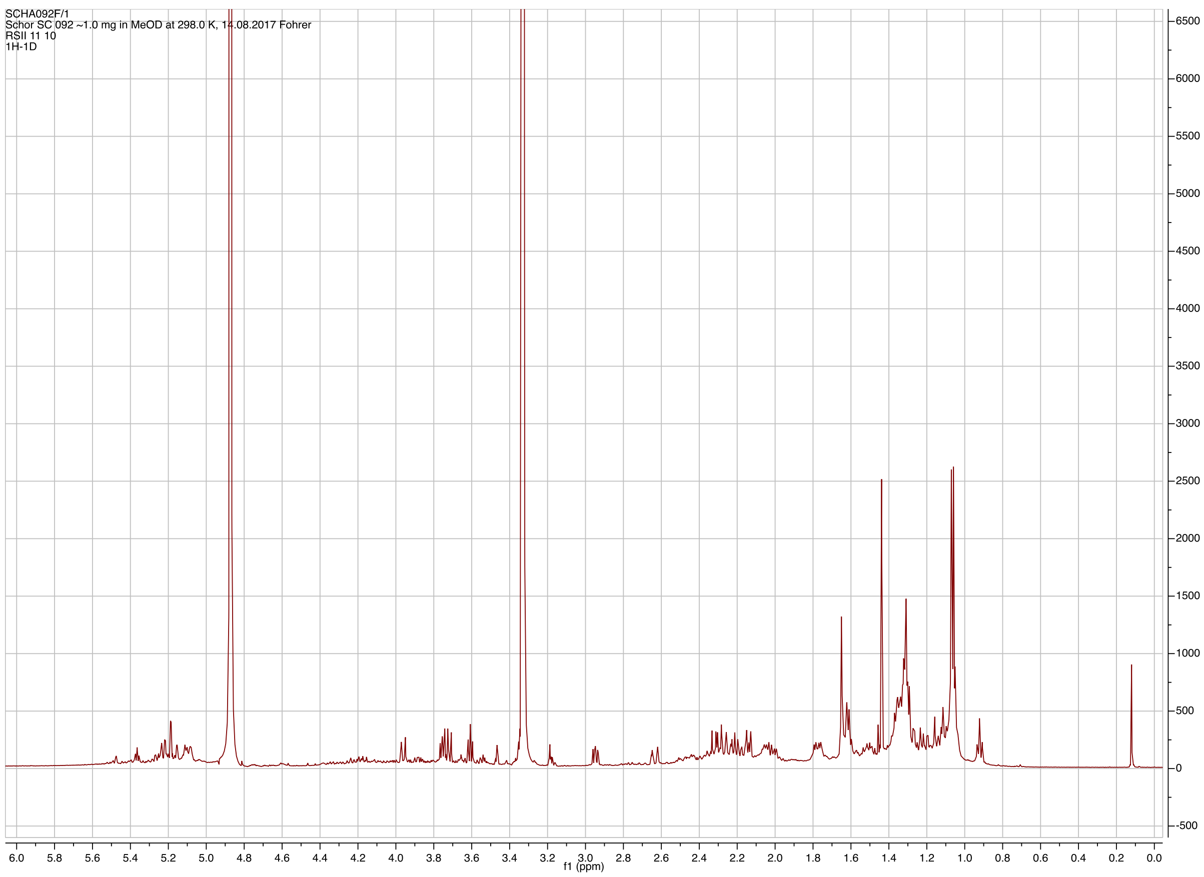

Supplementary Figure 37. ^{1}H NMR of Xenovulene A **1** isolated from *S. schorii.*

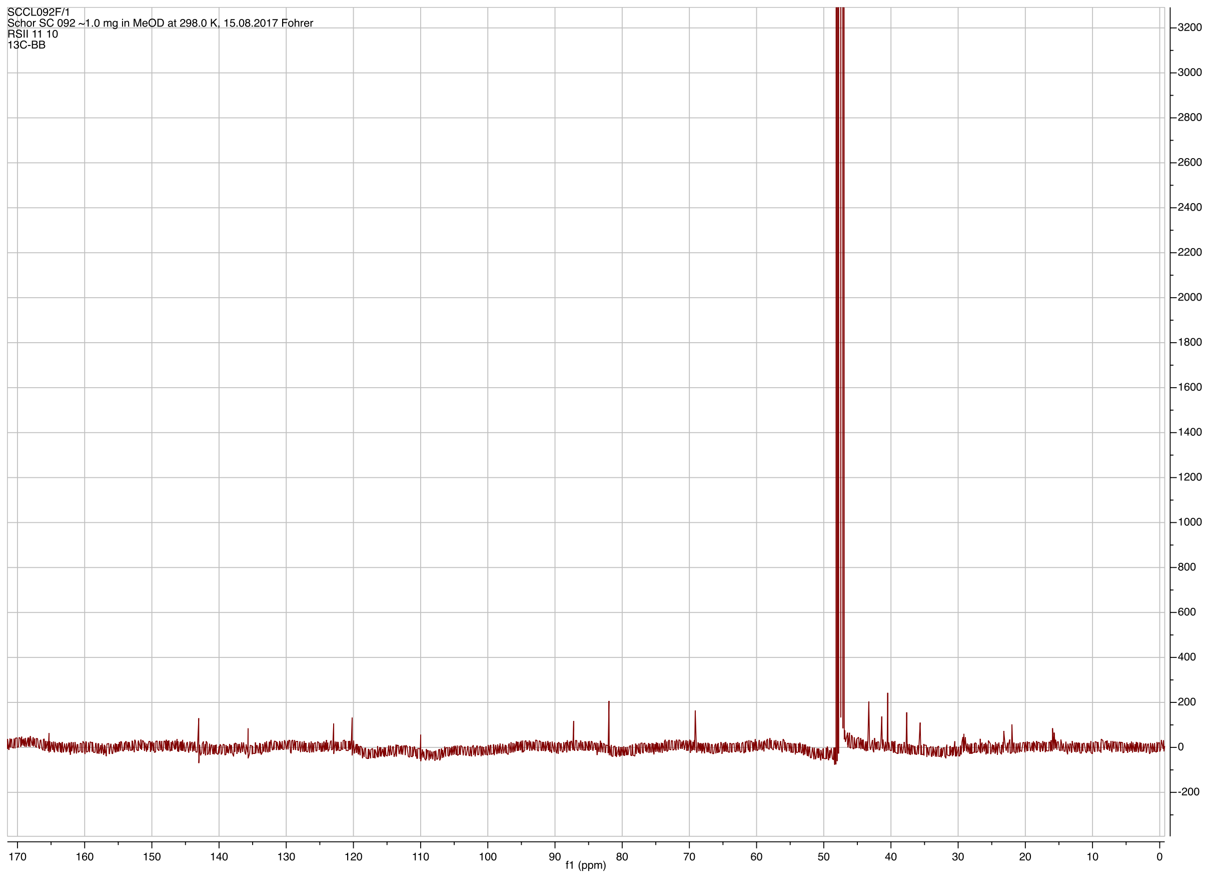

Supplementary Figure 38. ^{13}C NMR of Xenovulene A **1** isolated from *S. schorii.*

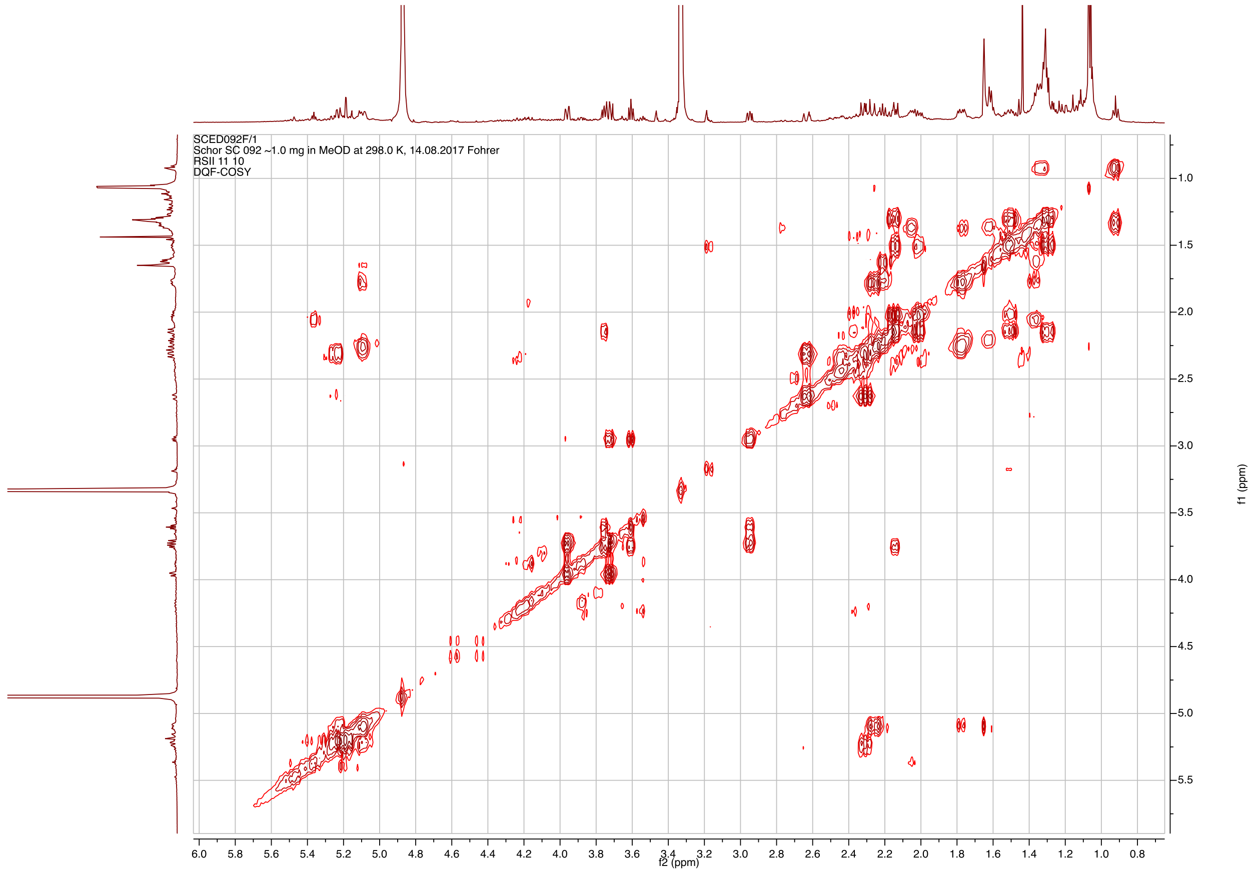

Supplementary Figure 39. ^{1}H, ^{1}H COSY NMR of Xenovulene A **1** isolated from *S. schorii.*

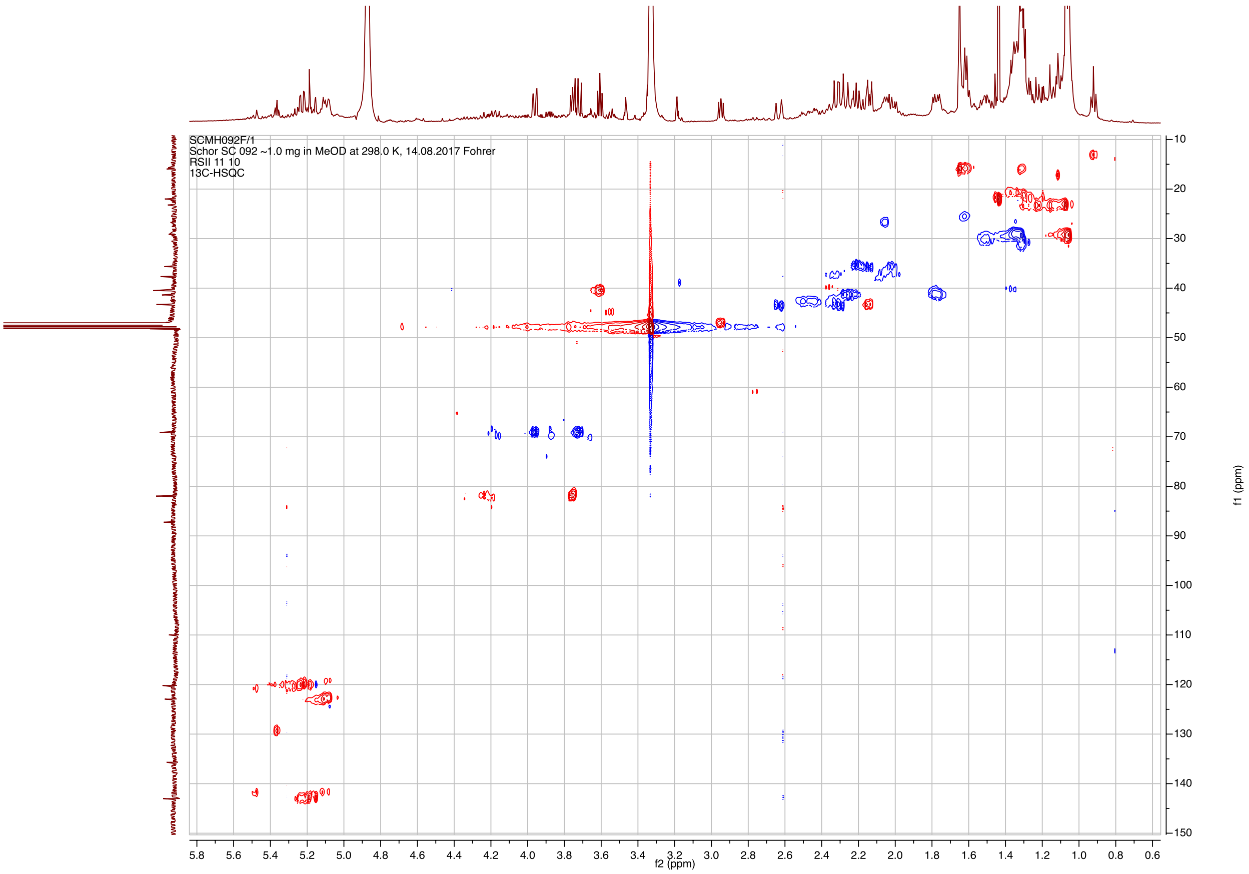

Supplementary Figure 40. HSQC NMR of Xenovulene A **1** isolated from *S. schorii.*

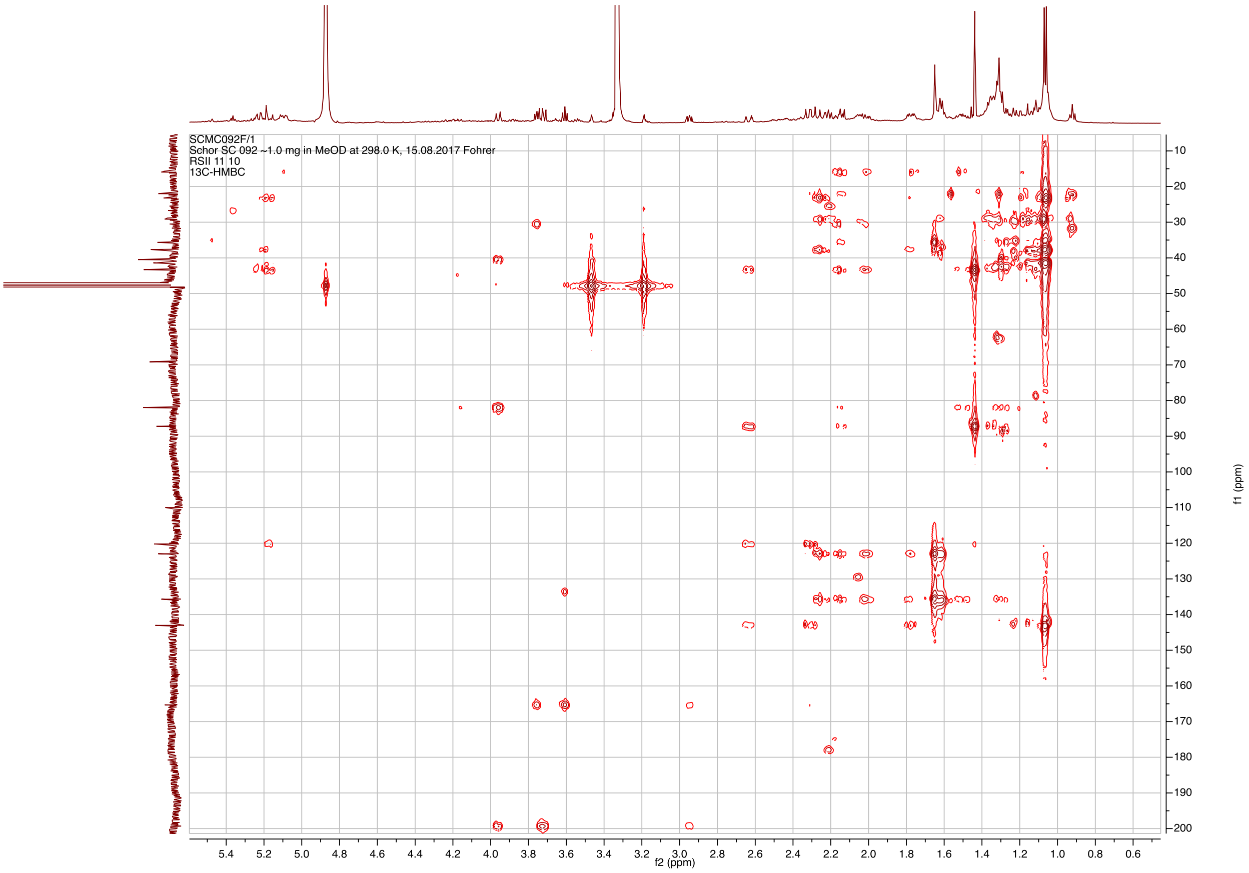

Supplementary Figure 41. HMBC NMR of Xenovulene A **1** isolated from *S. schorii.*

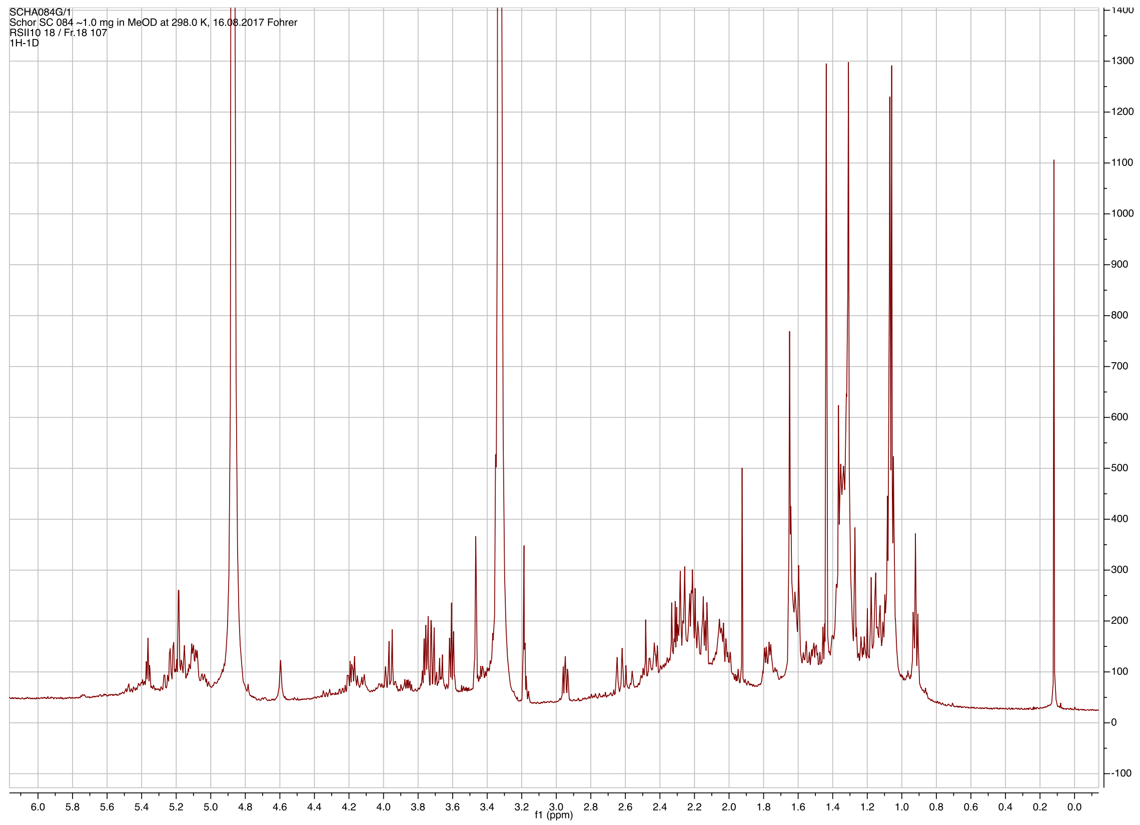

Supplementary Figure 42. ^{1}H NMR of Xenovulene A **1** Isolated from *A. oryzae*.

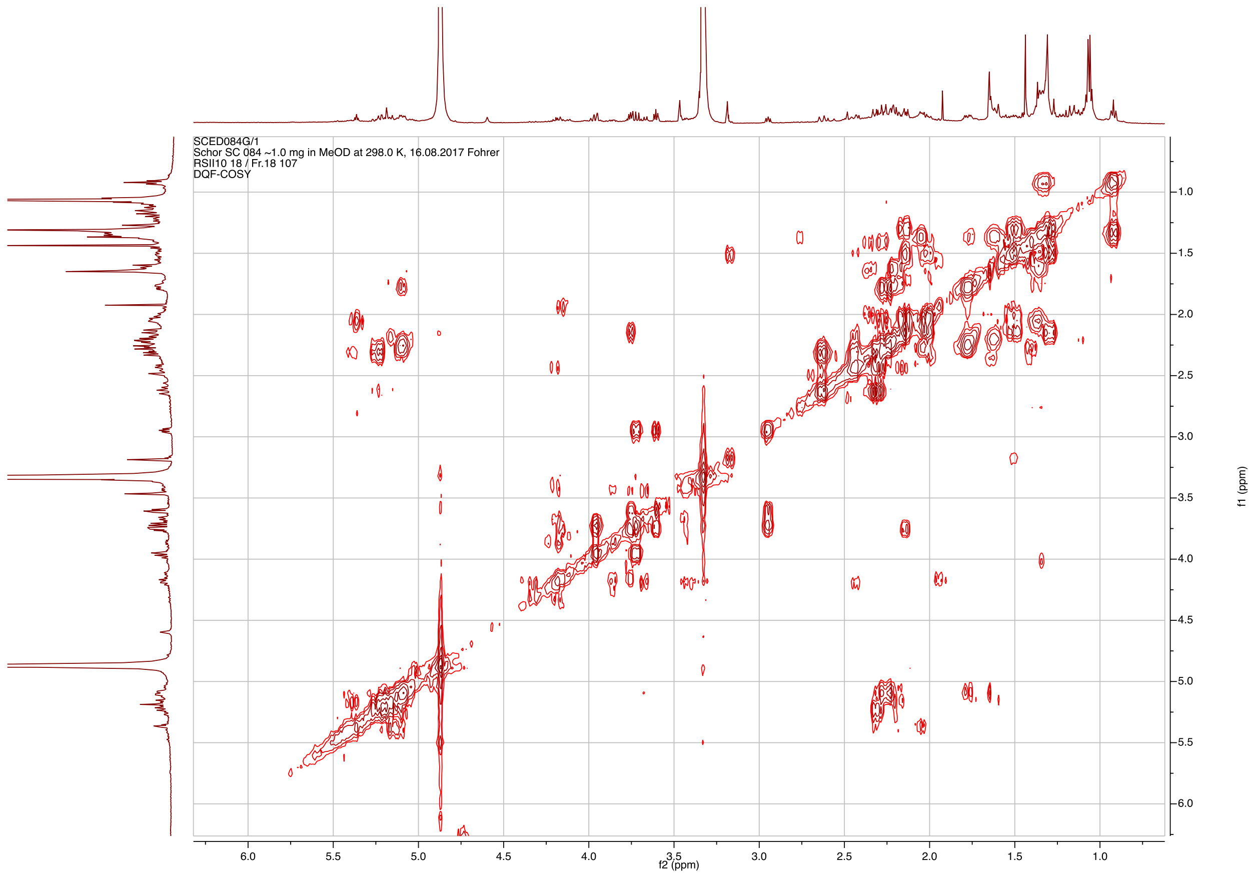

Supplementary Figure 43. ^{1}H, ^{1}H COSY NMR of Xenovulene A **1** Isolated from *A. oryzae*.

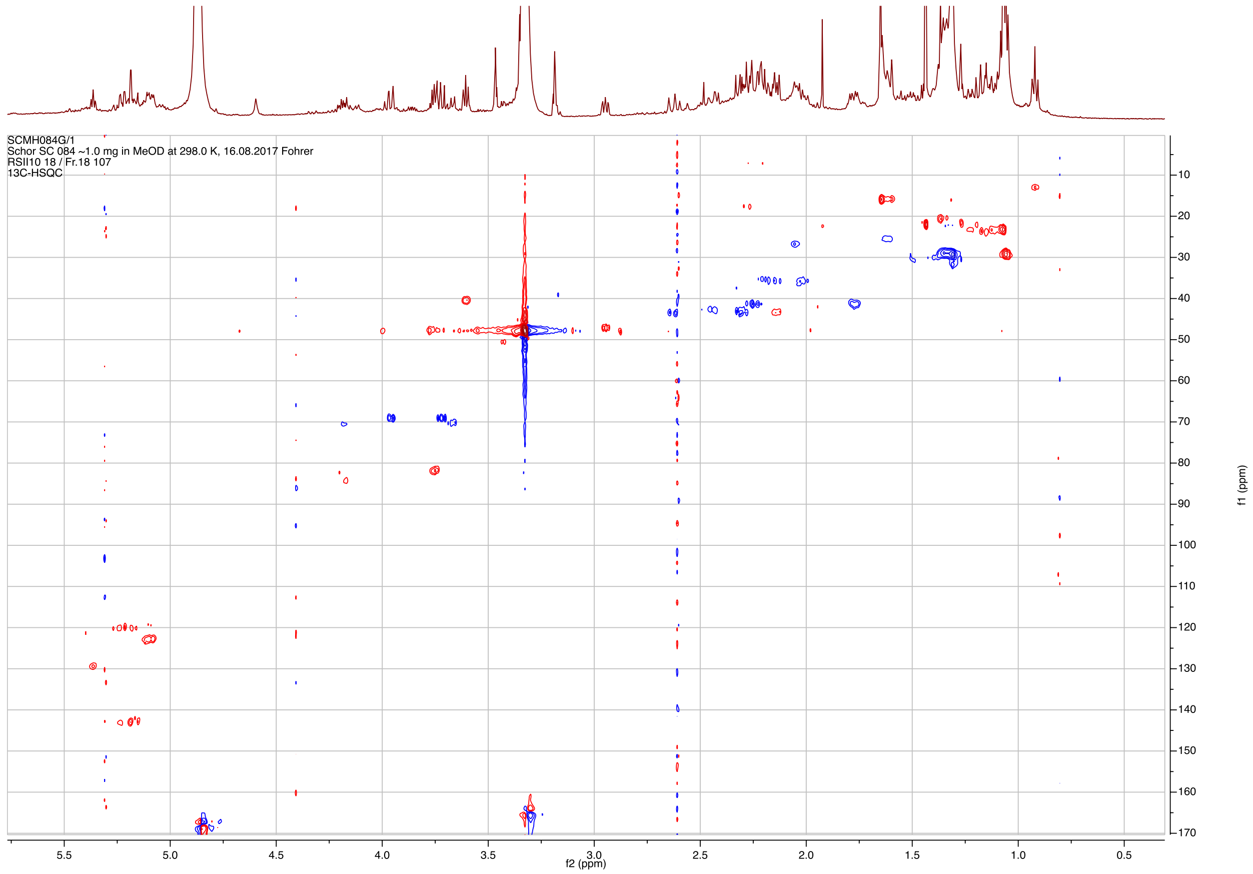

Supplementary Figure 44. HSQC NMR of Xenovulene A **1** Isolated from *A. oryzae*.

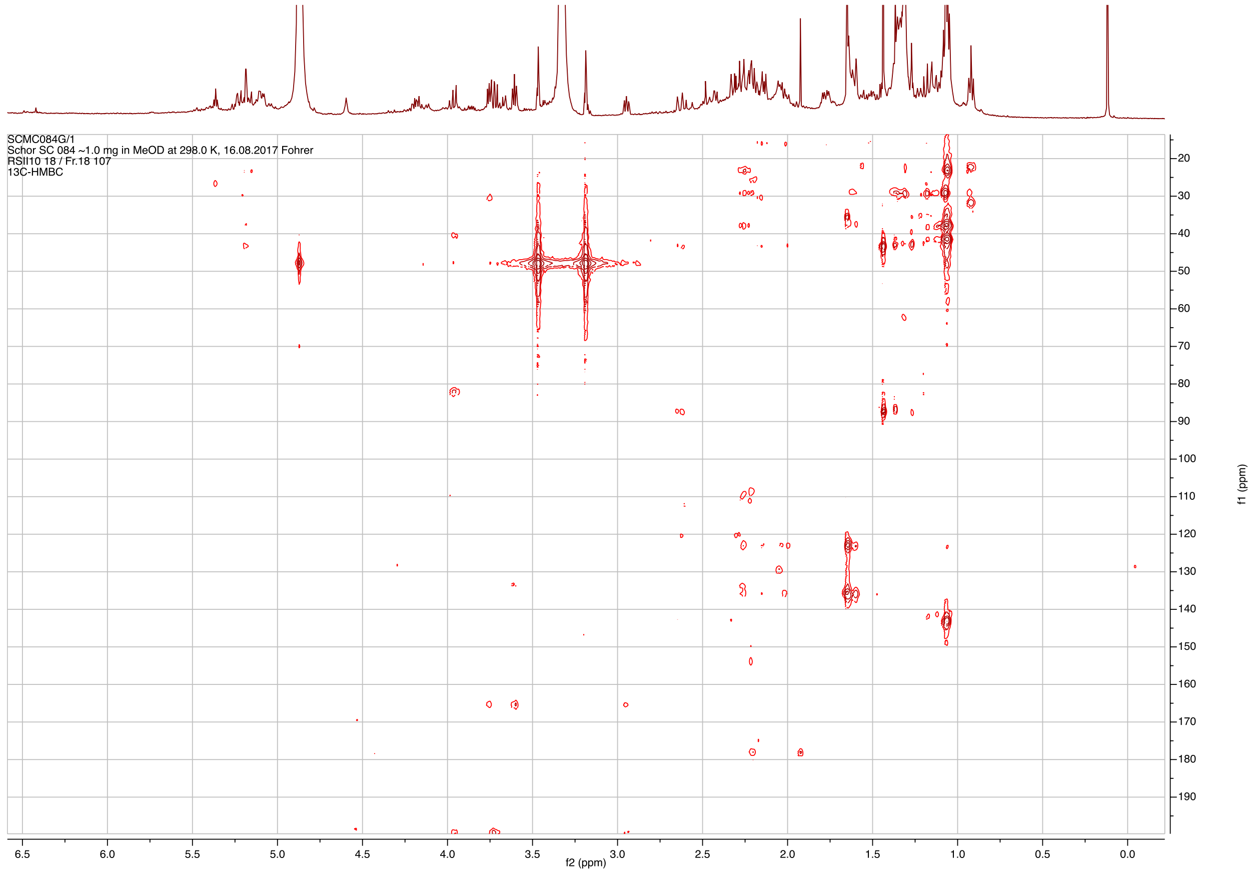

Supplementary Figure 45. HMBC NMR of Xenovulene A **1** Isolated from *A. oryzae*.

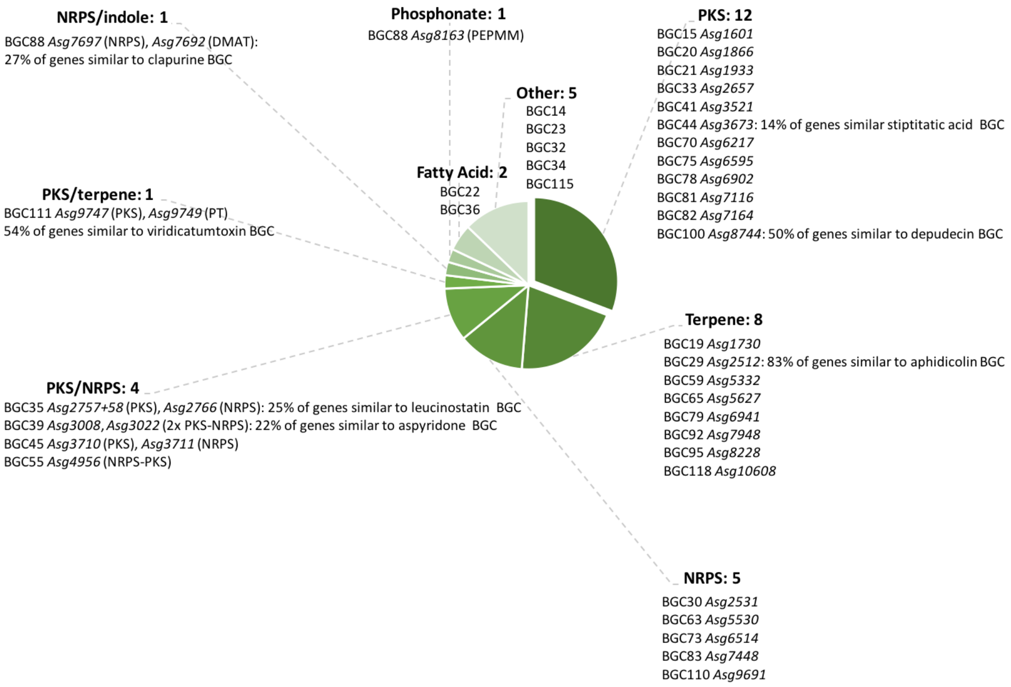

Supplementary Figure 46. Pie chart of BGC (Biosynthetic Gene Cluster) predicted by fungiSMASH with a match to PKS, NRPS or terpene gene. BGC numbers are denoted as well as the core gene (encoding PKS [Polyketide synthase], NRPS [Non-ribosomal peptide synthetase] and terpene) is listed with gene number. Structures of natural products are shown for BGC, which show similarity to characterised BGCs.

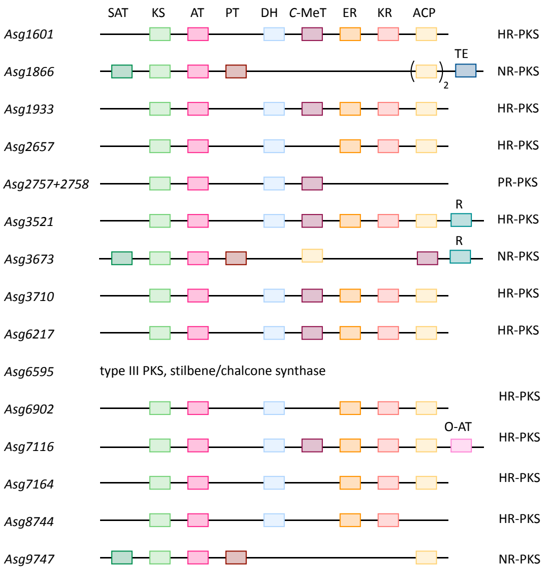

Supplementary Figure 47. Conserved domain analysis of PKS (Polyketide synthase) genes in the *A. strictum* genome. SAT = starter unit acyl transferase, KS = ketosynthase, AT = acyltransferase, PT = product template, DH = dehydratase, *C*-Met = *C*-methyl transferase, ER = enoylreductase, KR = ketoreductase, ACP = acyl carrier protein, TE = thiolesterase, R = reductive release, *O*-AT = *O*-acyltransferase.

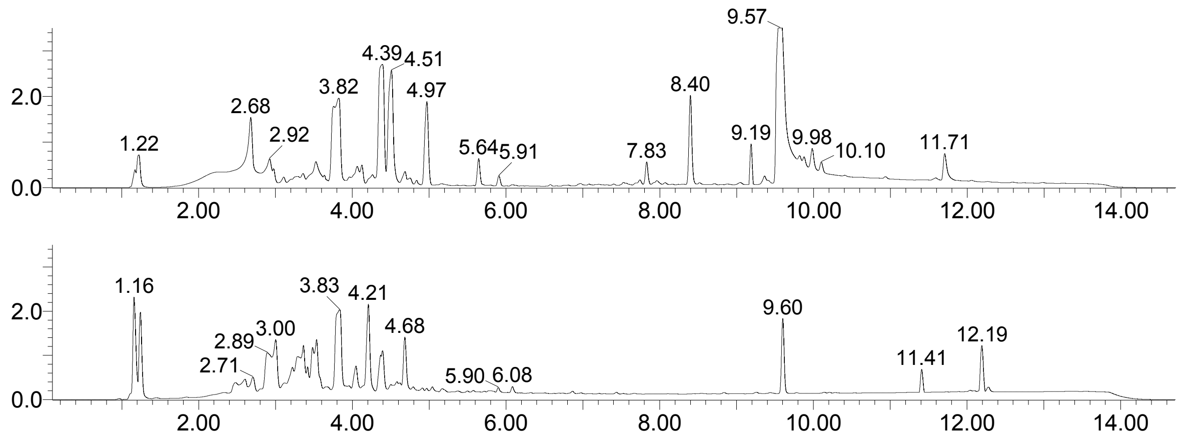

Supplementary Figure 48. DAD (Diode Array Detector) chromatograms of *A. schorii* extracts grown under producing (upper) and non-producing (lower) conditions. Peaks at t_R = 8.4, 9.2, 9.6, 10.0 and 11.7 correspond to xenovulenes **1**, **10**, **11**, **9** and **7** respectively.

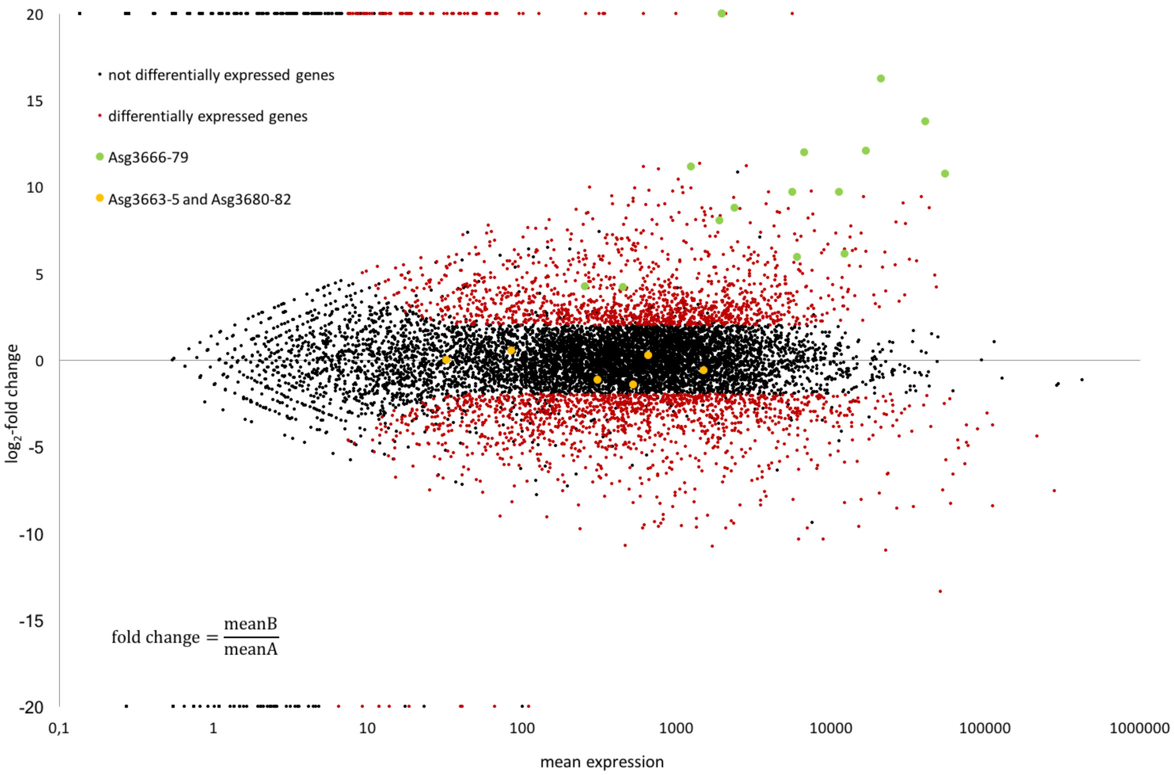

Supplementary Figure 49. Scatter diagram shows mean expression under both conditions (mean(A+B)/2) over $\log_2$-fold change. Every dot correlates to one gene, green dots represent Asg3666-79 and orange dots represent Asg3663-5 + Asg3680-82.

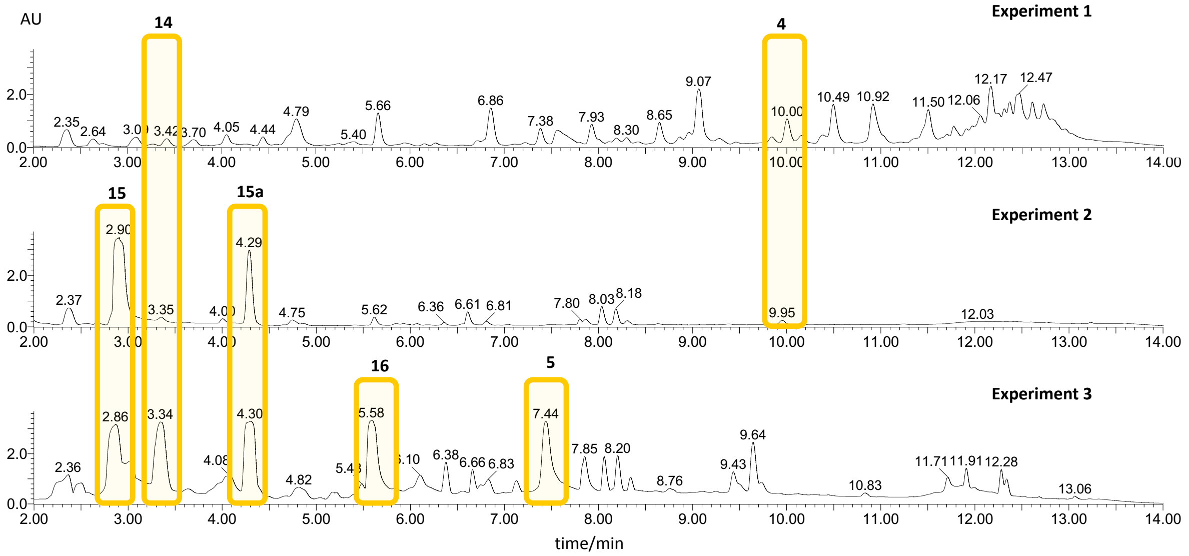

Supplementary Figure 50. DAD (Diode Array Detector) chromatogram of *A. oryzae* NSAR1 extracts measured with LCMS (Liquid Chromatography Mass Spectrometry) method 2. Extracts were obtained from transformants (DPY, 4 d) expressing different set of genes as indicated in Table 2 experiments 1-3 (Main Text).

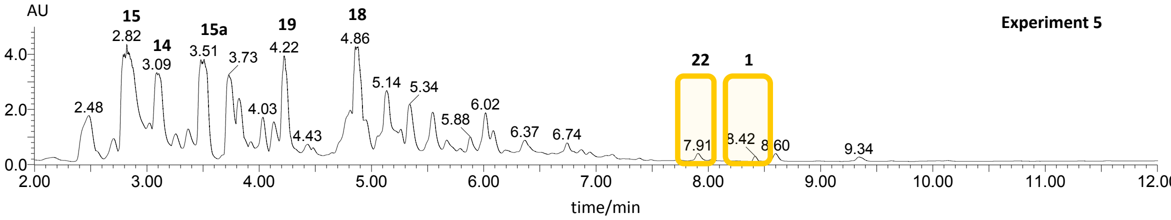

Supplementary Figure 51. DAD (Diode Array Detector) chromatogram of *A. oryzae* NSAR1 extracts measured with LCMS (Liquid Chromatography Mass Spectrometry) method 1 for experiment 5 (Main Text).

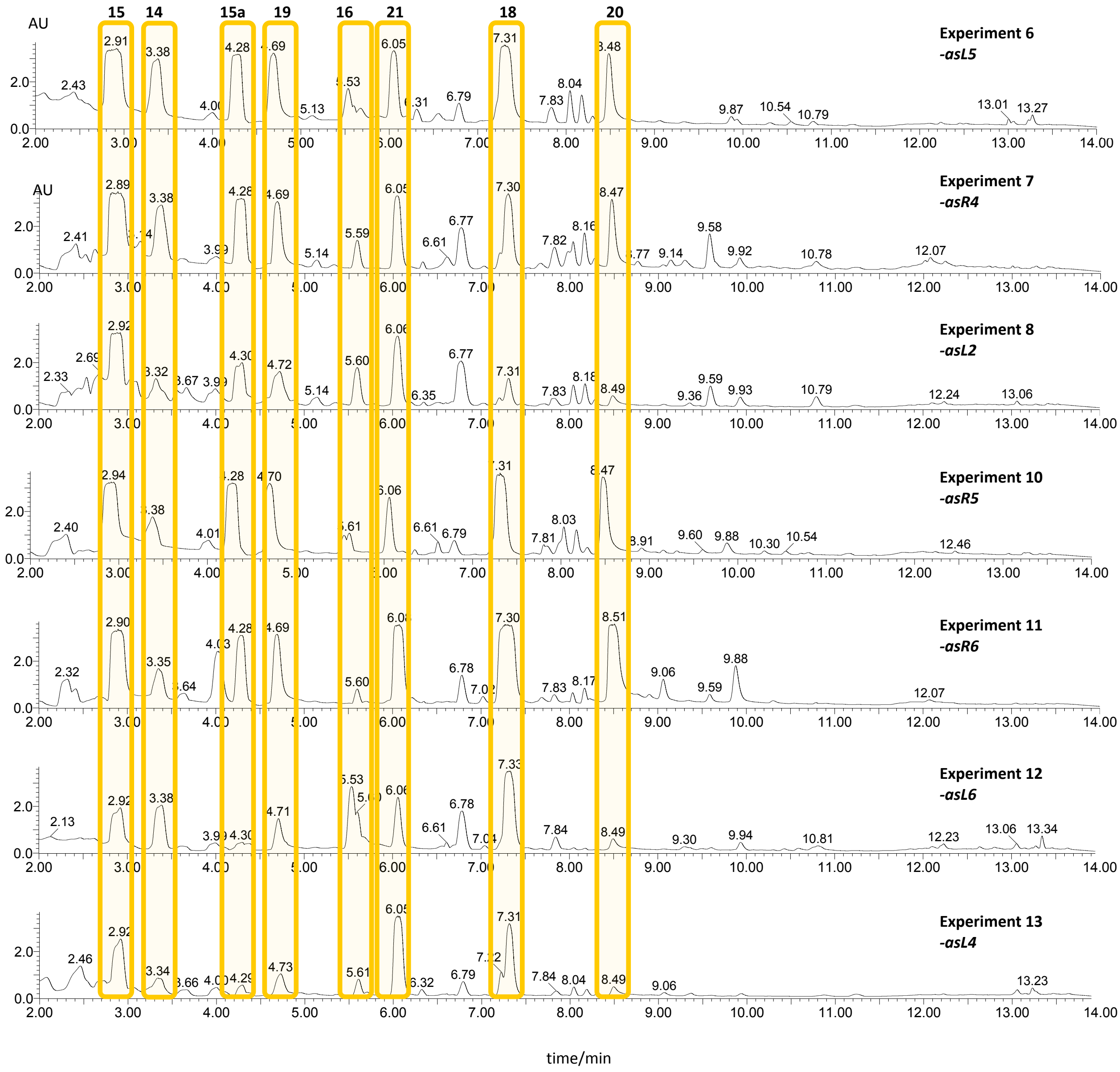

Supplementary Figure 52. DAD (Diode Array Detector) chromatogram of *A. oryzae* NSAR1 extracts measured with LCMS (Liquid Chromatography Mass Spectrometry) method 2. Extracts were obtained from transformants (DPY, 4 d) expressing different set of genes as indicated in Table 2 for experiments 6-8 and 10-13 (Main Text).

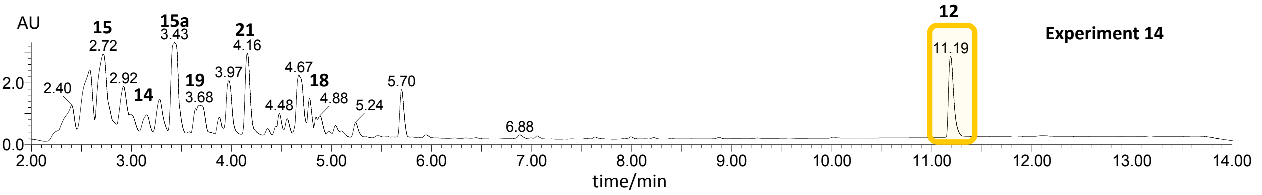

Supplementary Figure 53. DAD (Diode Array Detector) chromatogram of Experiment 14 (Table 2, main Text) showing the tropolone meroterpenoid **12**. LCMS (Liquid Chromatography Mass Spectrometry) method 1.

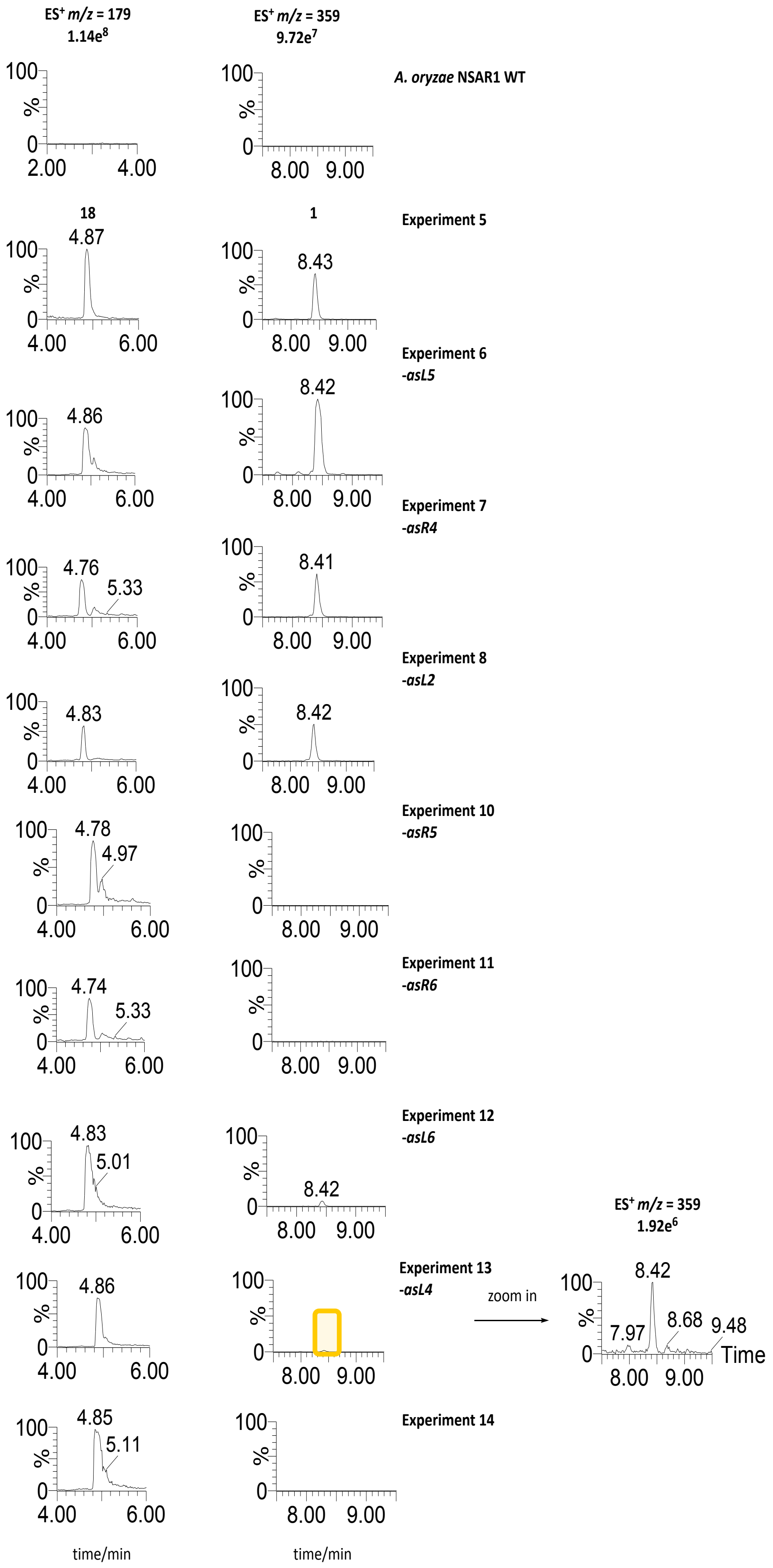

Supplementary Figure 54. Extracted ion chromatograms ES$^+$ *m/z* = 179 for **18** and *m/z* = 359 for **1** for all extracts obtained through KOe (Knockout by expression) experiments. Compound **18** shows the production of polyketide precursor and serves as an internal control. All data for *m/z* = 359 are shown at equivalent scales (9.72 e^7). Experiment 13 *asL4* is additionally shown at 1.92e^6 to show traces of **1** (Main text Table 2).

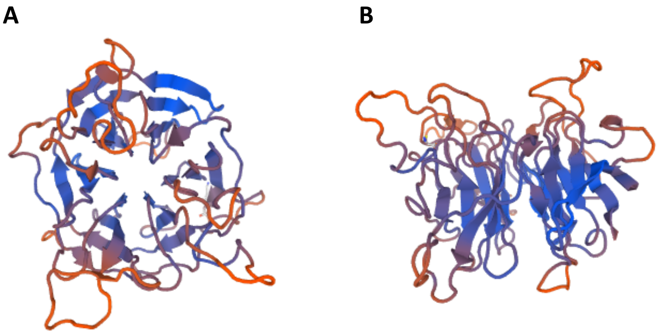

Supplementary Figure 55. Model of AsR5 computed with SWISS-MODEL and template 2p4o.1. **A**, Bottom view of predicted six-bladed propeller. **B**, Side view of predicted six-bladed propeller. Red low – blue high confidence.

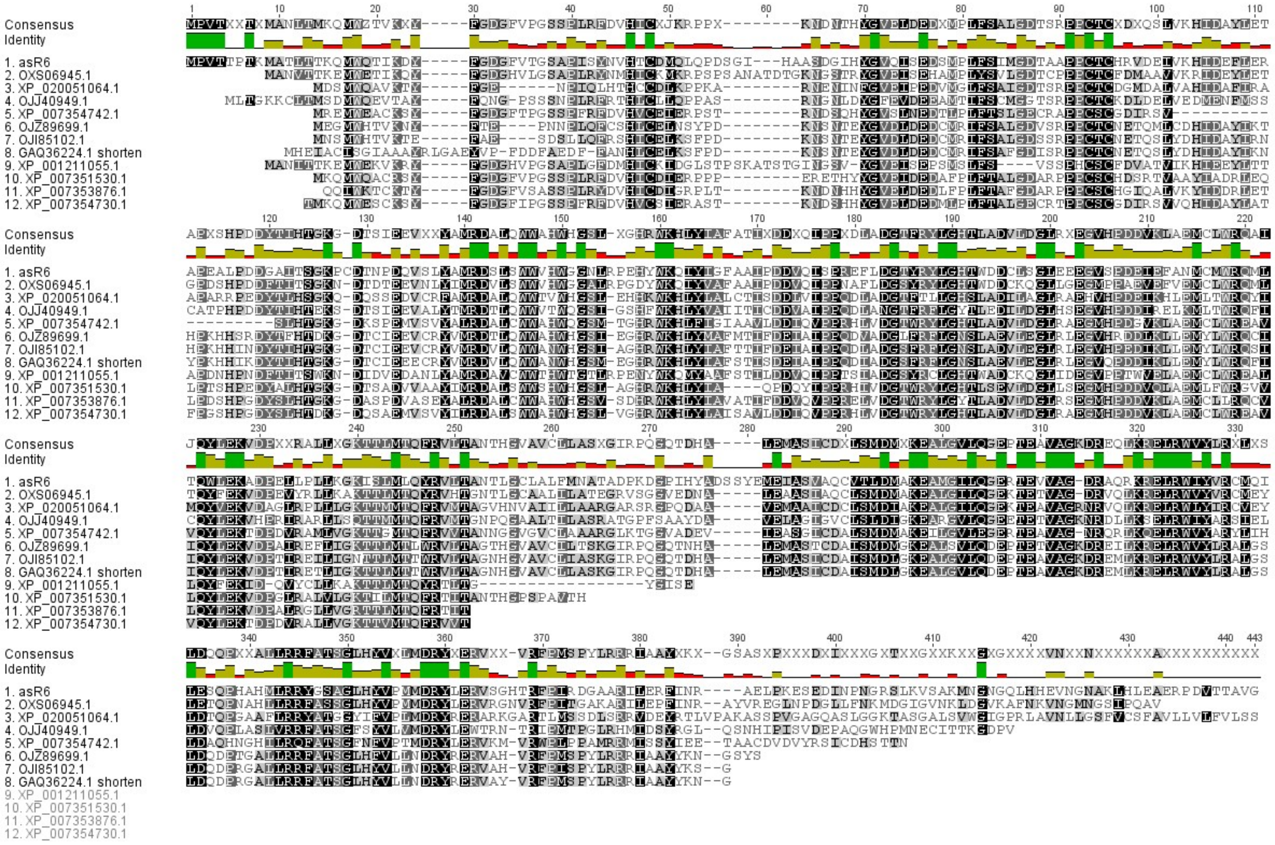

Supplementary Figure 56. Protein alignment of AsR6 and homologous sequences identified by BLASTp. Geneious 7.1.9 was used for the alignment.

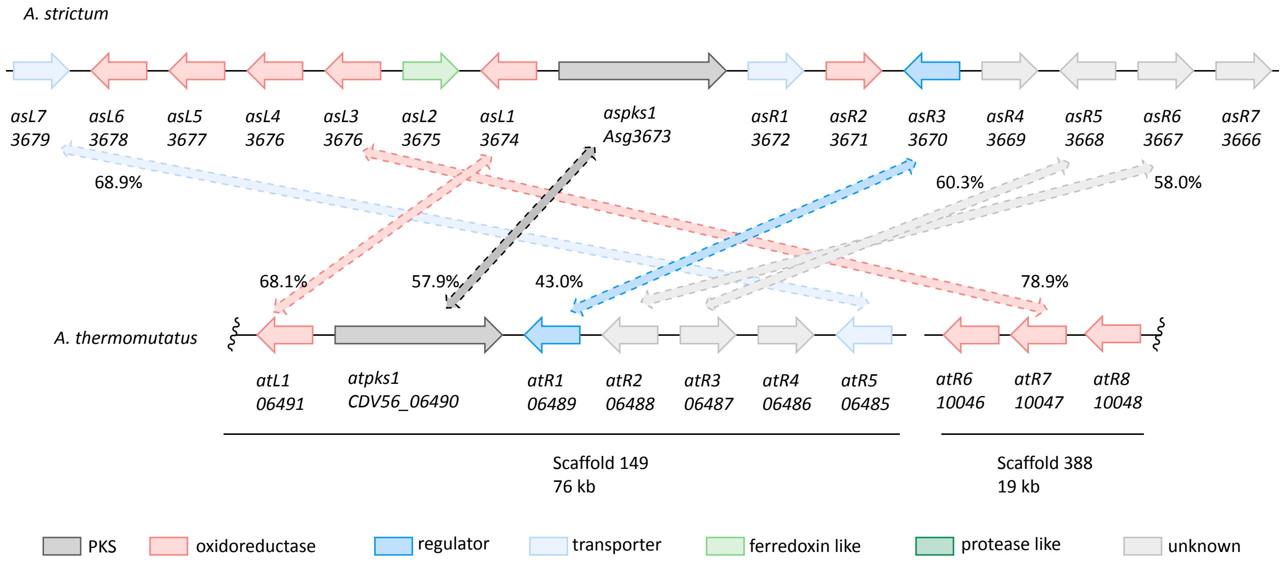

Supplementary Figure 57. ARTEMIS comparison of the xenovulene BGC (Biosynthetic Gene Cluster) with the unknown BGC from *Aspergillus thermomutatus*.

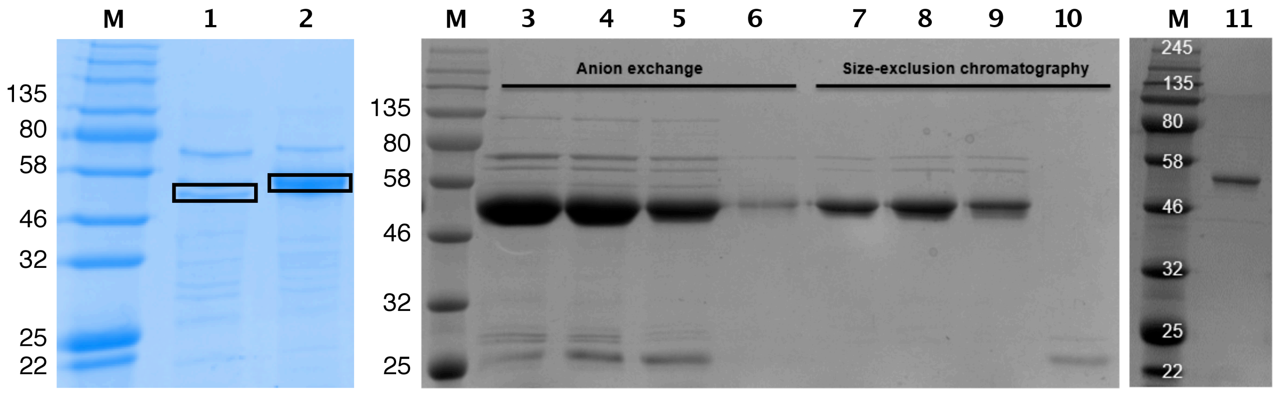

Supplementary Figure 58: Purification of AsR5 and AsR6. Lanes: **M**, markers, approximate size / KDa; **1**, AsR5 after nickel affinity chromatography; **2**, AsR6 after nickel affinity chromatography; **3-6**, fractions of AsR6 purification by Q-sepharose chromatography; **7-10**, fractions of AsR6 purification by size-exclusion chromatography; **11**, purified AsR6 used for *in vitro* assays. Boxed bands confirmed by MS sequencing.

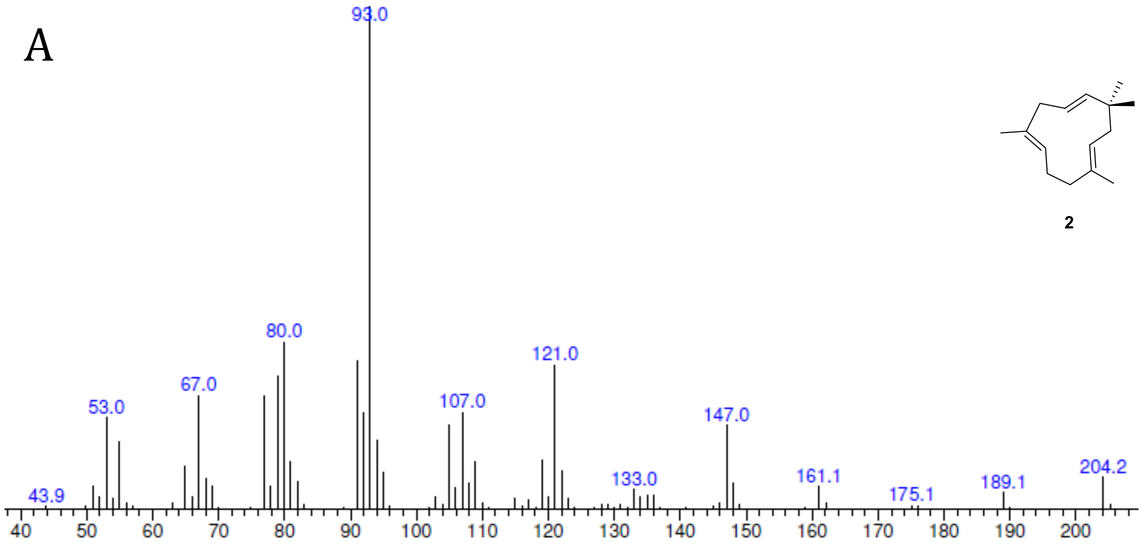

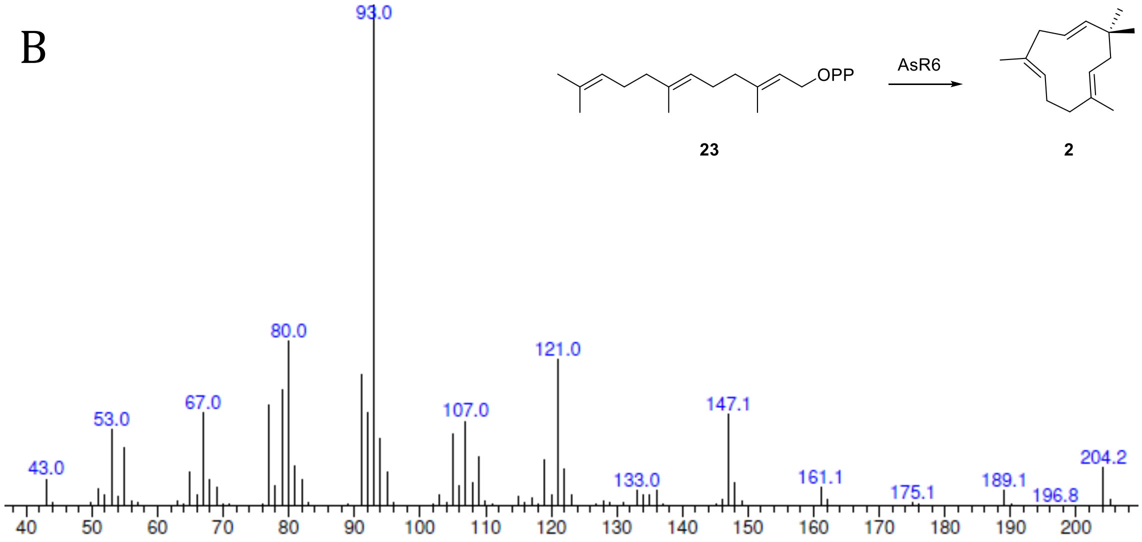

Supplementary Figure 59: GC-EI-MS (Gas Chromatography - Electron Impact - Mass Spectrometry) spectra of: **A**, humulene standard; and **B**, humulene produced by AsR6 upon incubation with Farnesylpyrophosphate **23** and $MgCl_2$.

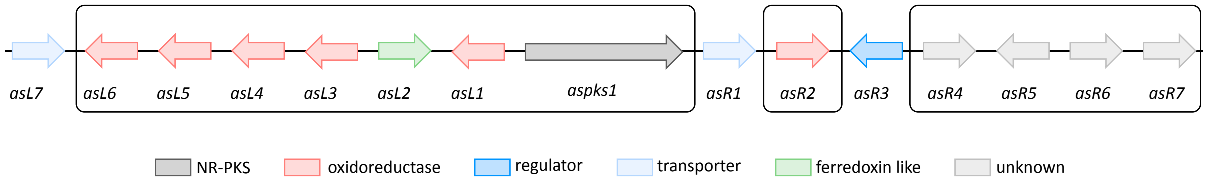

Supplementary Figure 60. *aspks1* BGC (Biosynthetic Gene Cluster), framed genes were selected for heterologous expression in *A. oryzae* NSAR1.

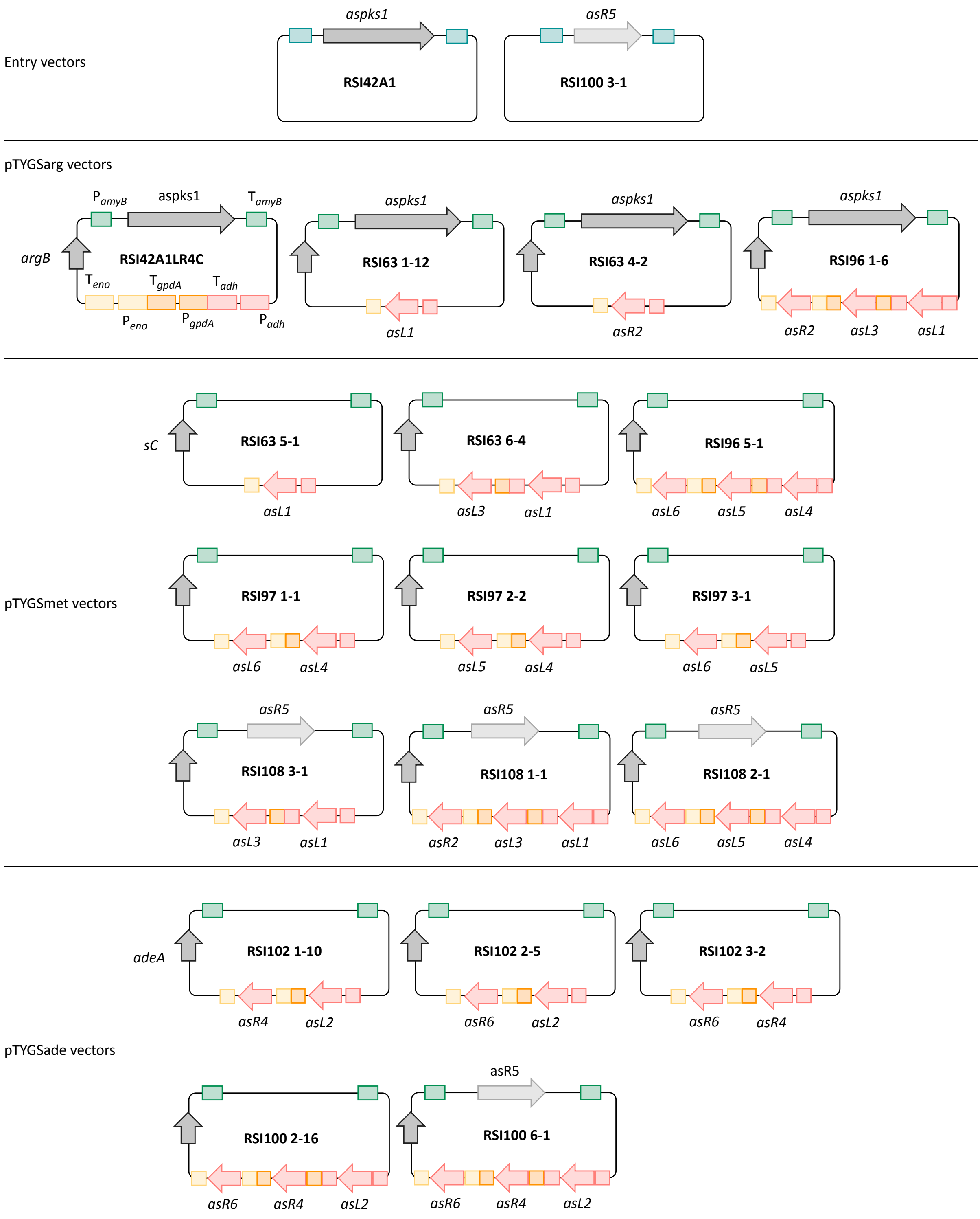

Supplementary Figure 61. Overview of vectors constructed in this work which used gateway entry and fungal expression (pTYGSarg, met, ade) vectors.

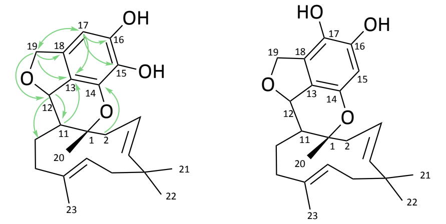

7 **8**

7
$C_{23}H_{30}O_4$
M = 370 g/mol

8
$C_{23}H_{30}O_4$
M = 370 g/mol

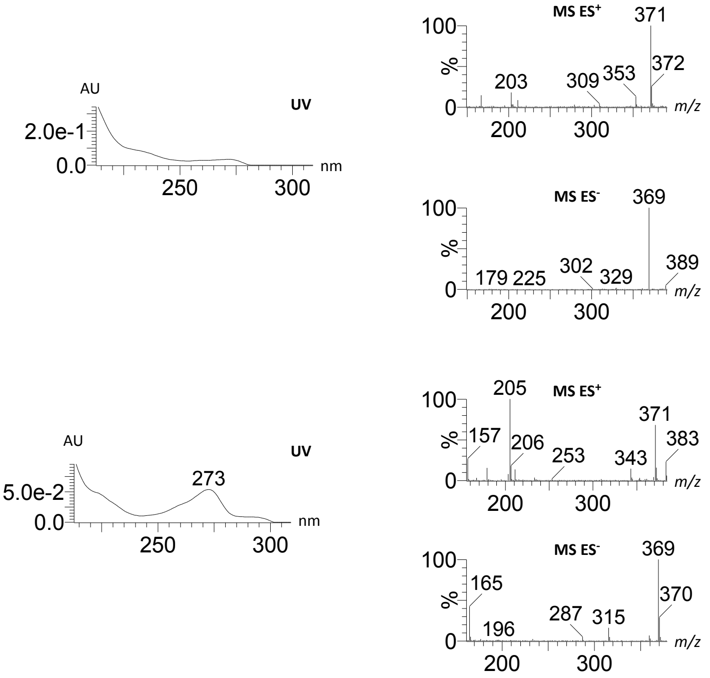

Supplementary Figure 62. Uv and MS data for compounds **7** and **8.** See Supplementary Table 1 for NMR data.

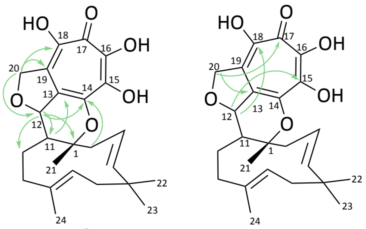

9 **9**

9
$C_{24}H_{30}O_6$
M = 414 g/mol

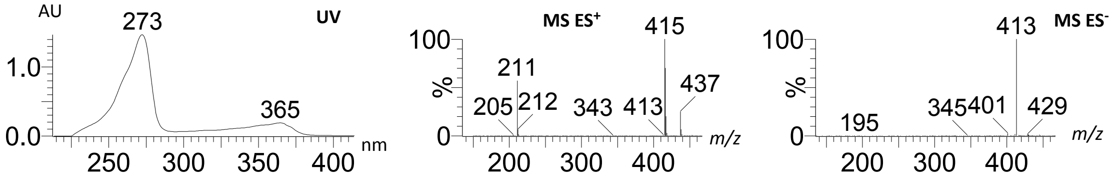

Supplementary Figure 63. Uv and MS data for compound **9.** See Supplementary Table 2 for NMR data.

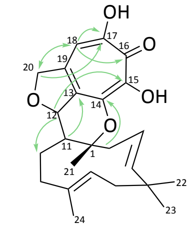

10

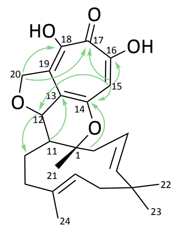

11

10
$C_{24}H_{30}O_5$
M = 398 g/mol

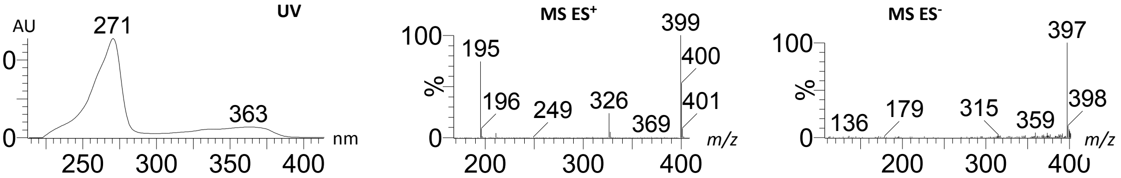

11
$C_{24}H_{30}O_5$
M = 398 g/mol

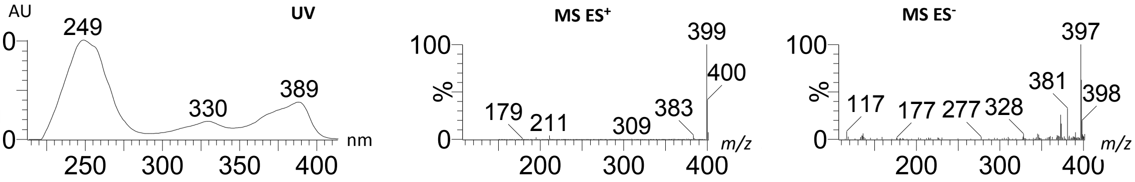

Supplementary Figure 64. Uv and MS data for compounds **10** and **11.** See Supplementary Table 3 for NMR data.

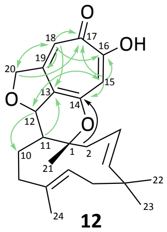

12

12
$C_{24}H_{30}O_4$
M = 382
g/mol

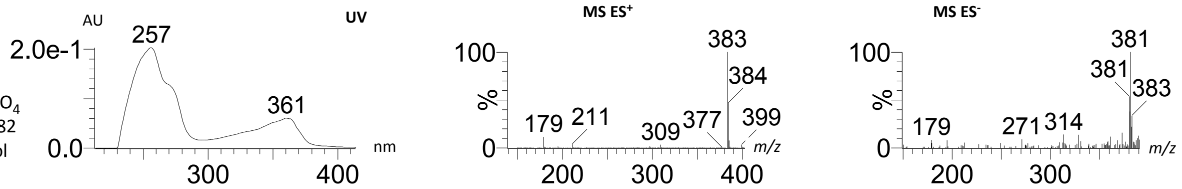

Supplementary Figure 65. Uv and MS data for compound **12.** See Supplementary Table 4 for NMR data.

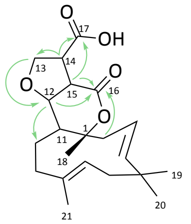

13

13

$C_{21}H_{30}O_5$
M = 362 g/mol

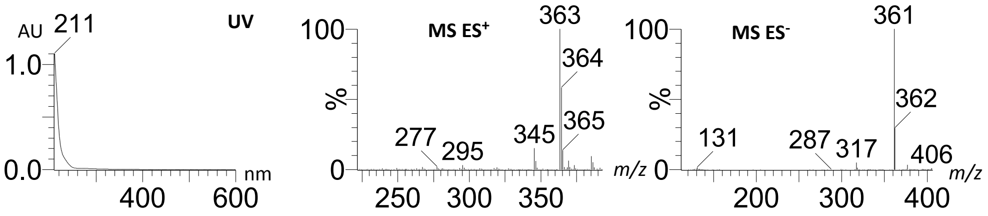

Supplementary Figure 66. Uv and MS data for compound **13.** See Supplementary Table 5 for NMR data.

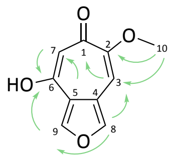

21

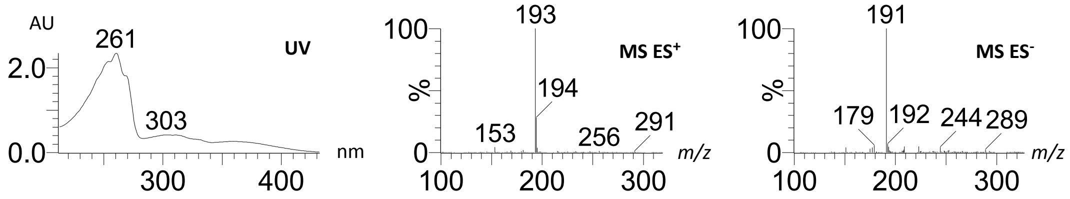

Supplementary Figure 67. Uv and MS data for compound **21.** See Supplementary Table 6 for NMR data.

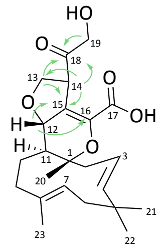

22

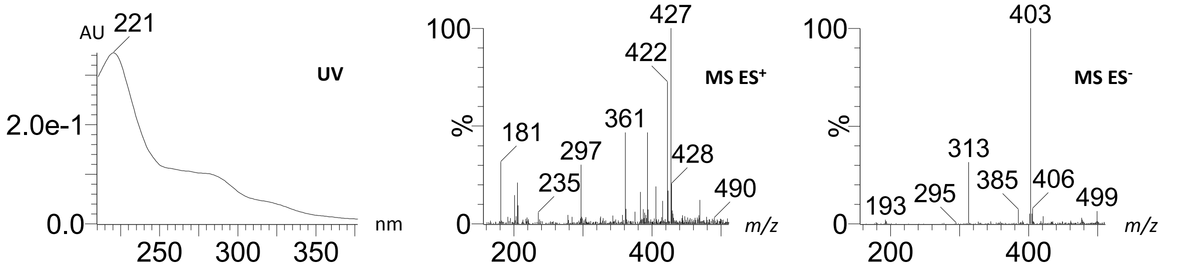

Supplementary Figure 68. Uv and MS data for compound **22.** See Supplementary Table 7 for NMR data.

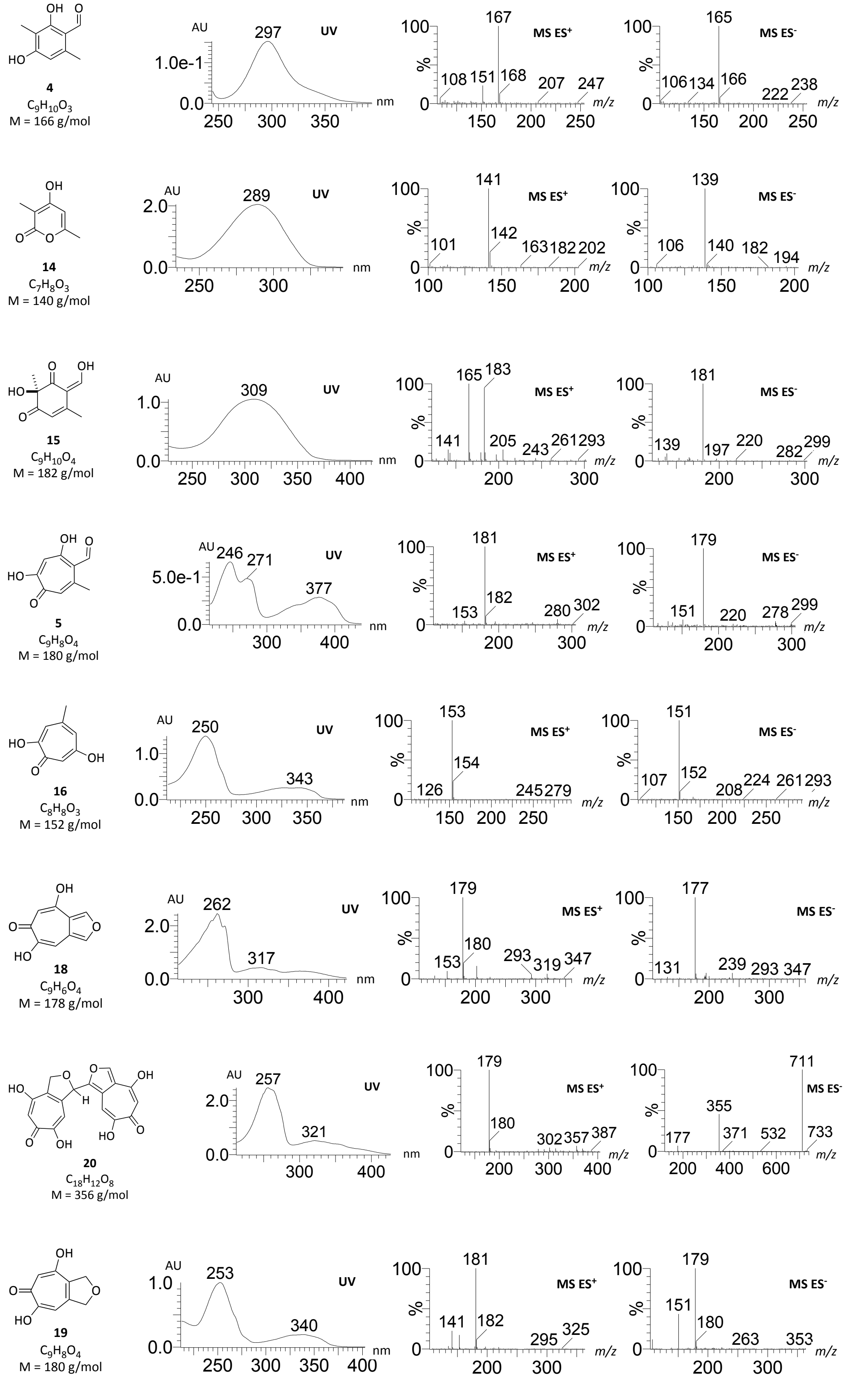

Supplementary Figure 69. UV (ultraviolet) and MS (Mass spectrometry) data for compounds shown. HRMS (High Resolution Mass Spectrometry) data is given in main text.

```
               1         10        20        30        40        50        60
               |         |         |         |         |         |         |
 CCT72694.1    ------------------------------------------------------------
 AB247331.1    ---------MERQSMALVGDKEEIIRKSFEYHPTVWGDYFIRNYSCLPLEKECMIKRVEE
 KP398851.1    ------------------------------------------------------------
 AAF13264.1    ------------------------------------------------------------
       AsR6    ------MPVTTPTKMATLTTKQ------------MW----------------------QT

 CCT72694.1    ------------------------------------------------------------
 AB247331.1    LKDRVRNLFEETHDVLQIMILVDSIQLLGLDYHFEKE-ITAALRLIYEADVENYGLYEVS
 KP398851.1    ------------------------------------------------------------
 AAF13264.1    ------------------------------------------------------------
       AsR6    IKD----------------------------YFGDGFVTGSAPISYNVHTCDMQL----

CCT72694.1    -MVPSLITPPPSRSGEATPQKDACLNPVNIAEPEGHW----------IKLPEALF-SSIM
 AB247331.1    LRFRLLRQHGYNLSPDVFNKFKDDKGRFLPTLNGDAKGLLNLYNAAYLGTHEETILDEAI
 KP398851.1    ---------MPSRIDMAITETP--MARRKNNDPPRSH------------MVGRLH-PSGL
 AAF13264.1    -----------MKKPNGTNGAS-----SSLEPPPSTF----------QPL---CH-PLVE
       AsR6    ---------QPDSGIHAASDGIHYGVQISEDSMPL-----------FSIMGDTAAPPCTC

 CCT72694.1    AVEPEVNPLYRT-------------------SKALSDEWLKTALRMN-------------
 AB247331.1    SFTKCQLESLLGELEQPLAIEVSLFLETPLYRRTRRLLVRKYIPIYQEKVMRN----DTI
 KP398851.1    RITMEVNNYFLN-------------------NWPFPDE--RTKLQFV-------------
 AAF13264.1    EVSKEVDGYFLQ-------------------HWNFPNE--KARKKFV-------------
       AsR6    HRVDEIVKH------------IDEFLERAPE--ALPDDG----------AITSGKPCDTN

 CCT72694.1    ---------------------------------DKTAVIWSRLDIAYMSAICAPHADLET
 AB247331.1    LELAKLDFNLLQSLHQEEVKKITIWWNDLALTKSLKFARDRVVECYYWIVAVYFEPQYSR
 KP398851.1    ------------------------------------AEGLPTWHLCQY-----PTAREDR
 AAF13264.1    ------------------------------------AAGFSRVTCLYF-----PKALDDR
       AsR6    PDQVSL-YAMRDSLS---------WWVHW--------GGNLRPEHYWKQIYIGFAAIPDD

 CCT72694.1    LKLM-NDWNGWVFAFDDPFD---------------EGTFANDPIK-AAEEVIYTLATLDN
 AB247331.1    ARVITSKAISLMSIMDDIYD----------------------------------------
 KP398851.1    VGAA-CRFMVMLFLIDDLLE----------------------HLP-LKEGSSYN----ER
 AAF13264.1    IHFA-CRLLTVLFLIDDLLE----------------------YMS-FEEGSAYN----EK
       AsR6    VQISPREFLDGTYRYLGHTWDDCLSGLEEEGVSPDEIEFANMCMWRQMLTQWLEKADPE-

 CCT72694.1    IHPVVSPDENPLRHT------LQSCW--MRFRERSSPSLQYRWKKHLTMYCVGVL-QQVG
 AB247331.1    ------------------------------------------------------------
 KP398851.1    LISIVKGETAPAQDSPIEKV-VLNVWEDMRSIDKRLS---DDIEEPTFAF----W-RSQN
 AAF13264.1    LIPISRGDVLPDRSIPVEYI-IYDLWESMRAHDREMA---DEILEPVFLF----M-RAQT
       AsR6    LLPLLKG--------------------------KISLMLQYRVLTANTLGCLALFMNATA

 CCT72694.1    VQHRATR-----------------------------------------------------
 AB247331.1    ----------------------NYSTLEESRLLTEAIERWEPQAVDCVPEYLKDFYLKLL
 KP398851.1    DKDRLAK-----------------------------------------------------
 AAF13264.1    DRTRARP-----------------------------------------------------
       AsR6    DPKDGPIHYADSSYEMEIASVAQCVTLDMAK---EAMGILQGERTEVVA-----------

 CCT72694.1    ------------------------------------------------PTIEEYMD----
 AB247331.1    KTYKDFEDELEPNEKYRIPYLQ--EEIKVLSRAYFQEAKWGVE--RYVPALEEHLL----
 KP398851.1    ------------------------------------------------KSVAEYLK----
 AAF13264.1    ------------------------------------------------MGLGGYLE----
       AsR6    ------GDRAQRKRELRWIYVRCMQILESQPHAHMLR-RYGSAGLHYVPMMDRYLERVSG

 CCT72694.1    MRAGCVGAYPCIGLMEFAEGIDIPQNVMDHPSMQAISRITCDL-VTLQNDLCSYRKDL--
 AB247331.1    VSLITAGYFAVACASYVGLGEDATKETFEWVASSPKILKSCSIHCRLMDDITSHQREQ--
 KP398851.1    YRELDVASAVLLATQRFQDAIYLSEEELASVA---DLELNYTHHISVINDVMSWDKELRA
 AAF13264.1    YRERDVGKELLAALMRFSMGLKLSPSELQRVR---EIDANCSKHLSVVNDIYSYEKELYT
       AsR6    HTRFPIRDGAARILERFINRAELPKES---------------------EDINPNGRSLKV


 CCT72694.1    ------IQGEESNIIFILKDQGMTDQQAVDQIGEMLYDCYRRW----HMALANLPFWGEG
 AB247331.1    ------ERDHFASTVESYMKEHGTSAKVACEKLQVMVE--QKWKDLNEECLRPTQVARPL
 KP398851.1    SKDIAAEGSIVSNIVQTLADESGLDYDGAKRTCWVM-I--REW-ERQHDAIVQQKLQEGC
 AAF13264.1    SKTAHSEGGILCTSVQILAQEADVTAEAAKRVLFVM-C--REW-ELRHQLLVARLSAEGL
       AsR6    SAKMNGNGQLHHEV----------------------------------------------

 CCT72694.1    IDRDVIKFVTGCRNIALGNLHWSLYTFRYLGNDGPEVKRTRMMKLP
 AB247331.1    IEI-ILNLSRAMEDIYKHKDTYTNSNTRMKDNVSLIFVESFLI---
 KP398851.1    SDA-LKTYMEGIELQMSGNELWSRTTHRY---NAPHLK--------
 AAF13264.1    ETPGLAAYVEGLEYQMSGNELWSQTTLRY----SVVVD--------
       AsR6    ----------------NGNAKLHLEAER------PDVTTAVG----
```

Supplementary Figure 70. Multiple alignment of AsR6 with known sesquiterpene cyclases. Humulene synthase from *Z. zerumbet* (AB247331.1), *F. fujikorii* koraiol synthase Ffsc4 (CCT72694.1), *C. acutatum* CaTPS (KP398851.1) and aristolochene synthase from *A. terreus* (AAF13264.1)

Supplementary Tables

Supplementary Table 1. Assignment of NMR (Nuclear Magnetic Resonance) data for **7** and **8**. See supplementary Figure 62 for numbering scheme.

Position	δ_C/ppm 7	δ_H/ppm (*J* in Hz) 7 (in $CDCl_3$)	HMBC (H to C)	δ_H/ppm (*J* in Hz) 8
1	86.8	-	-	-
2	43.1	2.34 (dd, 1H, 14.7, 10.5)	3, 4, 5, 14	2.28 (m)
		2.60 (ddd, 1H, 14.7, 2.3, 2.3)	1, 2, 3, 4, 21	2.50 (ddd, 14.6, 2.3, 2.3)
3	119.7	4.93 (ddd, 1H, 15.8, 10.5, 2.5)	1, 2, 5	4.96 (m)
4	143.2	5.13 (dd, 1H, 15.8, 1.8)	2, 3, 5, 20, 21	5.10 (dd, 1H, 15.9, 1.7)
5	38.4	-	-	-
6	41.6	1.71 (m, 1H)	4, 5, 7, 8, 20, 21	1.72 (m)
		2.15 (m, 1H)	5, 7, 8, 21	2.07-2.22 (m)
7	123.4	5.00 (m, 1H)	5, 23	4.98-5.03 (m)
8	136.5	-		-
9	37.9	2.11 (m, 1H)	7, 8, 10, 11, 23	2.07-2.22 (m)
		2.22 (1H)	7, 8, 10, 11, 23	2.07-2.22 (m)
10	29.1	1.29 (m, 1 H)	1, 8, 9, 11, 12, 13	contamination at same δ_H
		1.65 (m, 1H)	8, 9, 11, 12	1.69 (m)
11	43.4	1.73 (m, 1H)	1, 2, 9, 10, 12, 13	contamination at same δ_H
12	82.3	4.78 (m, 1H)	10, 11, 13, 14, 18	4.75 (br d)
13	118.7	-	-	-
14	138.5	-	-	-
15	128.8	-	-	
16	145.3	-	-	-
17	100.7	6.43 (s, 1H)	13, 15, 16, 19	-
18	131.6	-	-	-
19	73.4	4.80 (m, 1H)	12, 13, 17, 18	4.98-5.03 (m)
		5.01 (m, 1H)	13, 17, 18	5.08
20	22.8	1.25 (s, 3H)	1, 2, 11	1.25 (s)
21	30.3	1.03 (s, 3H)	4, 6, 5, 22	1.04 (s)
22	24.4	0.95 (s, 3H)	4, 6, 5, 21	0.95 (s)
23	17.1	1.59 (s, 3H)	7, 9, 8	1.59 (s)

Supplementary Table 2. NMR (Nuclear Magnetic Resonance) assignments for **9.** See Supplementary Figure 63 for numbering scheme.

Position	δ_C/ppm 9	δ_H/ppm (*J* in Hz) 9 in $CDCl_3$	HMBC (H to C)
1	87.1	-	-
2	43.6	2.40 (dd, 1H, 14.8, 10.3)	3, 4, 21
		2.75 (d, 1H, 14.8)	1, 3, 4, 11, 21
3	119.0	4.91 (m, 1H)	1, 2, 5
4	144.1	5.17 (dd, 1H, 13.0, 4.9)	2, 5, 22, 23
5	38.5	-	-
6	41.8	1.75 (dd, 1H, 13.0, 4.9)	4, 5, 7, 8, 23
		2.16 (m, 1H)	4, 5, 7, 8, 22, 23
7	123.9	5.04 (m, 1H)	5, 6, 24
8	136.2	-	-
9	37.9	2.13 (m, 1H)	7, 8, 10, 11, 24
		2.31 (m, 1H)	7, 8, 10, 11, 24
10	28.4	1.38 (m, 1 H)	1, 8, 9, 11, 12
		1.70 (m, 1H)	8, 9, 11,12
11	40.9	2.05 (br dd, 1H, 10.8, 8.2)	1, 2, 9, 10, 12, 13, 21
12	84.5	4.91 (m, 1H)	1, 11, 10, 13, 14, 15, 18, 19
13	123.5	-	-
14	142.4	-	-
15	144.6	-	-
16	150.6	-	-
17	160.3	-	-
18	146.4	-	-
19	127.4	-	-
20	72.6	5.05 (m, 1H)	13, 14, 17, 18, 19
		5.21 (dd, 1H, 13.7, 1.1)	12, 13, 16, 17, 18, 19
21	23.0	1.33 (s, 3H)	1, 2, 11
22	29.7	1.02 (s, 3H)	4, 5, 6 23
23	24.4	0.93 (s, 3H)	4, 5, 6, 22
24	17.2	1.60 (s, 3H)	7, 9, 8

Supplementary Table 3. NMR (Nuclear Magnetic Resonance) assignment of compounds **10** and **11**. See Supplementary Figure 64 for numbering scheme.

Position	δ_C/ppm 10	δ_H/ppm (*J* in Hz) 10 in $CDCl_3$	HMBC (H to C) 10	δ_C/ppm 11	δ_H/ppm (*J* in Hz) 11 in $CDCl_3$	HMBC (H to C) 11
1	87.5	-	-	85.8	-	-
2	43.7	2.37 (dd, 1H, 14.7, 10.4)	3, 4, 14	43.3	2.31 (m, 1H)	3, 4, 14
		2.81 (d, 1H, 15.0)	1, 3, 4, 5, 11, 21		2.60 (ddd, 1H, 14.9., 3.9, 1.9)	1, 3, 4, 5, 11, 21
3	119.4	4.93 (ddd, 1H, 15.8, 10.3,	1, 2, 5	119.2	4.91 (ddd, 1H, 15.8, 10.5,	1, 2, 5
4	143.9	2.8)	1, 5, 22, 23	143.8	2.6)	2, 5, 22, 23
5	38.5	5.16 (dd, 1H, 15.8, 1.7)	-	38.5	5.16 (dd, 1H, 15.8, 1.8)	-
6	41.7	-	4, 5, 7, 8, 23	41.7	-	4, 5, 7, 8, 23
		1.75 (br dd, 1H, 13.0, 4.9)	4, 5, 7, 8, 22, 23		1.75 (dd, 1H, 13.0, 4.7)	5, 7, 8, 22, 23
7	124.0	2.16 (m, 1H)	5, 24	123.8	2.16 (m, 1H)	5, 6, 24
8	136.2	5.05 (m, 1H)	-	136.3	5.04 (m, 1H)	-
9	38.0	-	7, 8, 10, 11, 24	38.0	-	7, 8, 10, 11, 24
		2.13 (m, 1H)	7, 8, 10, 11, 24		2.14 (m, 1H)	7, 8, 10, 11, 24
10	28.3	2.30 (br dd, 1H, 12.2, 12.2)	1, 8, 9, 11, 12	28.3	2.28 (m, 1H)	1, 8, 9, 11, 12
		1.41 (m, 1 H)	8, 9, 11, 12		1.37 (m, 1 H)	8, 9, 11, 12
11	41.1	1.68 (m, 1H)	1, 2, 9, 10, 12, 13,	40.5	1.66 (m, 1H)	1, 2, 9, 10, 12, 13,
12	85.2	2.09 (m, 1H)	21	84.3	1.96 (br dd, 1H, 10.7, 8.2)	21
13	127.7	4.99 (m, 1H)	10, 11	122.8	4.85 (br d, 1H, 10.7)	10, 11, 13, 15, 18,
14	148.2	-	-	152.6	-	19
15	149.3	-	-	112.3	-	-
16	164.2	-	-	159.4	7.16 (s, 1H)	-
17	159.8	-	-	165.9	-	12, 13, 14, 16, 17,
18	108.9	-	-	147.1	-	19
19	138.2	7.12 (s, 1H)	13, 14, 16, 17, 19,	134.5	-	-
20	75.6	-	20	72.5	-	-
		5.00 (m, 1H)	-		5.05 (dd, 1H, 14.1, 2.3)	-
21	23.1	5.08 (dd, 1H, 13.3, 3.3)	12, 13, 16, 18, 19	22.8	5.21 (dd, 1H, 14.1, 1.0)	-
22	29.7	1.37 (s, 3H)	13, 19	30.0	1.33 (s, 3H)	13, 14, 18
23	24.4	1.02 (s, 3H)	1, 2, 11	24.3	1.02 (s, 3H)	12, 13, 17, 18, 19
24	17.2	0.92 (s, 3H)	4, 6, 5, 23	17.1	0.93 (s, 3H)	1, 2, 11
		1.61 (s, 3H)	4, 6, 5, 22		1.60 (s, 3H)	4, 6, 5, 23
			7, 9, 8			4, 6, 5, 22
						7, 9, 8

Supplementary Table 4. NMR (Nuclear Magnetic Resonance) assignment for **12.** See Supplementary Figure 65 for numbering scheme.

Position	δ_C/ppm 12	δ_H/ppm (*J* in Hz) 12 in $CDCl_3$	HMBC (H to C)
1	86.7	-	-
2	43.1	2.31 (dd, 1H, 14.6, 10.4)	3, 4, 14
		2.63 (ddd, 1H, 14.7, 3.9, 1.9)	1, 3, 4, 5, 11, 21
3	119.2	4.91 (m, 1H)	2, 5
4	143.9	5.17 (dd, 1H, 15.8, 1.8)	1, 2, 5, 22, 23
5	38.5	-	-
6	41.7	1.75 (dd, 1H, 12.9, 4.7)	4, 5, 7, 8, 22
		2.16 (m, 1H)	5, 7, 8, 22, 23
7	123.8	5.05 (m, 1H)	5, 6, 24
8	136.3	-	-
9	38.0	2.12 (m, 1H)	7, 8, 10, 11, 24
		2.26 (m, 1H)	7, 8, 10, 11, 24
10	28.3	1.35 (m, 1 H)	1, 8, 9, 11, 12, 13
		1.64 (m, 1H)	8, 9, 11, 12
11	40.5	1.94 (br dd, 1H, 10.7, 8.2)	1, 2, 9, 10, 12, 13, 21
12	84.2	4.80 (dd, 1H, 10.6, 2.1)	10, 11, 13, 14, 15, 18, 19
13	121.3	-	-
14	158.9	-	-
15	111.0	6.96 (s, 1H)	12, 13, 14, 16, 17
16	166.1	-	-
17	173.5	-	-
18	113.1	6.91 (s, 1H)	13, 16, 17, 19, 20
19	152.1	-	-
20	75.2	4.90 (m, 1H)	12, 13, 17, 18, 19
		4.97 (m, 1H)	13, 14, 17, 18, 19
21	22.7	1.26 (s, 3H)	1, 2, 11
22	30.0	1.04 (s, 3H)	4, 6, 5, 23
23	24.3	0.97 (s, 3H)	4, 6, 5, 22
24	17.1	1.61 (s, 3H)	7, 9, 8

Supplementary Table 5. NMR (Nuclear Magnetic Resonance) assignments for **13**. See Supplementary Figure 66 for numbering scheme.

Position	δ_C/ppm 13	δ_H/ppm (*J* in Hz) 13 in CD_3OD	HMBC (H to C)
1	88.6	-	-
2	44.1	2.33 (m, 1H)	1, 3, 4, 5
		2.46 (ddd, 1H, 14.7, 2.2, 2.2)	1, 3, 4, 11
3	120.7	5.11 (m, 1H)	1, 2, 5
4	144.8	5.23 (dd, 1H, 15.9, 1.8)	2, 5, 19, 20
5	39.2	-	-
6	42.5	1.79 (br dd, 1H, 13.0, 4.9)	4, 5, 7, 8, 19
		2.25 (m, 1H)	4, 5, 7, 8, 19
7	124.1	5.09 (m, 1H)	5, 21
8	137.7	-	
9	37.9	2.04 (br dd, 1H, 12.8, 7.2)	7, 8, 10, 11, 21
		2.28 (m, 1H)	7, 8, 10, 11, 21
10	31.4	1.42 (m, 1 H)	1, 8, 9, 11, 12, 13
		1.56 (m, 1H)	8, 9, 11, 12
11	43.4	1.96 (dd, 1H, 8.3, 8.3)	1, 2, 9, 10, 12, 13, 18
12	85.8	4.21 (m, 1H)	10, 16
13	70.7	3.73 (m, 1H)	12, 17
		4.23 (m, 1H)	12, 14
14	49.1	3.55 (ddd, 1H, 8.1, 8.1, 5.3)	13, 15, 16, 17
15	47.8	3.74 (m, 1H)	16, 17
16	174.7	-	-
17	175.9	-	-
18	22.1	1.37 (s, 3H)	1, 2
19	24.6	1.08 (s, 3H)	4, 6, 5, 20
20	30.5	1.07 (s, 3H)	4, 6, 5, 19
21	17.3	1.65 (s, 3H)	7, 9, 8

Supplementary Table 6. NMR (Nuclear Magnetic Resonance) assignment of **21**. See Supplementary Figure 67 for numbering scheme.

Position	δ_C/ppm 21	δ_H/ppm (*J* in Hz) 21 in DMSO-d6	HMBC (H to C)
1	176.9	-	-
2	152.6	-	-
3	100.8	6.64 (s, 1 H)	1, 2, 5, 8
4	119.1	-	-
5	122.4	-	-
6	165.1	-	-
7	109.7	5.86 (s, 1 H)	1, 2, 5, 6, 9
8	143.0	8.14 (d, 1 H, 1.9)	2, 3, 4, 5, 9
9	144.0	8.17 (d, 1 H, 1.9)	4, 5, 7, 8
10	56.3	3.65 (s, 3H)	2, 3

Supplementary Table 7. NMR (Nuclear Magnetic Resonance) Assignment of **22**. See Supplementary Figure 67 for numbering scheme.

Position	δ_C/ppm 22	δ_H/ppm (*J* in Hz) 22 in CD_3OD	HMBC (H to C)
1	83.3	-	-
2	43.6	2.25 (m, 1H)	3, 11
		2.60 (bd, 1H, 14.2)	1, 3, 4, 11
3	122.5	5.16 (m, 1H)	2, 5, 21
4	143.1	5.15 (m, 1H)	2, 3, 5, 21
5	39.3	-	-
6	42.8	1.75 (dd, 1H, 12.6, 4.6)	4, 5, 7, 8, 21
		2.20 (m, 1H)	4, 7, 21, 22
7	124.3	5.10 (m, 1H)	6, 9, 23
8	137.7	-	-
9	39.2	2.01 (m, 1H)	7, 8, 10, 11, 23
		2.32 (m, 1H)	7, 8, 10, 11, 23
10	29.6	1.40 (m, 1 H)	8, 9
		1.49 (m, 1H)	8, 9, 12
11	41.0	1.87 (m, 1H)	1, 2, 9, 10, 12, 20
12	84.3	4.12 (dd, 1H, 10.0, 1.7)	10, 11, 15, 16
13	72.5	3.91 (m, 1H)	12, 15, 18
		3.93 (m, 1H)	12, 15, 18
14	53.7	4.09 (m, 1H)	13, 15, 18
15	116.9	-	-
16	143.2	-	-
17	170.3	-	-
18	211.2	-	-
19	67.3	4.17 (d, 1H, 18.1)	18
		4.31 (d, 1H, 18.1)	18
20	22.4	1.14 (s, 3H)	1, 2, 11
21	24.6	1.09 (s, 3H)	4, 5, 6, 22
22	30.8	1.04 (s, 3H)	4, 5, 6, 21
23	17.2	1.62 (s, 3H)	6, 7, 9

Supplementary Table 8. ^{1}H NMR (Nuclear Magnetic Resonance) data for Compound **5.**[1]

Position	δ_H 5 in DMSO-d6
3	6.28 (s, 1H)
6	6.37 (s, 1H)
8	2.46 (s, 3H)
9	9.82 (s, 1H)

Supplementary Table 9. NMR (Nuclear Magnetic Resonance) data for other compounds**.**

Compound	^{1}H NMR	^{13}C NMR	ref
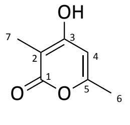 **14**	(500 MHz, CD_3OD), δ_H: 5.98 (s, 1H, 4-C*H*), 2.19 (s, 3H, 6-CH_3), 1.84 (s, 3H, 7-CH_3).	(125 MHz, CD_3OD), δ_C: 169.2 (*C*-1), 168.9 (*C*-3), 161.3 (*C*-5), 102.0 (*C*-4), 98.5 (*C*-2), 19.5 (*C*-6), 8.2 (*C*-7).	1
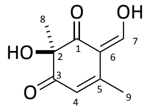 **15**	(400MHz, d_6-DMSO), δ_H: 8.13 (s, 1H, 7-C*H*OH), 5.64 (s, 1H, 4-C*H*), 2.22 (s, 3H, 8-CH_3), 1.30 (s, 3H, 9-CH_3).		1
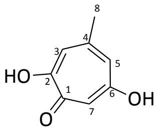 **16**	(500 MHz, CD_3OD), δ_H: 6.92 (s, 1H, 3-C*H*), 6.78 (s, 1H,7-C*H*), 6.69 (s, 1H, 5-C*H*), 2.38 (s, 3H, 8-CH_3).	(125 MHz, CD_3OD), δ_C: 174.2 (1-*C*O), 169.5 (6-*C*OH), 166.4 (2-*C*OH), 150.4 (4-*C*CH3), 122.0 (5-*C*H), 118.1 (3-*C*H), 113.7 (7-*C*H), 27.8 (8-*C*H_3).	1
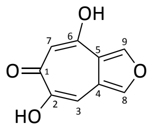 **18**	(500 MHz, CD_3OD), δ_H: 8.20 (dd, 1H, $^4J_{HH}$ = 1.9, $^5J_{HH}$ = 0.8, 9-C*H*OCH), 8.01 (d, 1H, $^4J_{HH}$ = 1.9, 8-C*H*OCH), 6.89 (s, 1H, $^5J_{HH}$ = 0.8, 3-C*H*). ^{13}C NMR (125 MHz, CD_3OD), δ_C: 182.0 (1-*C*O), 168.4 (6-*C*OH), 150.8 (2-*C*OH), 145.8 (9-*C*HOCH), 143.6 (8-*C*HOCH), 121.7 (4-*C*), 121.6 (4-*C*), 107.5-107.3 (7-*C*D), 103.6 (3-*C*H).		1
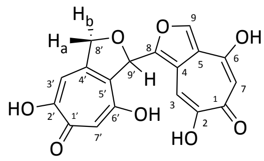 **20**	(500 MHz, CD_3OD), δ_H: 8.11 (s, 1H, 9-C*H*), 7.00 (s, 1H, 3-C*H*), 6.86 (s, 1H, 3'-C*H*), 6.60 (d, 1H, J_{HH} = 2.4, 9'-C*H*OC), 5.32 (dd, 1H, J_{HH} = 14.0, 3.6, 8'-C*H*$_2$OC, H$_a$), 5.15 (d, 1H, J_{HH} = 13.8, 8'-C*H*$_2$OC, H$_b$).	(125 MHz, CD_3OD), δ_C: 182.2 (1-*C*O), 173.4 (1'-*C*O), 167.6 (2'-*C*OH), 167.0 (6'-*C*OH), 165.6 (6-*C*), 153.0 (8-*C*), 150.7 (2-*C*OH), 148.9 (4'-*C*), 144.2 (9-*C*H), 128.6 (5'-*C*), 122.0 (5-*C*), 118.1 (4-*C*), 107.0 (3'-*C*H), 103.5 (3-*C*H), 80.6 (9'-*C*HOC), 78.80 (8'-*C*H_2OC). 7-CH and 7'-CH are most likely exchanged to 7-CD and 7'-CD.	1
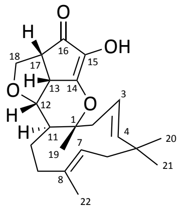 **1** Xenovulene A **101**, isolated from *A. oryzae* NSAR1 + 11 genes:	(500 MHz, CD_3OD), δ_H: 5.22 (ddd, 1H, J_{HH} = 15.9, 10.2, 2.4, 3-*H*), 5.15 (dd, 1H, J_{HH} = 15.9, 1.6, 4-*H*), 5.07 (m, 1H, 7-*H*), 3.94 (dd, 1H, J_{HH} = 9.3, 1.5, 18-*H*$_a$), 3.73 (dd, 1H, $^3J_{HH}$ = 5.1, 6.1, 12-*H*), 3.70 (dd, 1H, J_{HH} = 9.4, 8.0, 18-*H*$_b$), 3.59 (dd, 1H, $^3J_{HH}$ = 5.9, 5.9, 13-*H*), 2.93 (ddd, 1H, $^3J_{HH}$ = 7.9, 5.6, 1.5, 17-*H*), 2.61 (m, 1H, 2-*H*$_a$), 2.28 (dd, 1H, $^3J_{HH}$ = 14.9, 10.2, 2-*H*$_b$), 2.24 (br dd, 1H, $^3J_{HH}$ = 11.8, 11.4, 6-*H*$_a$), 2.13 (br dd, 1H, $^3J_{HH}$ = 10.5, 4.6, 11-*H*), 2.12 (m, 1 H, 9-*H*$_a$), 1.99 (dd, 1H, $^3J_{HH}$ = 12.5, 7.1, 1H, 9-*H*$_b$), 1.76 (br dd, 1H, $^3J_{HH}$ = 13.0, 4.9, 6-*H*$_b$), 1.63 (s, 3H, 22-CH_3), 1.49 (br ddd, 1H, J_{HH} = 13.9, 10.4, 7.1, 10-*H*$_a$), 1.42 (s, 3H, 19-CH_3), 1.29 (m, 1H, 10-*H*$_b$), 1.05 (s, 3H, 20-CH_3), 1.04 (s, 3H, 21-CH_3).		2

Supplementary Table 10. Statistics of draft genome assembly and gene prediction in *A. strictum* genome.

**Assembly statistics**		**Gene prediction**	
Bases in Scaffolds (bp)	33,842,462	# of predicted genes	10622
# of scaffolds	51	avg. gene length (bp)	1788
# of contigs	615	sense strand	5268
GC content (%)	52.28	antisense strand	5394
Avg. scaffold (bp)	663,577	avg. # of exon per gene	2.99
N50 scaffold (bp)	1,366,384	avg. exon length (bp)	535
largest scaffold (bp)	2,269,511	largest exon (bp)	11915
avg. scaf. contig (bp)	90,075	smallest exon (bp)	2
avg. contig (bp)	55,283	avg. intron length (bp)	93
N50 contig (bp)	196,596	largest intron (bp)	1887
Largest contig (bp)	618,099	smallest intron (bp)	29

Supplementary Table 11. Assignment of ORF positions for the Xenovulene BGC (Biosynthetic Gene Cluster).

#	gene, protein	bp, aa	Putative function	BLASTp[a], Phyre2[b] or CD[c]
Asg3679	*asL7*, AsL7	4350, 1449	transporter	ABC transporter
Asg3678	*asL6*, AsL6	1272, 423	oxidoreductase	FAD binding, 2-polyprenyl 6-methoxyphenol hydroxylase
Asg3677	*asL5*, AsL5	750, 249	oxidoreductase	NAD(P)-dependent short-chain dehydrogenase
Asg3676b	*asL4*, AsL4	1293, 430	oxidoreductase	FAD binding, 2-polyprenyl 6-methoxyphenol hydroxylase
Asg3676a	*asL3*, AsL3	1023, 340	oxidoreductase	Non-heme Fe^{II} dependent dioxygenase
Asg3675	*asL2*, AsL2	348, 115	hypothetical	ferredoxin like, $\alpha+\beta$ barrel
Asg3674	*asL1*, AsL1	1443, 480	oxidoreductase	FAD binding, salicylate monooxygenase
Asg3673	*aspks1*, MOS	8190, 2729	NR-PKS	3-methylorcinaldehyde synthase
Asg3672	*asR1*, AsR1	1308, 435	transporter	MFS transporter
Asg3671	*asR2*, AsR2	1551, 516	oxidoreductase	Cytochrome P450
Asg3670	*asR3*, AsR3	2565, 854	regulation	GAL4-like Zn(II)2/Cys6 binuclear cluster DNA-binding
Asg3669	*asR4*, AsR4	1899, 632	hypothetical	Zn(II)2/Cys6 binuclear cluster DNA-binding domain
Asg3668	*asR5*, AsR5	1206, 401	hypothetical	Ca-dependent phosphotriesterase (six bladed propeller)
Asg3667	*asR6*, AsR6	1293, 430	hypothetical	unknown
Asg3666	*asR7*, AsR7	2469, 822	hypothetical	Serine protease like

Supplementary Table 12. Comparison of the Xenovulene BGC (Biosynthetic Gene Cluster) and the unknown BGC from *Aspergillus thermomutatis*.

#	gene	Putative function	***aspks1*** **BGC (BIOSYNTHETIC GENE CLUSTER)**	**BLASTp[a], Phyre2[b] or CD[c]**
CDV56_06502	*atL12*	hypothetical	-	hypothetical[a]
CDV56_06501	*atL11*	transporter	-	vacuolar calcium ion transporter [a]
CDV56_06500	*atL10*	transporter	-	calcium/proton exchanger protein[a]
CDV56_06499	*atL9*	transporter	-	vacuolar calcium ion transporter [a]
CDV56_06498	*atL8*	transporter	-	sodium/calcium exchanger protein[a]
CDV56_06497	*atL7*	resistance	-	cadmium resistance transporter
CDV56_06496	*atL6*	hypothetical	-	hypothetical[a]
CDV56_06495	*atL5*	hypothetical	-	hypothetical[a]
CDV56_06494	*atL4*	hypothetical	-	hypothetical[a]
CDV56_06493	*atL3*	hypothetical	-	hypothetical[a]
CDV56_06492	*atL2*	hypothetical	-	hypothetical[a]
CDV56_06491	*atL1*	oxidoreductase	AsL1	FAD binding, salicylate monooxygenase[a,c]
CDV56_06490	*atpks1*	NR-PKS	MOS	3-methylorcinaldehyde synthase[a,c]
CDV56_06489	*atR1*	regulation	AsR3	GAL4-like Zn(II)2/Cys6 binuclear cluster DNA-binding[a,c]
CDV56_06488	*atR2*	hypothetical	AsR6	hypothetical[a]
CDV56_06487	*atR3*	hypothetical	AsR5	hypothetical[a]
CDV56_06486	*atR4*	hypothetical	-	hypothetical[a]
CDV56_06485	*atR5*	transporter	AsL7	ABC transcporter[a]
CDV56_10046	*atR6*	oxidoreductase	-	cytochrome P450 monooxygenase[a,c]
CDV56_10047	*atR7*	oxidoreductase	AsL3	Non-heme Fe^{II} dependent dioxygenase[a,c]
CDV56_10048	*atR8*	oxidoreductase	-	short-chain dehydrogenase[a,c]
CDV56_10049	*atR9*	hypothetical	-	hypothetical[a]
CDV56_10050	*atR10*	hypothetical	-	Ribonuclease like[a,c]
CDV56_10051	*atR11*	hypothetical	-	hypothetical[a]

Supplementary Table 13. **Read count after quality control and unmapped reads**. Non-producing conditions refer to fermentation conditions in which **1** is not produced; Producing conditions refer to fermentation conditions in which **1** is produced. Read counts are the total number of RNA reads from the Illumina sequencing run under the described conditios. Two biological replicates were made for each condition. See Supplementary Methods for full details.

**conditions**	**non-producing 1**	**non-producing 2**	**producing 1**	**producing 2**
read counts after filter and trimming	39.366.754	43.664.326	80.873.578	47.509.594
unmapped reads	2.431.118	2.731.955	4.407.226	1.223.507
%	6.2%	6.3%	5.0%	2.6%

Supplementary Table 14. Data calculated with DESeq for aspks1 BGC (Biosynthetic Gene Cluster) and gene boundaries.

gene	Protein name	mean expression (0.5*meanA+B)	meanA (non-producing)	meanB (producing)	Log_2-fold change (B/A)
Asg3663		1496,91	1817,58	1176,24	-0,63
Asg3664		307,23	426,31	188,15	-1,18
Asg3665		64,10	600,77	707,42	0,24
Asg3666	AsR7	5635,53	13,79	11257,28	9,67
Asg3667	AsR6	16885,47	7,78	33763,17	12,08
Asg3668	AsR5	11284,07	27,13	22541,01	9,70
Asg3669	AsR4	1899,60	14,33	3784,87	8,04
Asg3670	AsR3	6046,69	196,08	11897,30	5,92
Asg3671	AsR2	2385,73	10,81	4760,64	8,78
Asg3672	AsR1	1231,79	1,09	2462,48	11,14
Asg3673	AsPKS	12300,95	345,26	24256,64	6,13
Asg3674	AsL1	1960,75	0,00	3921,51	∞
Asg3675	AsL2	6674,83	3,28	13346,38	11,99
Asg3676a	AsL3	54766,45	63,89	109469,01	10,74
Asg3676b	AsL4	54766,45	63,89	109469,01	10,74
Asg3677	AsL5	21058,44	0,55	42116,34	16,24
Asg3678	AsL6	1962,28	0,00	3924,56	∞
Asg3679	AsL7	40698,49	5,85	81391,12	13,76
Asg3680	AsL8	450,85	46,27	855,43	4,21
Asg3681	AsL9	254,08	25,19	482,98	4,26
Asg3682		32,24	32,40	32,09	-0,01

Supplementary Table 15. Oligonucleotides used in this study.

Oligo ID	Sequence 5'-3'
11	TCCGTAGGTGAACCTGCGG
12	TCCTCCGCTTATTGATATGC
22	CTAGAAAGAAGGATTACCTC
23	CTGTCGAGAAGTTTCTGATCG
37	GCAAAGTACATCTGCGAGCG
38	CAAGAACCCCACCTCCTTCC
81	GCTCAGCAAGGTTGGGTTTG
82	CGTTGTGGATGATGTGCGTC
83	ATGGCAGCTCATGGGCAAAC
84	CACTAGAGGATCCCCATCATGCGTGTTCATGTAACGAACCCTG
85	CACATCTCCACTCGACCTGGCTCTGGCCAAGATTACCG
86	TCACAACAAGAACCCCACCTCC
124	CAGGGTTCGTTACATGAACACGCATGATGGGGATCCTCTAGTG
125	CGGTAATCTTGGCCAGAGCCAGGTCGAGTGGAGATGTG
145	CAGGAAACAGCTATGACC
146	TGTAAAACGACGGCCAGT
147	TGCTTGGAGGATAGCAACCG
148	GGGGATGACAGCAGTAACGA
179	GCCAACTTTGTACAAAAAAGCAGGCTCCGCGGAATTCATGGCAGCTCATGGGCAAAC
180	TGCCAACTTTGTACAAGAAAGCTGGGTCGGGAATTCCGTCAGGACATTGTTGGAG
181	GCCAACTTTGTACAAAAAAGCAGGCTCCGCGAATTCCTGTCGAGAAGTTTCTGATCG
182	TGCCAACTTTGTACAAGAAAGCTGGGTCGGGAATTCTCACAACAAGAACCCCACCTCC
200	CGTTCCTCGTTAAATCATCACA
201	CAATCTCTACCAGCCCACGA
202	CATTGATGAACACGCCCTTGC
203	GACGAGGATTCTGGTCGAGG
204	CCATGAAGGTCAAGATCATTGC

Oligo ID	Sequence 5'-3'
205	CTCGTCGTACTCCTGCTTGG
206	GTCCGTCTTCGACCACTGG
207	CGTAGTAGTTCTCGACACCAGG
318	GCCAACTTTGTACAAAAAAGCAGGCTCCGCATGGCAGCTCATGGGCAAAC
319	CATGTGTCCTCGCAGAAGTTG
320	CACAGAAGCAAGCACGATTC
321	TGCCAACTTTGTACAAGAAAGCTGGGTCGGTCACAACAAGAACCCCACCTC
322	CTGCTGGCTTAACACGTGC
323	GACTGGAGATAGCCGTGTGC
406	CCAGTTCTTCTCGGCGTTC
419	CCACTTCATCGCAGCTTGAC
420	AACATCGCCTCGCTCCAG
421	TTCTTTCAACACAAGATCCCAAAGTCAAAGATGGACAGCCCAGAAGTAT
422	CAGGTTGGCTGGTAGACGTCATATAATCATTTAAAGAGTATAGCCGCC
423	TTTCATTCTATGCGTTATGAACATGTTCCCTTAAAGAGTATAGCCGCC
425	CAGGTTGGCTGGTAGACGTCATATAATCATCTATGGTAGCACTACTGGC
424	ACAGCTACCCCGCTTGAGCAGACATCACCGATGGGCAGCCTCACTGAT
426	TACGACAATGTCCATATCATCAATCATGACCTATGGTAGCACTACTGGC
427	CGACTGACCAATTCCGCAGCTCGTCAAAGGATGGCTCTCGCACAGCAA
428	CAGGTTGGCTGGTAGACGTCATATAATCATTCATTCTTTGTCCGAGCG
434	GCGGCCGCATACCTAGACGCATG
435	GGTACCAGGACTTTGAAGCAGCCCT
436	GCGGCCGCATCCAACATAAGAATAA
437	GGTACCTGTGGAGGTTTGCTACTGG
555	CCTTAAAGGGACGCTGGAGG
556	CCTCCAGTTCTTCTCGGCGTTCTGG
557	CTATCGCCCATCACCCTCAC
558	CGAGACTGAGGAATCCGCTC
559	GGGTTTAAUGCGTAAGCTCCCTAATTGGC
560	GGTCTTAAUGAGCCAAGAGCGGATTCCTC
561	AGTAAGCUCGTCACCGATCTGTGAGCGCCGGCGTTTTAGAGCTAGAAATAGCAAGTTAAA
562	AGCTTACUCGTTTCGTCCTCACGGACTCATCAGACCGATCGGTGATGTCTGCTCAAGCG
563	GTGAAGGAGATGGAGCCGTC
564	GTTGCTTGATCCACAGCGTC
601	AGCTTACUCGTTTCGTCCTCACGGACTCATCAGCGCCTACGGTGATGTCTGCTCAAGCG
602	AGTAAGCUCGTCCGCCTACCTTTTCCGCCTGAGTTTTAGAGCTAGAAATAGCAAGTTAAA
603	TTTCTTTCAACACAAGATCCCAAAGTCAAAATGGCTCTCGCACAGCAATT
604	GGTTGGCTGGTAGACGTCATATAATCATACTCATTCTTTGTCCGAGCGGC
618	GCTCGACGTATTTCAGTGTC
619	GCTCCGTAACACCCAATACG
621	GCCAACTTTGTACAAAAAAGCAGGCTCCGCATGCCGCAACTAAAGGTTCT
622	CACTAGAGGATCCCCATCATGATGAGGAGCTTGCTACCGG
623	CCGGTAGCAAGCTCCTCATCATGATGGGGATCCTCTAGTG
624	GGGTTGTCCCTGCGTGTCTCAGAAGAACTCGTCAAGAAGG
625	CCTTCTTGACGAGTTCTTCTGAGACACGCAGGGACAACCC
626	TGCCAACTTTGTACAAGAAAGCTGGGTCGGTCACTCCTTGAGAAGCTCTG
627	CTGGGTTGTCCCTGCGTGTCCTATTCCTTTGCCCTCGGAC
628	GTCCGAGGGCAAAGGAATAGGACACGCAGGGACAACCCAG
629	GCCAACTTTGTACAAAAAAGCAGGCTCCGCATCAAGCGATCGTTGTAATT
630	CACTAGAGGATCCCCATCATGGCCAAGGGGAGTGGCCATT
631	AATGGCCACTCCCCTTGGCCATGATGGGGATCCTCTAGTG
632	CTGCTCGCCGGTGATCTTTCAGAAGAACTCGTCAAGAAGG
633	CCTTCTTGACGAGTTCTTCTGAAAGATCACCGGCGAGCAG
634	TGCCAACTTTGTACAAGAAAGCTGGGTCGGTTGTGTGTAGACGGACGC

Oligo ID	Sequence 5’-3’
635	CACTGCTCGCCGGTGATCTTCTATTCCTTTGCCCTCGGAC
636	GTCCGAGGGCAAAGGAATAGAAGATCACCGGCGAGCAGTG
637	GCCAACTTTGTACAAAAAAGCAGGCTCCGCATGACTGTGAAGATCCTTGT
638	CACTAGAGGATCCCCATCATGTCCGCTTCATCCTCAGACTT
639	AAGTCTGAGGATGAAGCGGACATGATGGGGATCCTCTAGTG
640	TTCTTGAACAGCGTGCTGTCAGAAGAACTCGTCAAGAAGG
641	CCTTCTTGACGAGTTCTTCTGACAGCACGCTGTTCAAGAA
642	TGCCAACTTTGTACAAGAAAGCTGGGTCGGTCATGCCTCAAACTCCAGCT
643	AATTCTTGAACAGCGTGCTGCTATTCCTTTGCCCTCGGAC
644	GTCCGAGGGCAAAGGAATAGCAGCACGCTGTTCAAGAATT
645	TTTCTTTCAACACAAGATCCCAAAGTCAAAATGCCGCAACTAAAGGTTCT
646	GGTTGGCTGGTAGACGTCATATAATCATACTCACTCCTTGAGAAGCTCTG
647	TTTCTTTCAACACAAGATCCCAAAGTCAAAATGAGCGCCATTCAAAGACT
648	GGTTGGCTGGTAGACGTCATATAATCATACTTAGATCTGAGCAAGGCCAC
649	TTTCTTTCAACACAAGATCCCAAAGTCAAAATGACTGTGAAGATCCTTGT
650	GGTTGGCTGGTAGACGTCATATAATCATACTCATGCCTCAAACTCCAGCT
653	ATGCCGCAACTAAAGGTTCT
654	TCACTCCTTGAGAAGCTCTG
655	ATCAAGCGATCGTTGTAATT
656	TTGTGTGTAGACGGACGC
657	ATGACTGTGAAGATCCTTGT
658	TCATGCCTCAAACTCCAGCT
659	AGAGGCTATTCGGCTATGAC
660	CCATGATATTCGGCAAGCAG
684	GCCAACTTTGTACAAAAAAGCAGGCTCCGCCAGGTCGAGTGGAGATGTGG
685	TAATCGCTCACCTCAACAGCATGATGGGGATCCTCTAGTG
686	CACTAGAGGATCCCCATCATGCTGTTGAGGTGAGCGATTA
687	CAGAGCTTCTCAAGGAGTGATGTGAAAGATCTAGACAAGA
688	TCTTGTCTAGATCTTTCACATCACTCCTTGAGAAGCTCTG
689	TGCCAACTTTGTACAAGAAAGCTGGGTCGGATGCCGCAACTAAAGGTTCT
690	GTGGCCTTGCTCAGATCTAATGTGAAAGATCTAGACAAGA
691	TCTTGTCTAGATCTTTCACATTAGATCTGAGCAAGGCCAC
692	TGCCAACTTTGTACAAGAAAGCTGGGTCGGATGAGCGCCATTCAAAGACT
693	AGCTGGAGTTTGAGGCATGATGTGAAAGATCTAGACAAGA
694	TCTTGTCTAGATCTTTCACATCATGCCTCAAACTCCAGCT
695	TGCCAACTTTGTACAAGAAAGCTGGGTCGGATGACTGTGAAGATCCTTGT
706	TTTCATTCTATGCGTTATGAACATGTTCCCTCACTCCTTGAGAAGCTCTG
707	ACAGCTACCCCGCTTGAGCAGACATCACCGATGAGCGCCATTCAAAGACT
708	TACGACAATGTCCATATCATCAATCATGACTTAGATCTGAGCAAGGCCAC
709	CGACTGACCAATTCCGCAGCTCGTCAAAGGATGACTGTGAAGATCCTTGT
710	TACGACAATGTCCATATCATCAATCATGACTCACTCCTTGAGAAGCTCTG
711	CGACTGACCAATTCCGCAGCTCGTCAAAGGATGAGCGCCATTCAAAGACT
755	TTCTTTCAACACAAGATCCCAAAGTCAAAATGGCTGCTCCATCACTCGAA
756	TTTCATTCTATGCGTTATGAACATGTTCCCTTTACAGTCCCTTTCTAACG
756	AACAGCTACCCCGCTTGAGCAGACATCACCATGCCTGCCTCACTCCCAGG
758	ACGACAATGTCCATATCATCAATCATGACCCTATCGCCAGTCAAAATTCG
759	GTCGACTGACCAATTCCGCAGCTCGTCAAAATGCCCGTTACTACCCCCAC
760	GGTTGGCTGGTAGACGTCATATAATCATACTTACCCAACAGCAGTTGTTA
761	GCCAACTTTGTACAAAAAAGCAGGCTCCGCATGCGTCGCAGTTTTCTTAT
762	TGCCAACTTTGTACAAGAAAGCTGGGTCGGCTAGAAGTGAAAGCCAGTCG
763	GCCAACTTTGTACAAAAAAGCAGGCTCCGCATGGGCGAGGCGTGCTCAAA
764	TGCCAACTTTGTACAAGAAAGCTGGGTCGGCTAGGCGATTTCCGCACGCT
771	GCCAACTTTGTACAAAAAAGCAGGCTCCGCATGGGCGAGGCGTGCTCAAAG
772	TGCCAACTTTGTACAAGAAAGCTGGGTCGGCTAGGCGATTTCCGCACGCTGC

Oligo ID	Sequence 5'-3'
787	TACGACAATGTCCATATCATCAATCATGACTTACAGTCCCTTTCTAACGC
788	CGACTGACCAATTCCGCAGCTCGTCAAAGGATGCCTGCCTCACTCCCAGG
789	CAGGTTGGCTGGTAGACGTCATATAATCATACCTATCGCCAGTCAAAATT
790	CGACTGACCAATTCCGCAGCTCGTCAAAGGATGCCCGTTACTACCCCCAC
791	TTTCTTTCAACACAAGATCCCAAAGTCAAAATGCCTGCCTCACTCCCAGG
792	TACGACAATGTCCATATCATCAATCATGACCTATCGCCAGTCAAAATTCG
793	TTTCTTTCAACACAAGATCCCAAAGTCAAAATGGGCGAGGCGTGCTCAAA
794	GGTTGGCTGGTAGACGTCATATAATCATACTAGGCGATTTCCGCACGCTG
795	GCCAACTTTGTACAAAAAAGCAGGCTCCGCATGCCTGCCTCACTCCCAGG
796	TGCCAACTTTGTACAAGAAAGCTGGGTCGGCCTATCGCCAGTCAAAATTC
799	ATGGGCGAGGCGTGCTCAAAG
800	CTAGGCGATTTCCGCACGCTGC
816	GAGGTCAGGGACTCGGGCATCGTCATTGGATGTGGTAACG
817	CGTTACCACATCCAATGACGATGCCCGAGTCCCTGACCTC
824	GGTTGGCTGGTAGACGTCATATAATCATACTTACAGTCCCTTTCTAACGC
825	TTTCTTTCAACACAAGATCCCAAAGTCAAAATGCCCGTTACTACCCCCAC
826	GCTTAGATTGTTTGTTAGAAGC

Supplementary Table 16. Combinations of PCR primers used for amplification of gene fragments.

Construct ID	Target	Selection marker and oligonucleotides for amplification of KO fragments	Oligonucleotides for construction in *S. cerevisiae*
RSI77 1	*asL4*	*gen*^{R}<br>P_{g+h}:P653+660<br>P_{i+j}: P659+654	P_{a+b}: P621+622<br>P_{c+d}: P623+624<br>P_{e+f}: P625+626
RSI77 2	*asL4*	*hyg*^{R}<br>P_{g+h}: P653+22<br>P_{i+j}: P23+654	P_{a+b}: P621+622<br>P_{c+d}: P623+627<br>P_{e+f}: P928+626
RSI77 3	*asL5*	*gen*^{R}<br>P_{g+h}: P655+660<br>P_{i+j}: P659+656	P_{a+b}: P629+630<br>P_{c+d}: P631+632<br>P_{e+f}: P633+634
RSI77 4	*asL5*	*hyg*^{R}<br>P_{g+h}: P655+22<br>P_{i+j}: P23+656	P_{a+b}: P629+630<br>P_{c+d}: P631+635<br>P_{e+f}: P636+634
RSI77 5	*asL6*	*gen*^{R}<br>P_{g+h}: P657+660<br>P_{i+j}: P659+658	P_{a+b}: P637+638<br>P_{c+d}: P639+640<br>P_{e+f}: P641+642
RSI77 6	*asL6*	*hyg*^{R}<br>P_{g+h}: P657+22<br>P_{i+j}: P23+658	P_{a+b}: P637+638<br>P_{c+d}: P639+643<br>P_{e+f}: P644+642

Supplementary Table 17. Construction details for yeast recombination plasmids used in this study.

Construct ID	Target	Resistance cassette	Oligonucleotides for construction in *S. cerevisiae*
RSI95 1	*asL4*	*gen*^{R}	P_{a+b}: P684+685
RSI95 2		*hyg*^{R}	P_{c+d}: P686+687
			P_{e+f}: P688+689
RSI95 2	*asL5*	*gen*^{R}	P_{a+b}: P684+985
RSI95 4		*hyg*^{R}	P_{c+d}: P868+690
			P_{e+f}: P691+692
RSI95 5	*asL6*	*gen*^{R}	P_{a+b}: P684+985
RSI95 6		*hyg*^{R}	P_{c+d}: P686+693
			P_{e+f}: P694+695

Supplementary Table 18. Construction details for fungal transformation plasmids used in this study.

Construct ID	Vector backbone	Template	Oligonucleotides for construction in *S. cerevisiae*
RSI63 1-12	RSI42A1LR4C (pTYGSarg)	RSI67-4	*asL1*: P421+422
RSI63 2-5	RSI42A1LR4C (pTYGSarg)	RSI67-4, RSI67-2	*asL1*: P421+423 *asL3*: P425+425
RSI63 4-2	RSI42A1LR4C (pTYGSarg)	RSI67-1	*asR2*: P603+604
RSI96 1-6	RSI42A1LR4C (pTYGSarg)	RSI67-4, RSI67-2, RSI67-1	*asL1*: P421+423 *asL3*: P424+426 *asR2*: P427+428
RSI96 2-9	RSI42A1LR4C (pTYGSarg)	gDNA	*asL4*: P645+706 *asL5*: P707+708 *asL6*: P709+650
RSI63 5-1	pTYGSmet	RSI67-4	*asL1*: P421+422
RSI63 6-4	pTYGSmet	RSI67-4, RSI67-2	*asL1*: P421+423 *asL3*: P425+425
RSI96 4-2	pTYGSmet	RSI67-4, RSI67-2, RSI67-1	*asL1*: P421+423 *asL3*: P424+426 *asR2*: P427+428
RSI96 5-9	pTYGSmet	gDNA	*asL4*: P645+706 *asL5*: P707+708 *asL6*: P709+650
RSI100 1-9	pTYGSmet	gDNA	*asL2*: P755+756 *asR4*: P757+758 *asR6*: P759+760
RSI97 1-1	pTYGSmet	gDNA	*asL4*: P645+710 *asL6*: P709+650
RSI97 2-2	pTYGSmet	gDNA	*asL4*: P645+710 *asL5*: P648+711
RSI97 3-1	pTYGSmet	gDNA	*asL5*: P647+708 *asL6*: P709+650
RSI112 1-1 1	pTYGSmet	gDNA	*asL2*: P755+824
RSI112 1-2 1	pTYGSmet	gDNA	*asR4*: P791+789
RSI112 1-3 1	pTYGSmet	gDNA	*asR6*: P825+760
RSI76 1-5	pTYGSade	gDNA	*asL4*: P645+646
RSI76 2-3	pTYGSade	gDNA	*asL5*: P647+648
RSI76 3-3	pTYGSade	gDNA	*asL6*: P649+650
RSI100 2-16	pTYGSade	gDNA	*asL2*: P755+756 *asR4*: P757+758 *asR6*: P759+760
RSI102 1-10	pTYGSade	gDNA	*asL2*: P755+P787 *asR4*: P788+789
RSI102 2-5	pTYGSade	gDNA	*asL2*: P755+787 *asR6*: P790+760
RSI102 3-5	pTYGSade	gDNA	*asR4*: P791+792 *asR6*: P790+760
RSI112 2-1 1	pTYGSade	gDNA	*asL2*: P755+824
RSI112 2-2 1	pTYGSade	gDNA	*asR4*: P791+789
RSI112 2-3 1	pTYGSade	gDNA	*asR6*: P825+760
RSI42A1LR4C	pTYGSarg	-	LR with RSI42A1
RSI100 9-1	RSI96 5-9 (pTYGSmet)	-	LR with RSI100 4-1
RSI108 1-1	RSI96 4-2 (pTYGSmet)	-	LR with RSI100 3-1
RSI108 2-1	RSI96 5-9 (pTYGSmet)	-	LR with RSI100 3-1
RSI108 3-1	RSI63 4-2 (pTYGSmet)	-	LR with RSI100 3-1
RSI100 6-1	RSI100 2-16 (pTYGSade)	-	LR with RSI100 3-1
RSI42A1	pE-YA	gDNA	*aspks1*: P318+319, P320+321
RSI100 3-1	pE-YA	gDNA	*asR5*: P761+762
RSI100 4-1	pE-YA	RSI100 7-9	*asR7*: P771+P772
RSI100 8-1	pE-YA	gDNA	*asR4*: P795+816, P817+796
RSI67 1	pCR2.1	cDNA	*asR2*: P427+428
RSI67 2	pCR2.1	cDNA	*asL3*: P424+425
RSI67 4	pCR2.1	cDNA	*asL1*: P421+423
RSI100 7-9	pCR2.1	gDNA	*asR7*: P799+800
		gDNA	

Supplementary Methods

Genome Sequencing and Assembly

Crude gDNA was extracted from fresh mycelia using phenol/chloroform, and then purified by caesium chloride density gradient centrifugation. Genome sequencing and assembly was performed as recently described.[3,4,5] DNA quality was assessed by gel-electrophoresis. In addition, the quantity was estimated by a fluorescence-based method using the Tecan Infinite 200 Microplate Reader (Tecan, Crailsheim, Germany) and the Quant-iT PicoGreen dsDNA kit (Invitrogen, Carlsbad, USA). For sequencing, a whole genome-shotgun PCRfree library was generated according to the manufacturer's protocols. The genome data of *Sarocladium schorii* was obtained by applying high throughput sequencing on the Illumina MiSeq platform. In total, a sequencing with 45% of a flowcell were carried out to sufficiently cover the genome. The MiSeq sequencing runs (2 × 300 bp) resulted in 14,335,458 processed reads yielding approx. 4.3 Gigabases sequence information. A *de novo* assembly of the obtained Illumina sequence reads by means of the Newbler Assembly software (version 2.8.) generated 818 contigs and 51 scaffolds accounting for a total size of 34.07 Mb. After that, genes were predicted using AUGUSTUS version 3.0.3[6] by applying a newly developed gene model for the species. Genes were functionally annotated using the genome annotation system GenDBE for eukaryotic genomes as recently described.[7,8]

Biosynthetic Gene Cluster Analysis

FungiSMASH predicted 118 BGC (Biosynthetic Gene Cluster) in the *A. strictum* draft genome. Only 39 of these BGC (Biosynthetic Gene ClusteR) were matched to a core secondary metabolite encoding gene (PKS, NRPS or terpene) and these were further investigated. The twelve predicted PKS-containing BGC (Biosynthetic Gene Cluster) formed the largest group, but also eight terpene and five NRPS-containing as well as six combined BGC (Biosynthetic Gene Cluster) were identified. Seven of the predicted clusters showed between 14-83% genes similar to previously characterised BGC (Supplementary Figure 46).

The domain organisations of all predicted PKS genes were further analysed with the conserved domain (CD) analysis tool. Ten HR-PKS, one PR-PKS, three NR-PKS and one type III PKS were annotated (Supplementary Figure 46). Only one (*Asg3673*) of the three encoded NR-PKS was found to have a *C*-Met and an R domain, which are required to release the polyketide precursor 3-methylorcinaldeyhde **4**. Sequence alignment of *Asg3673* and *aspks1* (Supplementary Figure 47) confirmed its identity (100%) and gave first *in silico* evidence that BGC 44 (referred to as *aspks1* BGC) is the best candidate for xenovulene A **1** biosynthesis. This cluster also posseses 14% of genes similar to the stipitatic acid (tropolone) cluster.

Upload of Annotated Genome and BGC to Genbank

The *Sarocladium schorii* genome sequence was deposited in the EMBL database under the Bioproject ID PRJEB23865, as well as the accession numbers OKRM01000001-OKRM01000051 (Scaffolds). The fully annotated 49 Kb BGC (Biosynthetic Gene Cluster) is uploaded to Genbank with the accession number MG736817

Bioinformatic Analysis

ORFs were determined using transcriptome data. Translated proteinsequences were analysed using BLAST, Phyre-2 and conserved domain analysis (Supplementary Table 11). Further details are given below.

AsR5: A Model of AsR5 computed with SWISS-MODEL and template 2p4o.1.A (hypothetical protein of *Nostoc punctiforme* PCC 73102, sequence homology 21.22% to AsR5). See Supplementary Figure 55.

AsR6: Alignment of amino acid sequences of class I terpene cyclases to show conserved motifs DDxxD/E and (N)DxxT/SxxxD/E. Humulene synthase from *Z. zerumbet* (AB247331.1), *F. fujikorii* koraiol synthase Ffsc4 (CCT72694.1), *C. acutatum* CaTPS (KP398851.1) and aristolochene synthase from *A. terreus* (AAF13264.1) and AsR6. Geneious 7.1.9 was used for the alignment. Mg^{2+} binding residues highlighted in red in Supplementary Figure 70. Blast searches revealed at least 11 close homologs of AsR6 (Supplementary Figure 56), all of which have no known function.

Cluster Comparison: AsR6, which encodes the terpene cyclase is homologous to several other unknowns in the database. In the case of *atR3* (06487) from *Aspergillus thermomutatus* the gene occurs in a cluster which could encode the biosynthesis of a tropolone meroterpenoid as it contain PKS (06490), TropB (06491) and TropC (*atR7* 100457) genes sufficient to make a tropolone and genes including *atR2* (06488) and *atR3* (06487) homologous to *asR6* and asR5 shown here to construct humulene and link it to a tropolon (Supplementary Figure 57 and Supplementary Table 12).

Transcriptome Sequence

Xenovulene A **1** production medium (ASPM) is well known from preliminary experiments.[2] To identify **1** non-producing conditions the secondary metabolite production was analysed in different liquid media. DPY medium was found to suppress **1** production but did not inhibit the growth of *A. strictum* (Supplementary Figure 3.5). Therefore, it was selected as non-producing medium for comparative transcriptome analysis. Liquid cultures for both conditions (ASPM, DPY) were inoculated with an *A. strictum* single colony and cultured for nine days. Samples of two biological replicates for each condition were taken at day 3, 6 and 9 and combined for total RNA isolation by kit (Zymo Research). See Supplementary Figure 48.

Transcriptome analysis was performed as recently described.[9] In brief, sequencing of the two prepared cDNA libraries was carried out on the Illumina HiSeq 1500 platform (2 x 55bp, Illumina Inc., San Diego, U.S.A.). In total, 184 million sequence reads (on average approx. 46 million reads for each of two replicates per condition) amounting to 10 Gb sequence information were generated for all transcriptome libraries. Data analysis and base calling were accomplished with in-house software based on CASAVA 1.8.2. (Illumina).[8] The obtained reads were quality filtered (> Q30) using the FASTX tool kit (http://hannonlab.cshl.edu/fastx_toolkit/).[10] Data of all two replicates of each condition were mapped to the *Sarocladium schorii* genome using tophat2 with default settings. Results of the short read mapping were imported into the platform ReadXplorer 2.2.3.[11,12] For Gene expression analysis, the R package DESeq[13] was selected. Only genes with a minimum fold change of $\log_2$ 1, as well as a p-adjusted value smaller than 0.05 were deemed significant. In addition, reads per kilobase per million reads (RPKM) values were calculated. On average 95% of those were mapped to the *A. strictum* draft genome (Supplementary Tables 13-14). The *Sarocladium schorii* transcriptome data was deposited in the EMBL database under the Bioproject ID PRJEB23865 (See Supplementary Table 13).

The sequenced transcriptomes for non-producing (A) and producing (B) conditions were used to perform a differential expression sequence (DESeq) analysis.[13] DESeq is a software package that uses statistical testing and a negative binominal distribution to compare differences in observed read counts. It evaluates whether the observed differences in two biological samples are significant or in the range of natural variation. The mean expression (meanA or meanB) of non-producing (A) and producing (B) conditions is calculated from the normalized read counts of biological replicates (in this work two per condition). The normalized read count is then used to calculate the relative gene expression

(fold change, meanB over meanA, Supplementary Figure 49). Comparing the $\log_2$-fold change of selected genes their up- ($\log_2$-fold change>0) and downregulation ($\log_2$-fold change<0) can be determined. If a $\log_2$-fold change of >2 and an adjusted p-value >0.05 (significant differential expression determined by Benjamini-Höchberg[14] testing with a false discovery rate of 5%) was observed, genes were regarded as differentially expressed. These genes are displayed in red (Supplementary Figure 49); genes displayed in black were regarded as not differently expressed. Genes with large or infinite $\log_2$-fold change are displayed at the borders of the scatter diagram.

The expression of the *aspks1* gene cluster (green dots, Supplementary Figure 49.) was analysed by comparing the $\log_2$-fold change of genes (*Asg3663-Asg3682*) encoded ~25 kb up- and downstream of *aspks1* (Supplementary Figure 49 and Supplementary Tables 13-14). The $\log_2$-fold change of genes between *Asg3666-3679* was found to be between 6 and 16. For *Asg3674* and *Asg3678*, no reads under non-producing conditions were assembled (meanA = 0), which resulted in an infinite $\log_2$-fold change. This analysis allowed to set cluster boundaries of the *aspks1* BGC to a 16 (*Asg3666-Asg3679*) genes encoding, 48922 bp spanning genomic region. Three investigated genes adjacent to either 5' (*Asg3663-65*) or 3' (*Asg3680-82*) cluster borders (orange dots, Supplementary Figure 49), were found to be not differentially expressed (Supplementary Table 14).

Cloning, Expression, Purification and Assay of AsR5 and AsR6

For expression of *asR6* and *asR5* in *E. coli*, an expression plasmid (pET100/D-TOPO) carrying the *E. coli* codon-optimized *asR6* or *asR5* gene cassette respectively with an N-terminally fused His_6-Tag was obtained from Invitrogen (GeneArt Gene Synthesis).

Following transformation into chemically competent *E. coli* BL21 (DE3) cells, a single colony was inoculated into 5 mL LB medium supplemented with 50 $\mu g \cdot mL^{-1}$ carbenicillin and grown overnight at 37 °C and 225 rpm.

An 0.5 mL aliquot of this seed culture was used to prepare a glycerol stock, whereas the remaining culture was inoculated into 2L flasks containing 1L 2TY medium supplemented with 50 $\mu g \cdot mL^{-1}$ carbenicillin. Cells were incubated at 37 °C and 200 rpm until A_{600} reached values between 0.4 and 0.6. To induce protein expression Isopropyl β-D-thiogalactopyranoside (IPTG) was added to a final concentration of 0.1 mM and the cells were incubated at 12 °C (AsR6)/16 °C (AsR5) and 200 rpm for 19 h.

Cells were harvested by centrifugation (3500 × g, 20 min) at 4 °C and then resuspended in loading buffer (50 mM Tris-HCL (pH = 8.0), 150 mM NaCl, 20 mM imidazole, 10% glycerol) and lysed by sonification. Cell debris was removed from the total lysate by centrifugation (12.000 × g, 1 h, 4 °C).

To purify AsR6 or AsR5 respectively from the total lysate an Äkta Pure FPLC system (*GE Healthcare*) connected to a HisTrap™-column (*GE-Healtcare*, column volume = 5 mL) was used. The FPLC was programmed to equilibrate the column with 5 Column Volumes (CV) loading buffer (at 5 mL/min) prior to injecting the sample upon the column (at 1 $mL \cdot min^{-1}$). The column was then washed with 6 CV loading buffer. Elution was achieved using a gradient (buffer A: loading buffer; buffer B: Elution buffer (50 mM Tris-HCl (pH = 8.0), 150 mM NaCl, 500 mM imidazole, 10% glycerol)); the concentration of buffer B was raised from 0-100% across 2 CV, followed by 3 CV of 100 % buffer B and 2 CV of 100% buffer A. Elution of protein was monitored using a UV-detector (280 nm). Eluting fractions containing protein of interest were pooled, concentrated using an Amicon Ultra-15 Centrifugal Filter Unit (*Millipore;* cut-off: 30 kDa) and rebuffered into storage buffer (50 mM Tris-HCl (pH = 8.0), 150 mM NaCl, 10% glycerol) using a HiPrep™ 26/10 Desalting column (*GE Healthcare*).

In order to improve the purity of asR6 for the use in *in vitro* assays the protein was further purified via anion exchange and size-exclusion chromatography. Anion exchange was done using a HiTrap Q FF column (*GE-Healthcare*, column volume = 5 mL). For this, asR6 was

diluted 1:3 with anion exchange buffer A (50 mM NaCl, 50 mM Tris (pH = 8.0), 10% glycerole). The FPLC was programmed to first equilibrate the column with 5 CV of anion exchange buffer A (at 5 mL·min^{-1}). The protein sample was then injected at 1 mL·min^{-1}. After a wash step (5 CV of anion exchange buffer A at 2.5 mL·min^{-1}) the protein was gradually eluted by increasing the salt concentration. For this, the concentration of anion exchange buffer B (1.5 M NaCl, 50 mM Tris (pH = 8.0), 10% glycerole) was raised from 0-35% across 7 CV, then from 35-65% across 4 CV, then from 65-100% across 4 CV and kept at 100% anion exchange buffer B for 2 additional CV.

Eluting fractions containing asR6 were concentrated to a sample volume of 1 ml using an Amicon Ultra-15 Centrifugal Filter Unit and injected upon a HiLoad® 26/600 Superdex® 200 pg (*GE Healthcare*, column volume = 320 ml) size-exclusion column previously equilibrated with storage buffer (50 mM Tris-HCl (pH = 8.0), 150 mM NaCl, 10% glycerol). The flow rate was set to 1 mL·min^{-1}.

100 µL aliquots of the eluted protein were stored at -20 °C. Purified proteins were assessed using SDS-PAGE (Supplementary Figure 58) and were confirmed by mass spectrometry (Dr. Jennifer Senkler, AG Braun, Leibniz Universität Hannover) using the methods of Klodmann and coworkers.[15]

E. coli Codon Optimised Sequences and ESI-Q-TOF-MS

AsR5:

5′-ATGCGTCGTAGCTTTCTGATTAGCGCAGCACTGGGTCTGAGCATGAGCACACCGGCACTGGCAGCAAGCATTCAGAGCGTTCTGGGTTATC
TGCGTCCGACCAGCCATCATCATGCACCGTGTGCCGATGATGTTGTTCTGAAACAGAGCGCAGGTAGCGATAGTGCAGCACCGGATCCGCTGCCGAG
CCGTGTTGTTCATAATTGGCCGAATGGCACCTGGATTGAAAACATTAGCGTTCGTCCGAATGGTAATCTGCTGGTTAGCCAGAGTACACCGCGTGGTC
GTGTTTGGCAGGTTAAAGAACCGTGGCTGGATGAACCGAAAGTTGAACTGGCATATGATTTTGATGAATGGGTTGATCGCATTATTGGTATTGGTGA
AACCACACCGGATAAATATGTTGTTGTTGGTAGCCGTTTTTATAGCCTGGATCCGCAGAGCAGCCAGGTTGAACGTACCTTTTGTGCAATGGAACTGG
ATTTTACCAAAGGTGAAAAACCGAGCGCACGTCTGGTTGCACGTTTTCCGCATGCAAATCTGCTGCAGAGCGTTAGCGCACTGCCGTGGGATCGTAGC
GTGGTTCTGATTTCAGATCAGTACCTGCTGCATCCGCGTGCAGATTGGGAAGATCTGACCCCTGGTCCGGGTCAGATTTGGCGTCTGGATACCAAAAC
CGGTCATCATGAAATTGTGATGACCAACTATGCCGAAATGAACACCACCTATAATCATGGTCTGGATGTGGGTATTAACGGCATTAAAATCCATGGCG
ATCATCTGTATTGGATCAATATGGATACCGGTGGTGCGTATCGTGTTCGCATTGATAAATACGGTTATCCGACACCGCTGAATGCAGTTCCGGAAACA
CTGGGTGTTGCCGAAGATGCACTGTGGGATGATTTTGCAATGCATGGCACCCGCATTGGTGAAGAAAGTGATGATACCACCATGTTTGCAACCAGCA
TTGTTAATCTGATGGCAATTAGTCCGGAAAATGGCACCATTGTTCCGCTGGCAGGCGTTGGCACCAGCGAACCGATGGGTTTTCCGGGTCCGACCTCA
GCACAGTTTGGTCGTACCGAAAAAGATAGCCATATTCTGTATGTGACCGGCAAACTGTTTAATGTTCCGCCTAGCATTCGTGATGTTGTGATTCAAGGT
TGGGTTCGTGCAATTGATACCACGGGTTTTCACTTTTAA-3′

AsR6:

5′-ATGCCGGTTACCACACCGACCAAAATGGCAACCCTGACCACCAAACAAATGTGGCAGACCATCAAAGATTATTTTGGTGATGGTTTTGTTAC
CGGTAGCGCACCGATTAGCTATAACGTTCATACCTGTGATATGCAACTGCAGCCGGATAGCGGTATTCATGCAGCAAGTGATGGTATTCATTATGGTG
TTCAGATTAGCGAAGATAGCATGCCGCTGTTTAGCATTATGGGTGATACCGCAGCACCGCCTTGTACCTGTCATCGTGTTGATGAAATTGTGAAACAC
ATCGACGAATTTCTGGAACGTGCACCGGAAGCACTGCCGGATGATGGTGCAATTACCAGCGGTAAACCGTGTGATACCAATCCGGATCAGGTTAGCC
TGTATGCAATGCGTGATAGCCTGAGTTGGTGGGTTCATTGGGGTGGTAATCTGCGTCCGGAACATTATTGGAAGCAGATTTATATCGGTTTTGCAGCC
ATTCCTGATGATGTGCAGATTAGTCCGCGTGAATTCCTGGATGGCACCTATCGTTATCTGGGTCATACCTGGGATGATTGTCTGAGCGGTCTGGAAGA
AGAGGGCGTTAGTCCGGATGAAATCGAATTTGCCAATATGTGTATGTGGCGTCAGATGCTGACCCAGTGGCTGGAAAAAGCAGATCCTGAACTGCTG
CCGCTGCTGAAAGGTAAAATTAGCCTGATGCTGCAGTATCGTGTTCTGACCGCAAATACCCTGGGTTGTCTGGCACTGTTTATGAATGCAACCGCAGA
TCCGAAAGATGGTCCGATCCATTATGCAGATAGCAGCTATGAAATGGAAATTGCAAGCGTTGCACAGTGTGTTACCCTGGATATGGCAAAAGAAGCA
ATGGGTATTCTGCAGGGTGAACGTACCGAAGTTGTTGCCGGTGATCGTGCACAGCGTAAACGTGAACTGCGTTGGATTTATGTTCGTTGTATGCAGAT
TCTGGAAAGCCAGCCGCATGCACATATGCTGCGTCGTTATGGTAGCGCAGGTCTGCATTATGTTCCGATGATGGATCGCTATCTGGAACGCGTTAGCG
GTCATACCCGTTTTCCGATTCGTGATGGTGCCGCACGTATCCTGGAACGTTTTATCAATCGTGCAGAACTGCCGAAAGAAAGCGAGGATATTAATCCG
AATGGTCGTAGCCTGAAAGTTAGCGCAAAAATGAATGGTAATGGTCAGCTGCATCATGAAGTTAATGGCAATGCAAAACTGCATCTGGAAGCCGAAC
GTCCGGATGTTACCACCGCAGTTGGTTAA-3′

ESI-Q-TOF analysis of AsR5 and AsR6

Protein sequence of **AsR5** with *N*-terminal His$_6$-Tag. Red residues were observed as part of peptide fragments by ESI-Q-TOF-MS (Coverage 70%):

MRGSHHHHHHGMASMTGGQQMGRDLYDDDDKDHPFTMRRSFLISAALGLSMSTPALAASIQSVLGYLRPTSHHHAPCADDVVLKQSAGSDSAAPDPLPSR
VVHNWPNGTWIENISVRPNGNLLVSQSTPRGRVWQVKEPWLDEPKVELAYDFDEWVDRIIGIGETTPDKYVVVGSRFYSLDPQSSQVERTFCAMELDFTK
GEKPSARLVARFPHANLLQSVSALPWDRSVVLISDQYLLHPRADWEDLTPGPGQIWRLDTKTGHHEIVMTNYAEMNTTYNHGLDVGINGIKIHGDHLYWI
NMDTGGAYRVRIDKYGYPTPLNAVPETLGVAEDALWDDFAMHGTRIGEESDDTTMFATSIVNLMAISPENGTIVPLAGVGTSEPMGFPGPTSAQFGRTEK
DSHILYVTGKLFNVPPSIRDVVIQGWVRAIDTTGFHF

Protein sequence of **AsR6** with *N*-terminal His$_6$-Tag. Red residues were observed as part of peptide fragments by ESI-Q-TOF-MS (Coverage 66%):

MRGSHHHHHHGMASMTGGQQMGRDLYDDDDKDHPFTMPVTTPTKMATLTTKQMWQTIKDYFGDGFVTGSAPISYNVHTCDMQLQPDSGIHAASDGIHYGV
QISEDSMPLFSIMGDTAAPPCTCHRVDEIVKHIDEFLERAPEALPDDGAITSGKPCDTNPDQVSLYAMRDSLSWWVHWGGNLRPEHYWKQIYIGFAAIPD
DVQISPREFLDGTYRYLGHTWDDCLSGLEEEGVSPDEIEFANMCMWRQMLTQWLEKADPELLPLLKGKISLMLQYRVLTANTLGCLALFMNATADPKDGP
IHYADSSYEMEIASVAQCVTLDMAKEAMGILQGERTEVVAGDRAQRKRELRWIYVRCMQILESQPHAHMLRRYGSAGLHYVPMMDRYLERVSGHTRFPIR
DGAARILERFINRAELPKESEDINPNGRSLKVSAKMNGNGQLHHEVNGNAKLHLEAERPDVTTAVG

Construction of *A. oryzae* Expression Vectors

KO of *aspks1* in *A. strictum* confirmed the involvement of the *aspks1* BGC (Biosynthetic Gene Cluster) in **1** biosynthesis. Differential expression analysis showed the upregulation of 15 genes (*asL1-7*, *asR1-7* and *aspks1*). For heterologous co-expression 12 genes were selected, omitting only transporters (*asR1*, *asL7*) and transcription factors (*asR3*) (Supplementary Figure 60).

The biosynthetic hypothesis suggested that early tropolone formation is similar to *T. stipitatus*. Thus, for co-expression of *aspks1*, *asL1*, *asL3* and *asR2*, the genes were cloned into the pTYGSarg vector.[16] For sequential co-expression of these genes, different combinations (*aspks1*, *aspks1*+*asL1*, *aspks1*+asL1+*asL3* and *aspks1*+*asL1*+*asL3*+*asR2*) were also cloned into pTYGSarg (Supplementary Tables 15-18). The three additional oxidoreductases encoded by *asL4*, *asL5* and *asL6*, which could be involved in the ring contraction, were cloned into pTYGSmet.[16] The genes encoding proteins of unknown function (*asL2*, *asR4*, *asR5* and *asR6*) were cloned into pTYGSade.[16] For co-expression of different sub-sets of genes, combinations of two genes each (*asL4*, *asL5* and *asL6* or *asL2*, *asR4* and *asR6*) were cloned into pTYGSmet and pTYGSade (Supplementary Figure 61).

All pE-YA entry and pTYGS(arg/ade/met) vectors for expression of the *aspks1* BGC (Biosynthetic Gene Cluster) were constructed by *in vivo* homologous recombination in *S. cerevisiae* and *in vitro* recombination. Genes were amplified by PCR using *A. strictum* c- or gDNA as template, with oligonucleotides featuring 30 bp homologous overlaps. The 8190 bp *aspks1* gene was amplified in two fragments (each ~4100 bp) with 70 bp overlap from *A. strictum* gDNA and assembled in the gateway entry vector pE-YA.[16]

Genes amplified from cDNA (*asL1*, *asL3* and *asR2*) were initially sub-cloned in pCR2.1 by the TOPO TA cloning kit (Invitrogen). Correct splicing was confirmed by sequencing (Eurofins, Ebersberg) and the vector used as template in further PCR reactions. Assembly of 1 or 2 genes in pTYGS expression vectors was achieved by designing oligonucleotides with an overlap to T_{adh}/P_{gpdA} or T_{gpdA}/P_{eno} and P_{eno},[16] which resulted in the removal of the 2480 bp T_{adh}/P_{gpdA} or the respective 800 bp T_{gpdA}/P_{eno} DNA fragment. All entry and expression clones used for LR recombination and subsequent *A. oryzae* NSAR1 transformation are displayed in Supplementary Figure 61.

Supplementary References

1. Davison, J. *et al.* Genetic, molecular, and biochemical basis of fungal tropolone biosynthesis. *Proc. Nat. Acad. Sci. USA* **109,** 7642–7647 (2012).
2. Ainsworth, A. *et al.* Xenovulene-a, a Novel Gaba-Benzodiazepine Receptor-Binding Compound Produced by *Acremonium-Strictum J. Antibiot.,* **48,** 568–573 (1995).
3. Wibberg, D. *et al.* Improved genome sequence of the phytopathogenic fungus Rhizoctonia solani AG1-IB 7/3/14 as established by deep mate-pair sequencing on the MiSeq (Illumina) system. *Journal of Biotechnology* **203,** 19–21 (2015).
4. Wibberg, D. *et al.* Draft genome sequence of the potato pathogen Rhizoctonia solani AG3-PT isolate Ben3. *Arch Microbiol* **199,** 1065–1068 (2017).
5. Jahn, L. *et al.* Linking secondary metabolites to biosynthesis genes in the fungal endophyte Cyanodermella asteris : The anti-cancer bisanthraquinone skyrin. *Journal of Biotechnology* **257,** 233–239 (2017).
6. Stanke, M., Schöffmann, O., Morgenstern, B. & Waack, S. Gene prediction in eukaryotes with a generalized hidden Markov model that uses hints from external sources. *BMC Bioinformatics* **7,** 62 (2006).
7. Rupp, O. *et al.* Construction of a Public CHO Cell Line Transcript Database Using Versatile Bioinformatics Analysis Pipelines. *Plos One* **9,** e85568–13 (2014).
8. Wibberg, D. *et al.* Genome analysis of the sugar beet pathogen Rhizoctonia solani AG2-2IIIB revealed high numbers in secreted proteins and cell wall degrading enzymes. *BMC Genomics* 1–12 (2016). doi:10.1186/s12864-016-2561-1
9. Verwaaijen B, *et al*. The *Rhizoctonia solani* AG1-IB (isolate 7/3/14) transcriptome during interaction with the host plant lettuce (*Lactuca sativa L.*). PLoS One. **12,** e0177278 (2017).
10. Kim, D. *et al.* TopHat2: accurate alignment of transcriptomes in the presence of insertions, deletions and gene fusions. *Genome Biol.* **14,** R36 (2013).
11. Hilker, R. *et al.* ReadXplorer - Visualization and analysis of mapped sequences. *Bioinformatics* **30,** 2247–2254 (2014).
12. Hilker, R. *et al.* ReadXplorer 2-detailed read mapping analysis and visualization from one single source. *Bioinformatics* **32,** 3702–3708 (2016).
13. Anders, S. & Huber, W. Differential expression analysis for sequence count data. *Genome Biol.* **11,** R106 (2010).
14. Benjamini, Y. & Hochberg, Y. Controlling the False Discovery Rate - a Practical and Powerful Approach to Multiple Testing. *Journal of the Royal Statistical Society Series B-Methodological* **57,** 289–300 (1995).
15. Klodmann, J., Senkler, M., Rode, C. & Braun, H.-P. Defining the protein complex proteome of plant mitochondria. *Plant Physiol* **157,** 587–598 (2011).
16. Pahirulzaman, A. K., Williams, K. & Lazarus, C. M. A toolkit for heterologous expression of metabolic pathways in *Aspergillus oryzae*. *Meth. Enzymol.* **517,** 241–260 (2012).
